# Impact of Age on the Cerebrovascular Proteomes of Wild-Type and Tg-SwDI Mice

**DOI:** 10.1371/journal.pone.0089970

**Published:** 2014-02-26

**Authors:** James L. Searcy, Thierry Le Bihan, Natalia Salvadores, James McCulloch, Karen Horsburgh

**Affiliations:** 1 Centre for Neuroregeneration, University of Edinburgh, Edinburgh, United Kingdom; 2 SynthSys – Synthetic & Systems Biology, University of Edinburgh, Edinburgh, United Kingdom; 3 Institute of Structural and Molecular Biology, University of Edinburgh, Edinburgh, United Kingdom; 4 Centre for Cognitive and Neural Systems, University of Edinburgh, Edinburgh, United Kingdom; 5 Centre for Cognitive Ageing and Cognitive Epidemiology, University of Edinburgh, Edinburgh, United Kingdom; Massachusetts General Hospital/Harvard Medical School, United States of America

## Abstract

The structural integrity of cerebral vessels is compromised during ageing. Abnormal amyloid (Aβ) deposition in the vasculature can accelerate age-related pathologies. The cerebrovascular response associated with ageing and microvascular Aβ deposition was defined using quantitative label-free shotgun proteomic analysis. Over 650 proteins were quantified in vessel-enriched fractions from the brains of 3 and 9 month-old wild-type (WT) and Tg-SwDI mice. Sixty-five proteins were significantly increased in older WT animals and included several basement membrane proteins (nidogen-1, basement membrane-specific heparan sulfate proteoglycan core protein, laminin subunit gamma-1 precursor and collagen alpha-2(IV) chain preproprotein). Twenty-four proteins were increased and twenty-one decreased in older Tg-SwDI mice. Of these, increases in Apolipoprotein E (APOE) and high temperature requirement serine protease-1 (HTRA1) and decreases in spliceosome and RNA-binding proteins were the most prominent. Only six shared proteins were altered in both 9-month old WT and Tg-SwDI animals. The age-related proteomic response in the cerebrovasculature was distinctly different in the presence of microvascular Aβ deposition. Proteins found differentially expressed within the WT and Tg-SwDI animals give greater insight to the mechanisms behind age-related cerebrovascular dysfunction and pathologies and may provide novel therapeutic targets.

## Introduction

The structural integrity of the cerebrovasculature is altered during ageing. These changes can be accelerated by vascular pathologies, most notably cerebral amyloid angiopathy (CAA) [1], [2], [3], [4], [5], [6], [7]. Ageing alone can lead to vessels supplying deep white matter to become tortuous. Thickening of the veins and venules is also commonly observed along with increased thickening of the basement membrane [1]; [8]. A decline in vascular density, specifically capillary loss, has been observed in both the elderly and aged animal models [9], [10] along with increases in arteriole and capillary diameter [11]. Many of these same age-related pathologies are recapitulated in brains with CAA, which is relatively common in the elderly population, presenting in 10–40% of the non-demented individuals over the age of 60 [12]. CAA can be categorized into two types identified pathologically by the accumulation of Aβ in leptomeningeal and cortical vessels including capillaries (Type 1) or with the exception of capillaries (Type 2) [13], [14]. CAA can lead to the loss of smooth muscle cells and thickening of the vessel walls, which in turn causes the blood vessel to become compromised, leading to intracerebral hemorrhage [15].

The presence of Aβ leads to very different protein changes that are independent of age. This includes the modulation of proteins that are involved in movement, aggregation and degradation of Aβ. APOE is a protein that is intimately involved in all of these processes [16]: the expression of the APOE4 allele leads to a propensity for increased Aβ aggregation in sporadic AD [17] and increased risk of Aβ deposition in capillaries [13]. APOE plays a direct role in Aβ efflux across the blood brain barrier to circulating plasma [18], [19], and its overexpression promotes Aβ proteolysis via enzymes such as neprilysin and insulin degrading enzyme [20]. Overexpression of neprilysin and insulin degrading enzyme decrease Aβ levels in transgenic amyloid precursor protein (APP) mouse models and improve cognition [21].

The Tg-SwDI transgenic mouse model of amyloidosis expresses human APP with three different mutations: the Swedish, Dutch and Iowa mutations. The double Swedish mutation leads to increased production and secretion of Aβ [22]. The Dutch and Iowa mutations, found in independent familial forms of CAA, increase the propensity for Aβ to accumulate in the vessels and are associated with cortical hemorrhages [23]. The Tg-SwDI model expresses the human APP gene at levels similar to endogenous mouse APP levels [23], and yet develops a temporal accumulation of Aβ. This latter accumulation of Aβ is potentially attributed to properties of the mutant Aβ preventing normal clearance [24].

As the Aβ accumulates over time, it associates closely with microvessels, (seen in Type 1 CAA) and is primarily fibrillar in nature [23], [25]. This microvascular deposition is closely associated with an increased inflammatory response [23]. These alterations observed in the Tg-SwDI, resulting from advancing age and accumulating Aβ, should be underpinned by changes in various proteins, including those associated with the neurovascular unit and the blood brain barrier [7], [26], [27], [28], [29] yet the scope of these changes are not fully understood.

To better understand how cerebral vessels globally respond to both age and accumulating Aβ, we have used a quantitative label-free proteomics approach to investigate the proteomic signatures of vessel-enriched fractions from the brains of WT and Tg-SwDI mice at 3 and 9 months of age. Our vascular-focused approach has revealed distinctly different protein expression patterns as a result of age in the presence of microvascular Aβ accumulation.

## Materials and Methods

### Ethics Statement

All procedures were authorized under the Home Office approved project license number 60/3722. This license was approved by the University of Edinburgh’s Ethical Review Committee and the Home Office and adhered to regulations specified in the Animals (Scientific Procedures) Act (1986).

### Animals

Tg-SwDI mice were obtained from Jackson Laboratories (Bar Harbour, MA). These mice were generated and previously described [23] on a C57Bl/6J background. Homozygous Tg-SwDI mice were bred and maintained. C57Bl/6J (WT) mice were obtained from Jackson laboratories through their UK distributor Charles River.

### Vessel Enrichment

Vessels were enriched using a protocol based on 2 sources [30], [31]. Hemibrains were homogenized in 1 mL of ice-cold PBS using a loose fit dounce homogenizer using 15 strokes. Homogenates were transferred to a 15 mL falcon tube and centrifuged for 10 minutes at 250×g. The pellet was re-suspended in 17.5% Ficoll solution (Sigma) and centrifuged for 25 minutes at 3,200×g to collect the pellet (vessel containing S1 fraction), which was retained on ice. The supernatant was centrifuged again for 25 minutes at 3200×g to collect a second pellet and the pellets from both spins were combined in 1 mL of 1% BSA-PBS and re-suspended. The suspension was centrifuged for 10 minutes at 2000×g. The pellet was subsequently washed in 1 mL of ice-cold PBS before being flash-frozen on dry ice and later stored at −70°C until further processing.

### Immunoblots

S1 fractions obtained from hemibrains of 3 month-old WT mice (n = 3) for vessel enrichment confirmation were homogenized in a lysis buffer of 9 M Urea/4% CHAPS with Complete Protease Inhibitor Cocktail (Roche, Manheim, Germany). Protein levels were determined using a Pierce BCA protein assay kit (Thermo Scientific, Cramlington, Northumberland, UK). Proteins were separated by SDS–PAGE and transferred onto nitrocellulose membranes.

S1 fractions obtained from hemibrains of 3, 12 and 17 month-old Tg-SwDI mice (n = 4/group) for APOE and HTRA1 confirmation were homogenized in a sucrose buffer with Complete Protease Inhibitor Cocktail (Roche, Manheim, Germany). Protein levels were determined as above and proteins were separated by SDS–PAGE and transferred onto PVDF membranes (Bio-Rad, Germany).

Immunoblotting was performed using the Odyssey Infrared Imaging System (LiCor Biosciences, Lincoln, NE, USA). Membranes were blocked in Odyssey blocking buffer (diluted 1∶1 with phosphate-buffered saline) and washed in phosphate-buffered saline–Tween® (phosphate-buffered saline with 0.1% Tween). Primary antibodies were detected using fluorescently labelled secondary antibodies (LiCor Biosciences). Proteins were visualized by scanning antibody-labelled blots in the Odyssey Imager under the appropriate channel. Antibodies for immunoblotting were as follows: Platelet endothelial cell adhesion molecule (PECAM), smooth muscle actin (SMA), glial fibrillary acid protein (GFAP) (AbCam, Cambridge, UK), Occludin (Invitrogen, Camarillo, CA, USA).

### Cerebral Vessel Aβ Levels

Total Aβ was extracted from independent S1 fractions taken from independent cohorts of 3, 12 and 17 month-old Tg-SwDI mice (n = 4/group) using a homogenisation buffer of the following constituents: 5 M guanidine and 50 mM Tris/HCL and mixed for 3–4 hours at RT and later stored at −20°C. The solution was diluted 1∶10 in ice-cold reaction buffer of the following constituents: 0.2 g/L KCL, 0.2 g/L KH2PO4, 8 g/L NaCl, 1.15 g/L Na2HPO4, 5% BSA, 0.03% Tween 20 and 1X protease inhibitor cocktail (Calbiochem). The solution was centrifuged at 16,000×g for 20 minutes at 4°C. Protein levels were quantified with Pierce BCA Protein Assay Kit (Thermo Scientific). Aβ1-40 levels were measured using the Invitrogen Human Ultrasensitive ELISA kit (Life Technologies, Paisley, UK) following the manufacturer’s instructions.

### LC-MS Analysis of Vessel-enriched Fractions

All chemicals were purchased from Sigma-Aldrich (UK) unless otherwise stated. Acetonitrile and water for LC-MS/MS and sample preparation were HPLC quality (Fisher, UK). Formic acid was Suprapure 98–100% (Merck, Darmstadt, Germany) and trifluoroacetic acid was 99% purity sequencing grade. Trypsin and Lys-C (modified, sequencing grade) were purchased from Roche Diagnostics (West Sussex, UK). All HPLC-MS connector fittings were from Upchurch Scientific or Valco (Hichrom and RESTEK, UK).

100 *µ*g of protein extract from the vessel-enriched fractions from 3 and 9 month-old, Tg-SwDI and WT mice (n = 7–8/group) was re-suspended in 45 *µ*L of 8 M urea, 2.5 *µ*L of 200 mM DTT and 5 *µ*L of 1 M ammonium bicarbonate. The samples were reduced at room temperature for 30 minutes, and then 2.5 *µ*L of 500 mM iodoacetamide was added. 0.5 *µ*g of Lys-C was added and digested overnight in 6 M urea followed by dilution to 2 M urea and overnight digestion with 2 *µ*g of trypsin.

Capillary-HPLC –MSMS data were collected on an online system consisting of a micropump (1200 binary HPLC system, Agilent, Edinburgh, UK) coupled to a hybrid LTQ-Orbitrap XL instrument (Thermo-Fisher, Loughborough, UK). HPLC-MS methods have been described previously [32]. Peptides were reconstituted in 10 *µ*L of loading buffer before injection and 8 *µ*L was loaded. The peptide mixture was separated using a 140 min gradient form 0% to 100% Buffer B (Buffer A: 2.5% acetonitrile 0.1% formic acid and buffer B 90% acetonitrile, 0.025% trifluoracetic acid, and 0.1% formic acid).

The mass spectrometer was operated in ‘data-dependent mode’, with a single MS scan (400 to 2,000 m/z) in FT mode 60 K resolution followed by 5 MS/MS scans in the linear ion trap on the most abundant ions and dynamic excluded for 120 seconds.

### Protein Identification and Quantification

Conversions from RAW to MGF files were performed as described previously [32], [33]. MS/MS data were searched using MASCOT Versions 2.3 (Matrix Science Ltd., London, UK) against a mouse plus contaminant database downloaded from NCBI with 30,061 sequences (From 12 January 2011) and amended with human APP with the Swedish, Dutch and Iowa mutations due to the transgenic nature of the mouse model. Variable methionine oxidation, STY phosphorylation, protein N-terminal acetylation, and fixed cysteine carbamidomethylation were used in all searches. Precursor mass tolerance was set to 7 ppm and MS/MS tolerance to 0.4 amu. LC-MS label-free protein abundance data derived from MS-speak data were calculated using Progenesis software.

Regarding the label free quantification, the total number of Features (i.e. intensity signal at a given retention time and m/z) was reduced to MS/MS peaks with charge of 2, 3, or 4+ and we only kept the five most intense MS/MS spectra per “Feature”. The subset of multi-charged ions (2+, 3+ and 4+) was extracted from each LC-MS run and the ions intensities summed for normalization. Protein quantification was performed as follows; for a specific protein, the associated unique peptide ions were summed to generate an abundance value. The final list of measured protein abundances were transformed using an ArcSinH function (as the method of detection can generate a significant amount of near zero measurement for which a log transform is not ideal). The within group means were calculated to determine the fold change and the transformed data was then used to calculate the p-values. Differentially expressed proteins were considered most meaningful under the following conditions: Only proteins detected by two or more peptides and p<0.01 associated with the protein change.

### Gene Ontology Analysis

Proteins found to be significantly different at p<0.01 within the WT and Tg-SwDI cohorts were uploaded to WebGestalt Gene Set Analysis Toolkit version 2 (http://bioinfo.vanderbilt.edu/webgestalt, Vanderbilt University) for enrichment analysis based on gene ontology [34], [35]. Cut-off criteria included a p<0.01 adjusted with hypergeometric statistical analysis using Benjamini & Hochberg multiple test adjustment with a minimum molecule cut-off of 2.

### KEGG Pathway Analysis

For pathway analysis, the p-value threshold was set at p<0.05 to strengthen network analysis. Proteins found to be significantly different at p<0.05 within the WT and Tg-SwDI cohorts were uploaded to WebGestalt Gene Set Analysis Toolkit version 2 for enrichment analysis based on the Kyoto Encyclopedia of Genes and Genomes (KEGG) pathway maps using the same criteria above.

### Immunohistochemistry

At 3 and 9 months of age, Tg-SwDI and WT mice were deeply anaesthetised with 5% isoflurane and transcardially perfused with 20 ml 0.9% heparinized phosphate buffered saline followed by 20 ml of 4% paraformaldehyde in 0.1% phosphate buffer. Brains were removed and post-fixed in 4% PFA over-night and paraffin embedded.

Coronal tissue sections from each respective cohort were deparaffinized using standard procedures. Briefly, sections were incubated at 60°C for 30 minutes, followed by further dewaxing in xylene. Slides were then placed sequentially in 100%, 90% and finally 70% alcohol before being rinsed in running water. Sections were processed for antigen retrieval by being incubated in 10 mM citric acid for 10 minutes at 100°C in a pressurized retrieval machine. For APOE immunostaining sections were blocked with normal donkey serum and incubated sequentially with goat polyclonal APOE (Calbiochem) along with mouse monoclonal β-dystroglycan (Leica Microsystems) followed by Alexa Flour488®-labelled mouse monoclonal 6E10 (Covance) overnight at 4°C. APOE and β-dystroglycan were visualized with Cy3-labelled and Cy5-labelled secondary antibodies respectively. For HTRA1 immunostaining sections were blocked with normal goat serum and incubated sequentially with rabbit polyclonal HTRA1 (Abcam) along with mouse monoclonal β-dystroglycan (Leica Microsystems), followed by Alexa Flour488®-labelled mouse monoclonal 6E10 (Covance) overnight at 4°C. HTRA1 and β-dystroglycan were visualized with Alexa Flour 546 and 646 respectively. Sections were analyzed using a laser scanning confocal microscope (Leica SP5). All images were acquired with a 40× oil-immersion objective within the thalamus. Image analysis was carried out using NIH image J 1.47d software.

### Statistical Analysis

Data were analysed using student’s t-test or one-way ANOVA where experimental design exceeded 2 comparisons. For the LC-MS data significance was set at p<0.01. For all other analyses significance was set at p<0.05.

## Results

### Vessel Enrichment: S1 Fractions are Enriched in Vessel-associated Proteins

**Figure 1:**
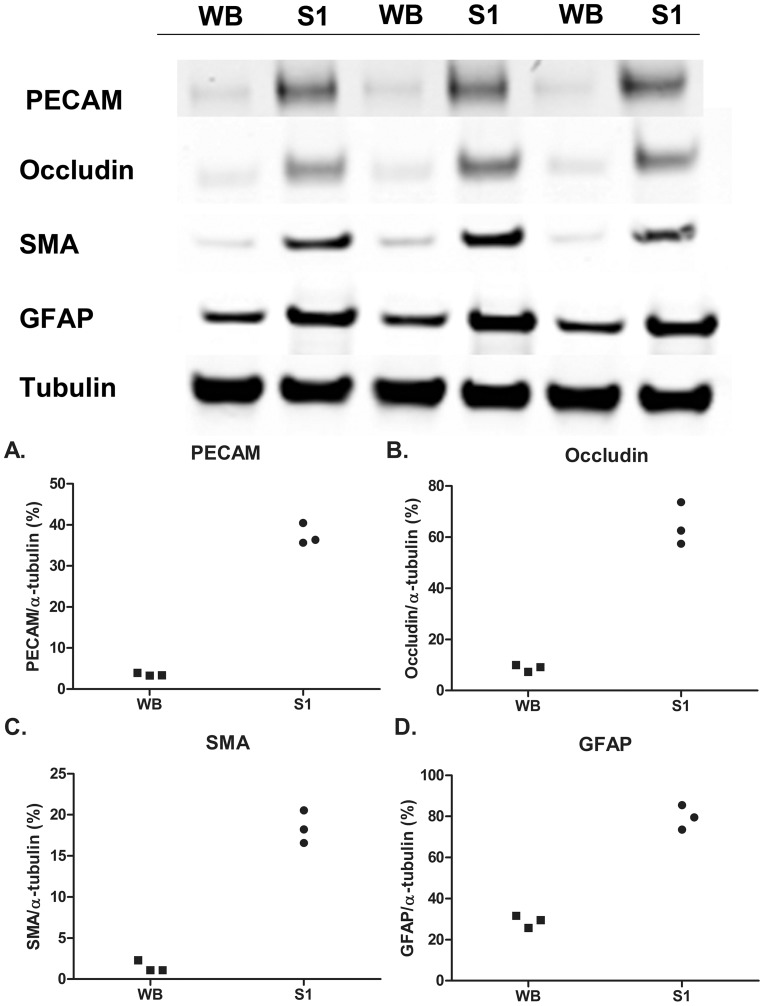
Vessel fractions are enriched in vascular-associated proteins. S1 fractions were enriched in proteins associated with various cellular compartments of cerebral vessels as demonstrated by increased levels of Occludin, PECAM, SMA and GFAP. α-tubulin showed similar expression across all blots. (A–D) Levels of selected proteins (relative to respective α-tubulin levels) were all significantly enriched in the S1 fraction compared to whole brain samples (n = 3/gp; p<0.001; t-test). WB = whole brain fraction; S1 = vessel-enriched fraction.

Vascular enrichment was conducted using modifications of previously published protocols [30], [31]. To verify that there was vascular enrichment, S1 fractions from 3 month-old WT mice (n = 3) were compared to whole brain (WB) fractions and the levels of four cerebrovascular-associated proteins were measured. These proteins were selected due to their critical importance in multiple cellular compartments of cerebral vessels; a marker of vascular smooth muscle (SMA); a marker of endothelial tight junctions (occludin), an endothelial marker (PECAM) and a marker of astrocytes (GFAP). The data demonstrated that S1 fractions were enriched by 3–12 fold [Occludin (7 fold), PECAM (11 fold), SMA (12 fold), GFAP (3 fold)] (Figure 1).

### Temporal Increase in Vascular Aβ1-40

The levels of Aβ1-40 were measured since this is the most prominent Aβ species in the cerebrovasculature of Tg-SwDI animals [23], whose negative charge in its mutant form contributes to its increased pathogenicity [36], [37] and its reduced clearance due to its lower affinity to LRP [38]. As predicted, the mean levels of Aβ1-40 (pg/mg of total tissue ± S.E.M) were significantly higher in vessel fractions from the 12 and 17 month-old Tg-SwDI mice (220.8±6.29 and 226±19.22 respectively) compared to the 3 month-old mice (10.72±10.72; p<0.0001; one-way ANOVA).

### Proteomic Analysis of Vessel Fractions

**Figure 2:**
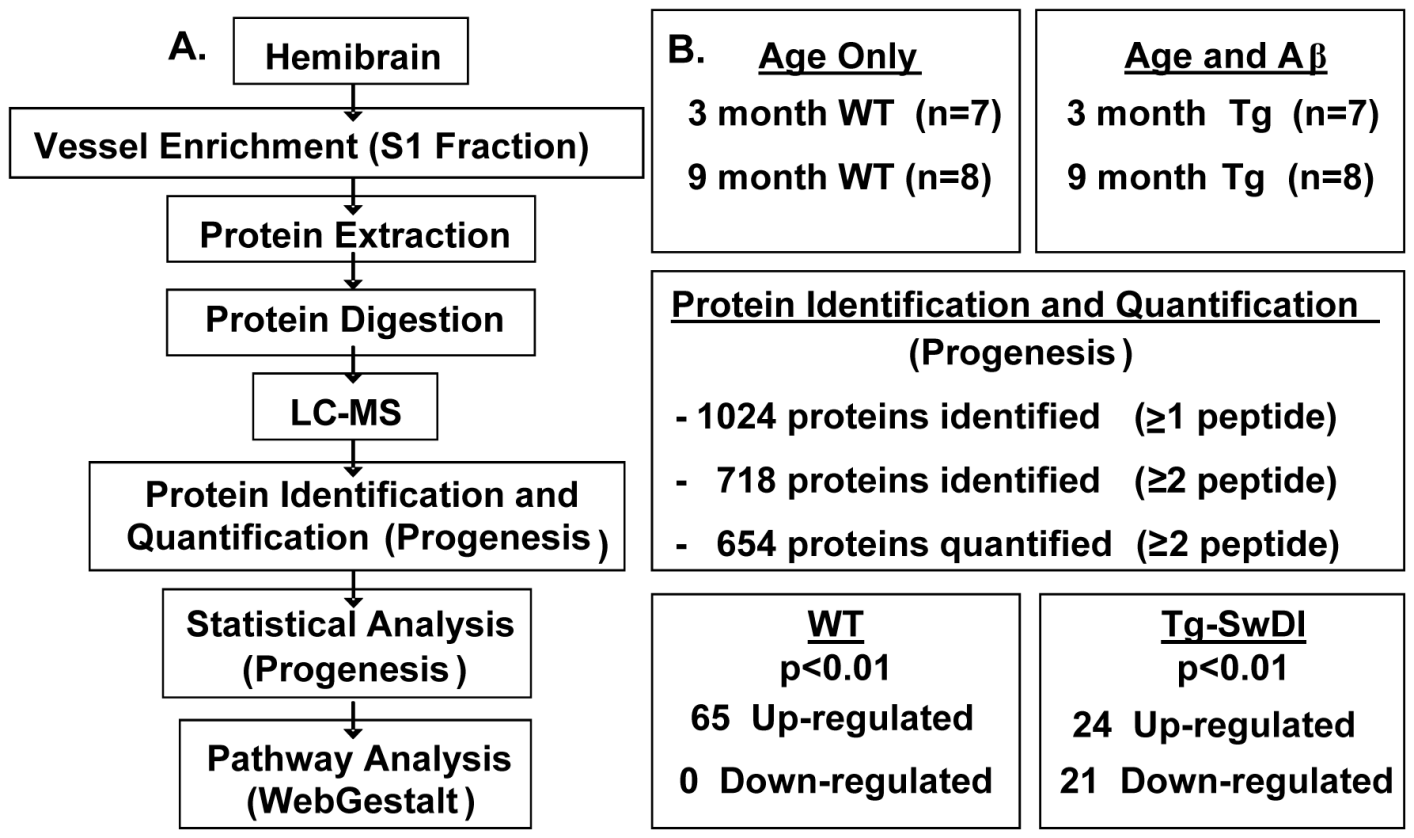
Proteomic design and workflow. (A) Vessel enriched fractions were collected from 3 month-old and 9 month-old WT and 3 month-old and 9 month-old Tg-SwDI mice for proteomic analysis. (B) Over 1,000 proteins were identified, with over 70% being identified by more than two peptides. 654 proteins were quantified by at least two peptides and were statistically analysed using Progenesis software. 65 proteins were found to be up-regulated in the WT animals. 45 proteins were found to be up- and down-regulated (21 and 24 respectively) in the Tg-SwDI animals. Those proteins that were found to be significantly different within the respective cohorts were analysed further with WebGestalt web-based software.

1024 proteins were identified within the S1 fractions from young and older WT and Tg-SwDI mice. 654 were quantified by at least 2 peptides across all groups (a list of all proteins quantified by 2 peptides can be found in Table S1). The proteomic workflow along with a summary of results is presented (Figure 2).

65 proteins were significantly different (p<0.01) between the 3 month-old WT and 9 month-old WT mice. All of these proteins were up-regulated (Table 1).

**Table 1:** Proteins significantly increased in older wild-type mice (9 months) relative to young wild-type mice (3 months).

Description - 9 month Wt vs 3 month WT	Accession	p-value	Fold Change
basement membrane-specific heparan sulfate proteoglycan core protein	gi_183979966	0.000007	2.68
myelin-associated oligodendrocyte basic protein isoform b	gi_86355503	0.00001	2.35
collagen alpha-2(IV) chain preproprotein	gi_226437587	0.00003	2.83
tubulointerstitial nephritis antigen-like precursor	gi_270132817	0.00004	2.88
laminin subunit gamma-1 precursor	gi_153791270	0.0001	2.55
laminin subunit beta-2 precursor	gi_31982223	0.0002	2.42
glycerol-3-phosphate dehydrogenase 1-like protein	gi_257467604	0.0003	1.82
histone H1.4	gi_13430890	0.0003	2.31
nidogen-1 precursor	gi_171543883	0.0003	2.12
agrin	gi_42490751	0.0003	2.00
myelin-oligodendrocyte glycoprotein	gi_113199771	0.0004	1.90
histone H2A type 2-A	gi_20799907	0.0006	1.92
claudin-11	gi_6679186	0.001	1.74
synaptic vesicle membrane protein VAT-1 homolog	gi_33859662	0.001	1.59
myelin basic protein isoform 1	gi_69885032	0.001	1.48
moesin	gi_70778915	0.001	1.61
fibrinogen gamma chain precursor	gi_19527078	0.001	8.77
3-ketoacyl-CoA thiolase, mitochondrial	gi_29126205	0.001	1.51
receptor expression-enhancing protein 5	gi_161016826	0.001	1.48
vimentin	gi_31982755	0.001	1.66
myelin proteolipid protein	gi_23956058	0.002	1.83
cysteine and glycine-rich protein 1	gi_6681069	0.002	1.56
solute carrier family 2, facilitated glucose transporter member 1	gi_165377226	0.002	1.51
nidogen-2 precursor	gi_84370361	0.002	2.23
D-3-phosphoglycerate dehydrogenase	gi_52353955	0.002	1.55
coronin-2B	gi_148528987	0.002	1.81
peroxiredoxin-6	gi_6671549	0.003	1.33
glycerol-3-phosphate dehydrogenase, mitochondrial precursor	gi_224922803	0.003	1.57
peptidyl-prolyl cis-trans isomerase FKBP1A	gi_6679803	0.003	1.63
voltage-dependent anion-selective channel protein 3 isoform 2	gi_6755967	0.003	1.54
microtubule-associated protein 1A isoform 1	gi_124244033	0.003	1.61
voltage-dependent anion-selective channel protein 2	gi_6755965	0.003	1.61
tubulin beta-2C chain	gi_22165384	0.003	1.63
myosin-11 isoform 1	gi_241982716	0.003	2.12
CD9 antigen	gi_6680894	0.003	2.18
succinyl-CoA ligase [GDP-forming] subunit alpha, mitochondrial precursor	gi_255958286	0.003	1.94
multidrug resistance protein 3	gi_153791547	0.003	1.48
acyl-CoA-binding protein isoform 2	gi_6681137	0.004	1.72
mesotrypsin	gi_6755891	0.004	1.80
alpha-internexin	gi_148539957	0.004	1.49
contactin-associated protein 1 precursor	gi_116063560	0.004	1.52
dihydropteridine reductase	gi_21312520	0.004	1.56
histone H1.0	gi_31560697	0.004	1.83
aldehyde dehydrogenase, mitochondrial precursor	gi_6753036	0.004	1.56
histone H1.2	gi_9845257	0.005	1.78
band 3 anion transport protein	gi_6755560	0.005	2.44
profilin-2	gi_9506971	0.005	1.51
peroxiredoxin-2	gi_148747558	0.006	1.45
guanine nucleotide-binding protein G(i) subunit alpha-2	gi_41054806	0.006	1.41
electron transfer flavoprotein subunit beta	gi_38142460	0.006	1.48
myelin-associated glycoprotein	gi_6754614	0.006	1.50
carbonic anhydrase 2	gi_157951596	0.006	1.74
choline transporter-like protein 1 isoform A	gi_227499980	0.007	1.83
60S ribosomal protein L13	gi_33186863	0.007	2.12
hemoglobin subunit beta-1	gi_31982300	0.007	3.97
potassium voltage-gated channel subfamily A member 2	gi_157012015	0.008	1.48
cytochrome c, somatic	gi_6681095	0.009	2.16
cytochrome b-c1 complex subunit 8	gi_21539585	0.009	1.41
microtubule-associated protein 6 isoform 1	gi_113204613	0.009	1.54
prohibitin-2	gi_126723336	0.009	1.38
plectin isoform 1d	gi_254675251	0.009	1.43
hemoglobin alpha, adult chain 2	gi_145301549	0.009	4.09
tubulin beta-5 chain	gi_7106439	0.010	1.53
MOSC domain-containing protein 2, mitochondrial precursor	gi_19526848	0.010	1.52
tubulin beta-4 chain	gi_31981939	0.010	1.56

45 proteins were found to be significantly different (p<0.01) between the 3 month-old Tg-SwDI and 9 month-old Tg-SwDI mice. 24 proteins were up-regulated and 21 were down-regulated (Table 2).

**Table 2:** Proteins significantly increased (positive fold change) and decreased (negative fold change) in older Tg-SwDI mice (9 months) relative to young Tg-SwDI mice (3 months).

Description - 9 month Tg-SwDI vs 3 month Tg-SwDI	Accession	p-value	Fold Change
apolipoprotein E precursor	gi_163644329	0.000003	3.86
serine protease HTRA1 precursor	gi_229093139	0.000003	6.26
heterogeneous nuclear ribonucleoproteins A2/B1 isoform 2	gi_32880197	0.00003	−3.96
heterogeneous nuclear ribonucleoprotein A1 isoform a	gi_6754220	0.0001	−2.96
heterogeneous nuclear ribonucleoprotein U-like protein 2	gi_124487099	0.0001	−3.82
heterogeneous nuclear ribonucleoprotein D0 isoform a	gi_116256512	0.0002	−3.49
heterogeneous nuclear ribonucleoprotein K	gi_13384620	0.0003	−3.09
heterogeneous nuclear ribonucleoproteins C1/C2 isoform 1	gi_8393544	0.0003	−2.95
heterogeneous nuclear ribonucleoprotein L	gi_183980004	0.0003	−2.87
CD9 antigen	gi_6680894	0.001	2.44
myelin-associated oligodendrocyte basic protein isoform b	gi_86355503	0.001	2.24
alpha-crystallin B chain	gi_6753530	0.001	2.68
protein kinase C & casein kinase substrate in neurons protein 1 isoform 1	gi_6754974	0.001	2.23
heterogeneous nuclear ribonucleoprotein H	gi_10946928	0.001	−2.84
CUGBP Elav-like family member 2 isoform 6	gi_124286791	0.001	−3.25
alcohol dehydrogenase [NADP+]	gi_10946870	0.001	1.84
TAR DNA-binding protein 43 isoform 1	gi_21704096	0.001	−2.44
myelin expression factor 2 isoform 1	gi_244790087	0.001	−2.57
gap junction alpha-1 protein	gi_6753992	0.002	2.19
V-type proton ATPase subunit G 2	gi_12963559	0.002	2.06
serine/arginine-rich splicing factor 7 isoform 1	gi_22122585	0.002	−1.67
lupus La protein homolog	gi_6678143	0.002	−2.32
intercellular adhesion molecule 5 precursor	gi_159110562	0.002	2.47
poly(rC)-binding protein 2 isoform 1	gi_157041229	0.003	−3.05
matrin-3	gi_25141233	0.003	−2.08
heterogeneous nuclear ribonucleoprotein U	gi_160333923	0.003	−2.13
profilin-1	gi_6755040	0.004	2.20
polypyrimidine tract-binding protein 2	gi_9507003	0.004	−3.13
mitochondrial glutamate carrier 2	gi_124486670	0.004	1.96
protein bassoon	gi_124487407	0.005	1.90
glial fibrillary acidic protein isoform 2	gi_84000448	0.005	1.85
clathrin light chain A isoform a	gi_122939192	0.005	1.66
D-3-phosphoglycerate dehydrogenase	gi_52353955	0.005	1.74
heterogeneous nuclear ribonucleoprotein H2	gi_9845253	0.006	−2.51
peroxiredoxin-6	gi_6671549	0.006	1.42
vesicle-associated membrane protein-associated protein A	gi_94721328	0.007	1.71
vesicle-associated membrane protein-associated protein B	gi_31543940	0.007	2.04
KH domain-contain., RNA-binding, signal transduction-assoc. protein 1	gi_110626031	0.007	−3.80
proline-rich transmembrane protein 2	gi_156523248	0.008	1.74
heterogeneous nuclear ribonucleoprotein G	gi_83699420	0.008	−1.80
heterogeneous nuclear ribonucleoprotein R	gi_33859724	0.008	−2.22
3-ketoacyl-CoA thiolase, mitochondrial	gi_29126205	0.009	1.74
heterogeneous nuclear ribonucleoprotein A/B isoform 2	gi_6754222	0.009	−2.32
PREDICTED: ras GTPase-activating protein SynGAP	gi_309263645	0.009	1.80
myc box-dependent-interacting protein 1 isoform 1	gi_6753050	0.010	1.57
potassium voltage-gated channel subfamily A member 2	gi_157012015	0.010	1.99

Of those proteins that were significantly different within the cohorts, only 6 proteins overlapped between the WT and Tg-SwDI cohorts. This indicates differential responses to age within the WT and Tg-SwDI cohorts (Tables 1&2).

### KEGG Pathway Analysis

Proteins found to be significantly different (p<0.05) between the 3 month-old and 9 month-old WT animals (178 proteins) along with those significantly different (p<0.05) between the 3 month-old Tg-SwDI and 9 month-old Tg-SwDI animals (154 proteins) were uploaded to the WebGestalt Gene Set Analysis Toolkit version 2 for cohort-specific KEGG pathway analysis.

**Figure 3:**
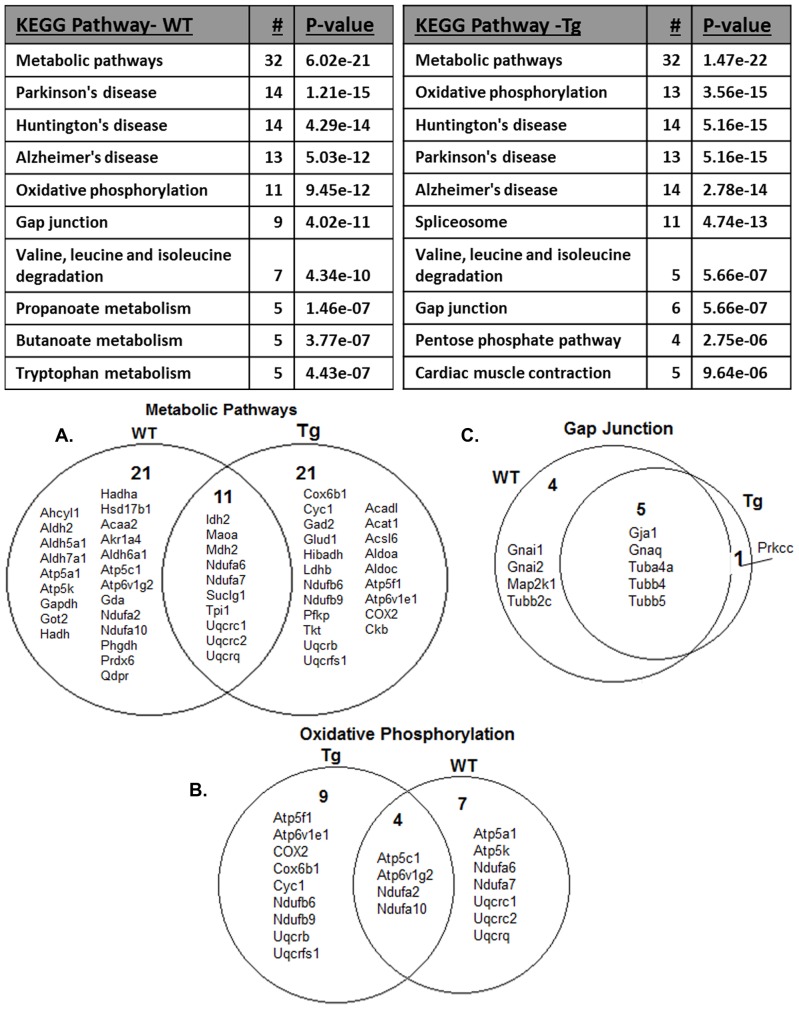
KEGG pathway analysis suggests similar patterns of expression. The Kyoto Encyclopedia of Gene and Genomes (KEGG) pathway analysis resulted in similar lists of modulated pathways representing proteins found to be significantly within the different cohorts (p<0.05; t-test). Venn diagrams show divergences in the proteins that make up those pathways. Both WT and Tg-SwDI animals show changes in (A) *metabolic pathways*, specifically (B) *oxidative phosphorylation*, though only a third of the modulated proteins represented by the KEGG pathways are shared. The (C) gap junction pathway share many more proteins between WT and Tg-SwDI cohorts.

KEGG pathway analysis revealed similar pathways being represented within the WT and Tg-SwDI cohorts. The top biological processes for both cohorts included: *metabolic pathways* and several neurodegenerative related pathways: *Alzheimer’s disease, Parkinson’s disease* and *Huntington disease* (See figure 3). Both 9 month-old WT and 9 month-old Tg-SwDI animals showed up-regulation of proteins represented by the *oxidative phosphorylation* category as well as the *gap junction* category. Closer inspection of the proteins contained within certain categories reveal a divergence in the proteins being modulated within the respective cohorts, with only about a third of the proteins being shared within *the metabolic pathways* and *oxidative phosphorylation* categories. Other categories (e.g. *gap junction*) show much more convergence.

### Age Effects on the WT Vascular Proteome

**Figure 4:**
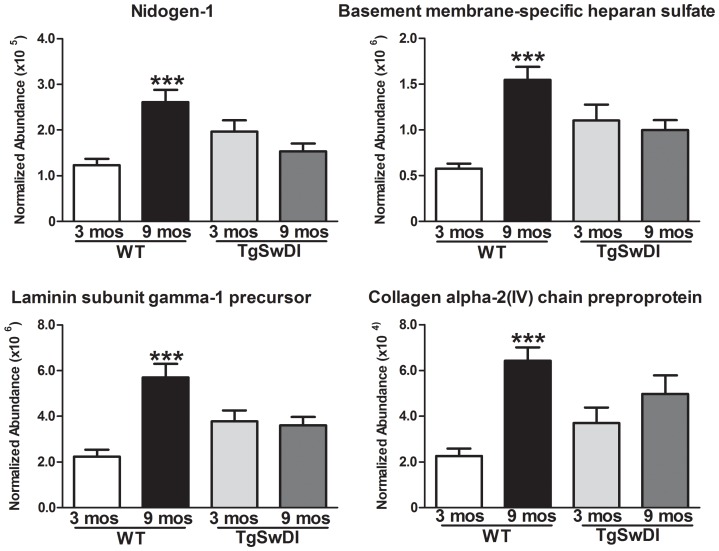
Age effects on the WT vascular proteome. 4 selected basement membrane proteins were significantly up-regulated in 9 month-old WT animals by at least 2 fold (Mean abundance ± S.E.M.; ***p<0.001; t-test). No significant difference was observed in the Tg-SwDI animals for these same proteins.

In WT animals, age led to a marked up-regulation of proteins associated with the basement membrane based on gene ontology (cellular compartment; Figure S1), particularly nidogen-1, basement membrane-specific heparan sulfate proteoglycan core protein, laminin subunit gamma-1 precursor and collagen alpha-2(IV) chain preproprotein (Figure 4).

Age also led to the up-regulation of several white matter- associated proteins in WT animals (and to a lesser extent in Tg-SwDI animals, including myelin-associated oligodendrocyte basic protein, myelin-oligodendrocyte glycoprotein, myelin proteolipid protein, myelin associated glycoprotein, contactin-associated protein-1 precursor and myelin basic protein isoform 1 (Figure S2).

### Age Effects on the Tg-SwDI Vascular Proteome

**Figure 5:**
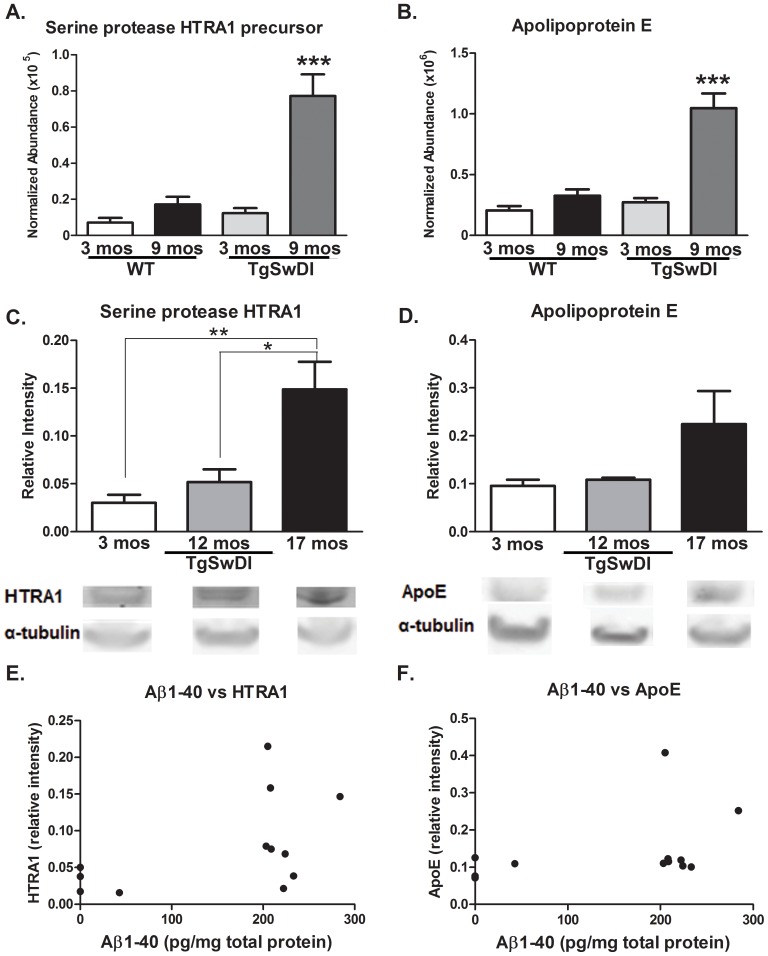
Age effects on the Tg-SwDI vascular proteome. (A) Serine protease HTRA1 and (B) Apolipoprotein E were significantly different between 3 month-old and 9 month-old Tg-SwDI animals (Mean abundance ± S.E.M; ***p<0.0001; t-test), whereas no differences were detected between the WT animals of the same age. Serine protease HTRA1 was the most up-regulated protein within the Tg cohort (fold change = 6.26) followed by APOE (fold change = 3. 86). In separate cohorts to those used for LC-MS, immunoblot analysis of 3, 12 and 17 month-old Tg-SwDI animals (n = 4/grp, representative blots shown below respective cohort) confirmed an age-dependent increase in (C) HTRA1 (Mean relative intensity ± S.E.M; *p<0.05; **p<0.01, one-way ANOVA, Bonferroni post-test) and in (D) APOE levels (Mean relative intensity ± S.E.M; p = 0.097, one-way ANOVA). No correlation could be made between Aβ1-40 levels and (E) HTRA1 or (F) APOE levels in the same vessel fractions.

HTRA1 and APOE were markedly increased in the 9 month-old as compared to 3 month-old Tg-SwDI animals (*p<0.0001) (Figure 5A, B). There were no differences detected between 3 month and 9 month-old WT mice. Serine protease HTRA1 was the most up-regulated protein within the Tg cohort (fold change = 6.26) followed by APOE (fold change = 3. 86). To confirm that these alterations in APOE and HTRA1 were a robust finding and increasing with age, the levels of these proteins were measured by Western blot in separate cohorts to those in which LC-MS was conducted at ages 3, 12 and 17 months (Tg-SwDI n = 4/grp). Immunoblot analysis confirmed a age-dependent increase in HTRA1 (*p<0.05; **p<0.01) (Figure 5C) and in APOE levels (p = 0.096) in vascular fractions (Figure 5D). However, there was no correlation between Aβ1-40 levels and HTRA1 or APOE levels in the same vessel fractions (Figure 5E, F).

**Figure 6:**
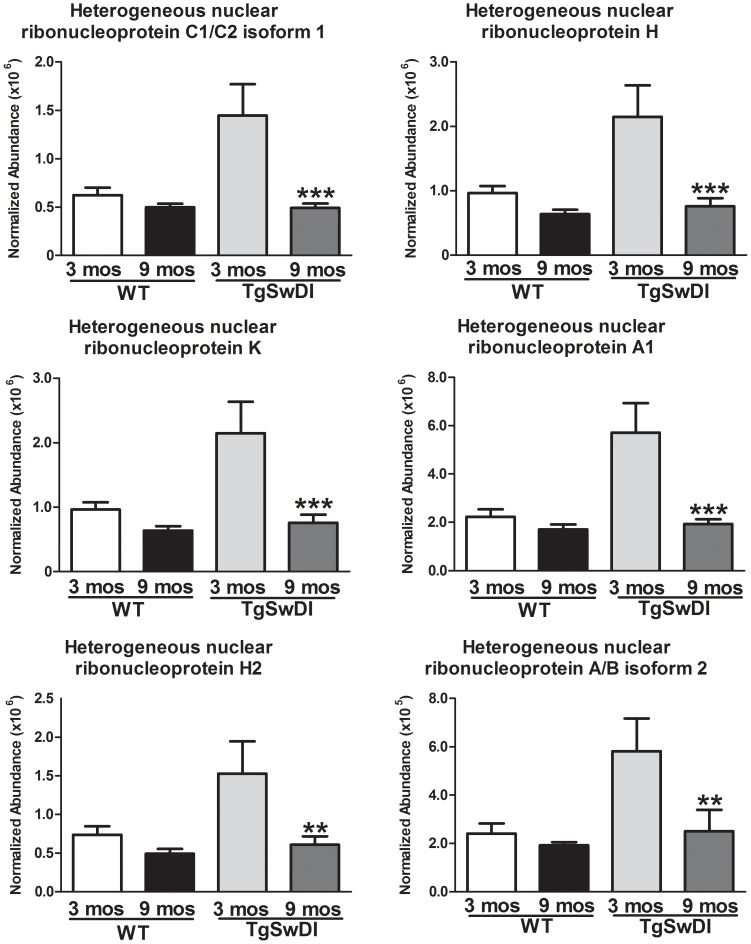
Age effects on the Tg-SwDI vascular proteome. Multiple heterogeneous nuclear ribonucleoproteins were down-regulated in 9 month-old Tg-SwDI animals (Mean abundance ± S.E.M; ***p<.001; **p<0.01; t-test) compared to younger animals. No significant difference was observed in the WT cohort.

Age led to the extensive down-regulation of several proteins associated with the *spliceosome* (note figure 3) and RNA processing in the Tg-SwDI animals based on gene ontology (biological process; Figure S3). These include several members of the heterogeneous nuclear ribonucleoprotein family of proteins (Figure 6).

### APOE Localizes to Cerebral Vessels and Accumulated Aβ

**Figure 7:**
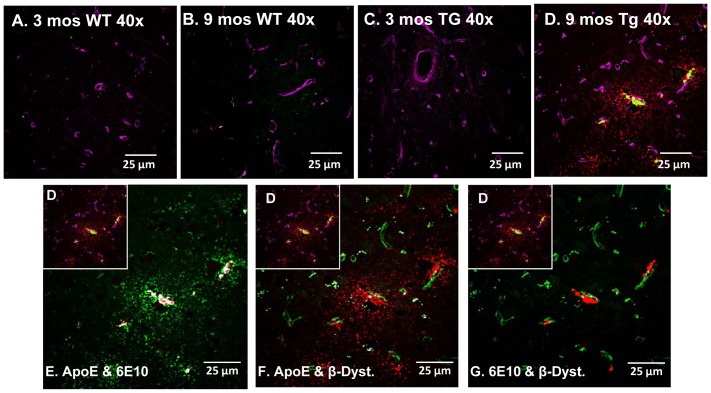
Increases in APOE levels are specific to 9 mos Tg-SwDI mice. Tissue sections representative of independent 3 and 9 month-old WT (A&B) and 3 and 9 month-old Tg-SwDI (C&D) animals were triple-labelled with APOE (red), 6E10 (green) and β-dystroglycan (magenta) and analysed with a laser scanning confocal microscope within the thalamus (400×). Immunohistochemical analysis of the four different cohorts show APOE and Aβ present only in the (D) 9 month Tg-SwDI animals. APOE co-localized (shown in white) with both (E) 6E10-labelled Aβ and (F) β-dystroglycan-labelled cerebral vessels. (G) Aβ is closely associated with cerebral vessels.

Tissue from independent cohorts of 3 month-old WT, 9 month-old WT, 3 month-old Tg-SwDI and 9 month-old Tg-SwDI mice were immunolabelled with antibodies to β-dystroglycan, Aβ and APOE. APOE immunolabelling was mostly observed in 9 month-old Tg-SwDI animals (Figure 7A–D). APOE co-localized to both accumulated Aβ and cerebral vessels, particularly those vessels proximate to Aβ deposits within the thalamus.

### HTRA1 is Located Proximately to Aβ-laden Vessels in the Thalamus

**Figure 8:**
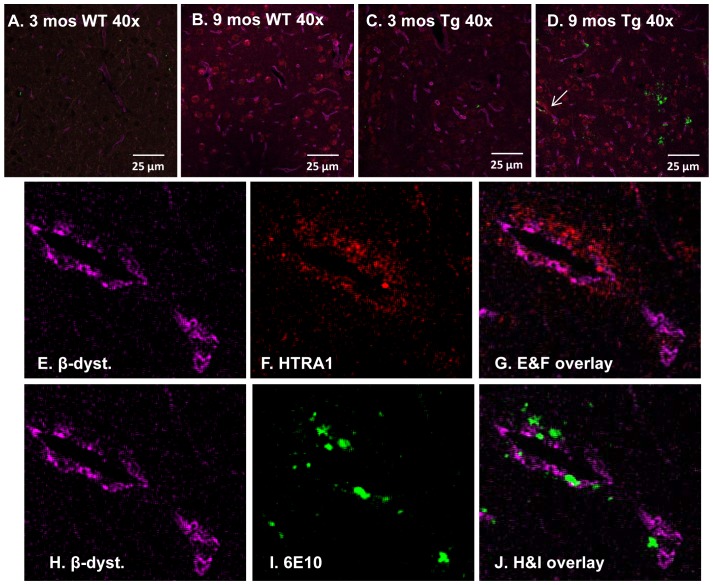
HTRA is located proximately to Aβ-laden vessels in the thalamus of 9 mos Tg-SwDI mice. Tissue sections representative of independent 3 and 9 month-old WT (A&B) and 3 and 9 month-old Tg-SwDI (C&D) animals were triple-labelled with HTRA1 (red), 6E10 (green) and β-dystroglycan (magenta) and analysed with a laser scanning confocal microscope (400×). Immunohistochemical analysis of the four different cohorts show HTRA1 closely associated with Aβ-laden vessels [white arrow, (D)] within the thalamus of 9 month-old Tg-SwDI mice (E–J) and appeared extracellular.

Tissue from independent cohorts of 3 month-old WT, 9 month-old WT, 3 month-old Tg-SwDI and 9 month-old Tg-SwDI mice (as above) were immunolabelled with antibodies to β-dystroglycan, Aβ and HTRA1. HTRA1 immunolabelling was mostly observed in 9 month-old WT and Tg-SwDI sections and appeared to be expressed cytoplasmically (Figure8 A–D). Only in 9 month-old Tg-SwDI mice was HTRA1 found to be located proximately to Aβ-laden vessels (Figure 8E–J) within the thalamus and appeared extracellular.

## Discussion

Brain ageing leads to alterations in the protein components of the cerebrovascular milieu, which can lead to changes in vascular integrity and decreased functioning of the NVU and the BBB [6], [7], [8]. Pathological insults such as CAA can accelerate these changes, [39] [40], [41]. Quantitative proteomics allowed an in-depth and global investigation of the protein profiles of the cerebrovascular milieu of WT and Tg-SwDI animals at distinctly different ages. We were able to identify proteins that might be contributing to these age-dependent changes by looking specifically at vascular enriched fractions. These vascular fractions were enriched in proteins associated with cerebral vessels (PECAM, occludin, SMA, GFAP, ABCB1A, nidogen, claudin 11 and AQP4, Table S1) using techniques similar to those used under physiological conditions [31] and in genetically altered models with cerebral haemorrhage propensity [42].

The number of proteins that were changed between the 3 and 9 month-old WT animals (n = 65) and the 3 and 9 month-old Tg-SwDI animals (n = 45) were substantial in number, yet the protein lists generated from the two cohorts had very little overlap, suggesting that age affected the vascular proteomic profile of the WT and Tg-SwDI animals in very different ways. We hypothesized that age would have a differential effect on the cerebrovascular proteome in the presence of microvascular Aβ.

KEGG pathway analysis suggested that many of the pathways enriched by significantly modulated proteins were in fact very similar between the WT and Tg-SwDI cohorts. Further scrutiny of these revealed minimal overlap of the proteins the categorical terms represented, again confirming differential regulation of protein expression within the two cohorts. Examples of this include two of the most significantly enriched pathways for both WT and Tg-SwDI cohorts: *metabolic pathways* and *oxidative phosphorylation* indicating changes in multiple mitochondrial proteins. Only 30% of those mitochondrial proteins that were found to be differentially modulated with the cohorts overlapped.

Despite the lack of overlap, modulations of mitochondrial proteins are of great importance to age-related vascular changes. The cerebrovasculature endothelium is highly enriched in mitochondria due to the high energy demands required for neurovascular coupling [43] as well as for the multitude of ATP-dependent transporters at the BBB required for bidirectional transport of molecules to the brain [6]. Mitochondrial dysfunction is associated with both brain ageing and accumulating Aβ and is closely linked to increases in oxidative stress [44], [45], [46], [47]. Such modulations like those observed in the older WT and Tg-SwDI animals suggest that the age does lead to changes in the expression of certain mitochondrial proteins, especially those that make up the electron transport chain.

Basement membrane thickening due to increases in proteins such as collagen has been observed with age and AD [8], [48], [49]. Of the 65 proteins up-regulated in 9 month-old WT animals, 10% are intimately linked to the basement membrane (nidogen-1, nidogen-2, laminin subunit gamma-1 precursor, collagen alpha-2(1V) chain preproprotein, laminin subunit beta-2 precursor, basement membrane specific heparin sulphate proteoglycan core protein, agrin). At 9 months of age the mice are not aged by convention, yet there is still evidence of a temporal increase in key basement membrane proteins over a 6 month period. This increase is not seen in the Tg-SwDI mice. Instead, comparison of these protein levels across all 4 groups indicates the abundance of these same proteins is typically greater in 3 month-old Tg-SwDI animals as compared to 3 month-old WT animals (Figure 4) and remained stable as the animals aged and Aβ accumulated.

In the present study, alterations in protein signatures were studied in vascular-enriched fractions which allows for the identification of proteins in cellular compartments in close association with the vasculature. Notably white matter-associated proteins were identified and found to be altered in older WT and TgSWDI mice as compared to the respective younger cohorts. This included the significant up-regulation of myelin-oligodendrocyte glycoprotein, myelin proteolipid protein, myelin associated glycoprotein, contactin-associated protein-1 precursor and myelin basic protein isoform 1 in older WT mice. Only myelin-associated oligodendrocyte basic protein, the third most prevalent protein in myelin [50] was found to be significantly up-regulated in both older WT and Tg-SwDI mice. Furthermore, in older Tg-SwDI mice, a down-regulation in myelin expression factor 2 isoform 1, a suppressor of MBP expression [51] was observed, which was not seen in the older WT mice. This is an indication that these white matter-associated proteins are differentially altered in older WT mice as compared to those found to be altered in the older TgSWDI mice.

The integrity of white matter has been reported to be compromised in ageing and in models of amyloidosis [52], [53], [54]. Whilst studies have previously shown alterations in myelin proteins with ageing the results are conflicting with some studies showing a reduction in specific proteins like myelin basic protein [55] and others finding the up-regulation or complete lack of change in proteins such as 2′,3′ cyclic nucleotide 3′-phosphodiesterase or proteolipid protein respectively [56]. Furthermore the spatial organisation of proteins on myelinated axons, such as those found within paranodal regions, may be altered and can respond dynamically to various challenges including cerebral hypoperfusion and amyloid plaques [57], [58], [59]. In the present study we found a marked up-regulation of a greater number of myelin-associated proteins in older WT mice compared to TgSWDI. These differences are likely due to whn and how these changes are measured and suggest complex regulatory control of myelin protein expression. One of the most up-regulated proteins within the 9 month-old Tg-SwDI animals was APOE. APOE is known to bind to Aβ and is involved in its deposition and transcystosis across the blood brain barrier via its interaction with LDL-related protein (LRP) [60], [61]. APOE expression has been manipulated in various ways within the Tg-SwDI mouse model; though very little data exists showing how the endogenous mouse APOE acts naturally within the model. Expression of human APOE in the Tg-SwDI mouse leads to a very different pattern of Aβ deposition than does endogenous APOE, such that human APOE reduces microvascular accumulation of Aβ [62]. Furthermore, when APOE is blocked from interacting with Aβ in Tg-SwDI mice, a reduction in vascular fibrillar amyloid is observed [63]. We show that the APOE co-localizes to Aβ deposits closely associated with cerebral vessels. Perivascular drainage of Aβ is thought to play a critical role in the clearance of Aβ [64] and APOE has been shown to co-localize to perivascular spaces as well as with astrocytic endfeet in transgenic mouse models of amyloidosis [65]. Recent work undertaken using specific antibodies to APOE suggests that APOE might compete with clearance mechanisms for Aβ at the BBB, and suggests potential novel targets for therapeutic intervention to reduce amyloidosis [66], [67].

We have also found a significant increase in the HTRA1 within the vascular fractions of the Tg-SwDI mice at 9 months of age. HTRA1 is a member of the trypsin family of serine proteases that has properties similar to many heat shock proteins and typically acts to prevent the aggregation of misfolded proteins [68]. It is known to inhibit Tgfβ signalling [69] and missense mutations are associated with cerebral autosomal recessive arteriopathy with subcortical infarcts and leukoencephalopathy [70]. HTRA1 cleaves multiple extracellular matrix (ECM) proteins including fibronectin, collagen and aggrecan, which in turn could lead to ECM remodelling [71]. HTRA1 has also been shown to degrade proteins aggregates such as tau [72] and has been shown previously to associate with Aβ, and specifically vascular Aβ [73]. Further, when HTRA1 is inhibited in astrocyte cultures that secrete Aβ 1–40, there is a dose-dependent increase in the level of Aβ1-40, the major Aβ species observed in CAA [73]. The presence of HTRA1 in the vessels may indicate a potential alternative clearance method of Aβ or its involvement in ECM remodelling around the blood vessels in reaction to accumulating Aβ.

Tg-SwDI animals also showed a very significant down-regulation of proteins associated with RNA binding, processing and transport [e.g. spliceosome proteins, heterogeneous nuclear ribonucleoproteins (HNRNPs), TAR DNA-binding protein-43 (TDP-43)] at 9 months of age compared to 3 months of age. The spliceosome is required for proper protein synthesis, as it is involved in the formation of functional mRNA through excision of introns and splicing of exons [74], [75]. Heterogeneous nuclear ribonucleoproteins are involved in RNA binding and processing [76], [77]. A large number of RNA-binding proteins have prion-like domains (e.g. HNRNPA1, HNRNPAB, HNRNPA2B1, TDP-43 [78]), some of these, such as TDP-43, have been implicated as major disease proteins in neurodegenerative diseases such as amyotrophic lateral sclerosis and frontotemporal lobar degeneration and as secondary features of diseases like Alzheimer’s disease and Parkinson’s disease [79].

Age has distinctly different effects on the proteomic profiles of cerebral vessels from WT and Tg-SwDI animals. This is likely due to the transgenic nature of the Tg-SwDI animals, which leads to the vascular accumulation of Aβ1-40. These differential effects include changes in proteins from various cellular compartments and with diverse cellular functions. Further investigation of these proteins is warranted and may give greater insight to the mechanisms behind age-related cerebrovascular dysfunction and pathologies as well as provide novel therapeutic targets.

## Supporting Information

Figure S1 **Gene ontology analysis of proteins found to be significantly different at p<0.01 within the WT cohort were uploaded to WebGestalt Gene Set Analysis Toolkit version 2.**
(TIF)

Figure S2 A number of white matter-associated proteins are significantly up-regulated in 9 month-old WT and Tg-SwDI animals (**p<0.01; t-test). See **Table 2** **for full list.**
(TIF)

Figure S3 **Gene ontology analysis of proteins found to be significantly different at p<0.01 within the Tg-SwDI cohort were uploaded to WebGestalt Gene Set Analysis Toolkit version 2.**
(TIF)

Table S1 **Global list of all proteins quantified by at least two peptides.**
(XLS)

Table S2 **Gene ontology analysis of proteins found to be significantly different at p<0.01 within the WT cohort were uploaded to WebGestalt Gene Set Analysis Toolkit version 2.**
(HTML)

Table S3 **Gene ontology analysis of proteins found to be significantly different at p<0.01 within the Tg-SwDI cohort were uploaded to WebGestalt Gene Set Analysis Toolkit version 2.**
(HTML)
