# Supplementary figures and images for: Impact of Age on the Cerebrovascular Proteomes of Wild-Type and Tg-SwDI Mice
Source: PLoS One. 2014 Feb 26;9(2):e89970. doi: 10.1371/journal.pone.0089970 (PMC3935958; doi:10.1371/journal.pone.0089970)

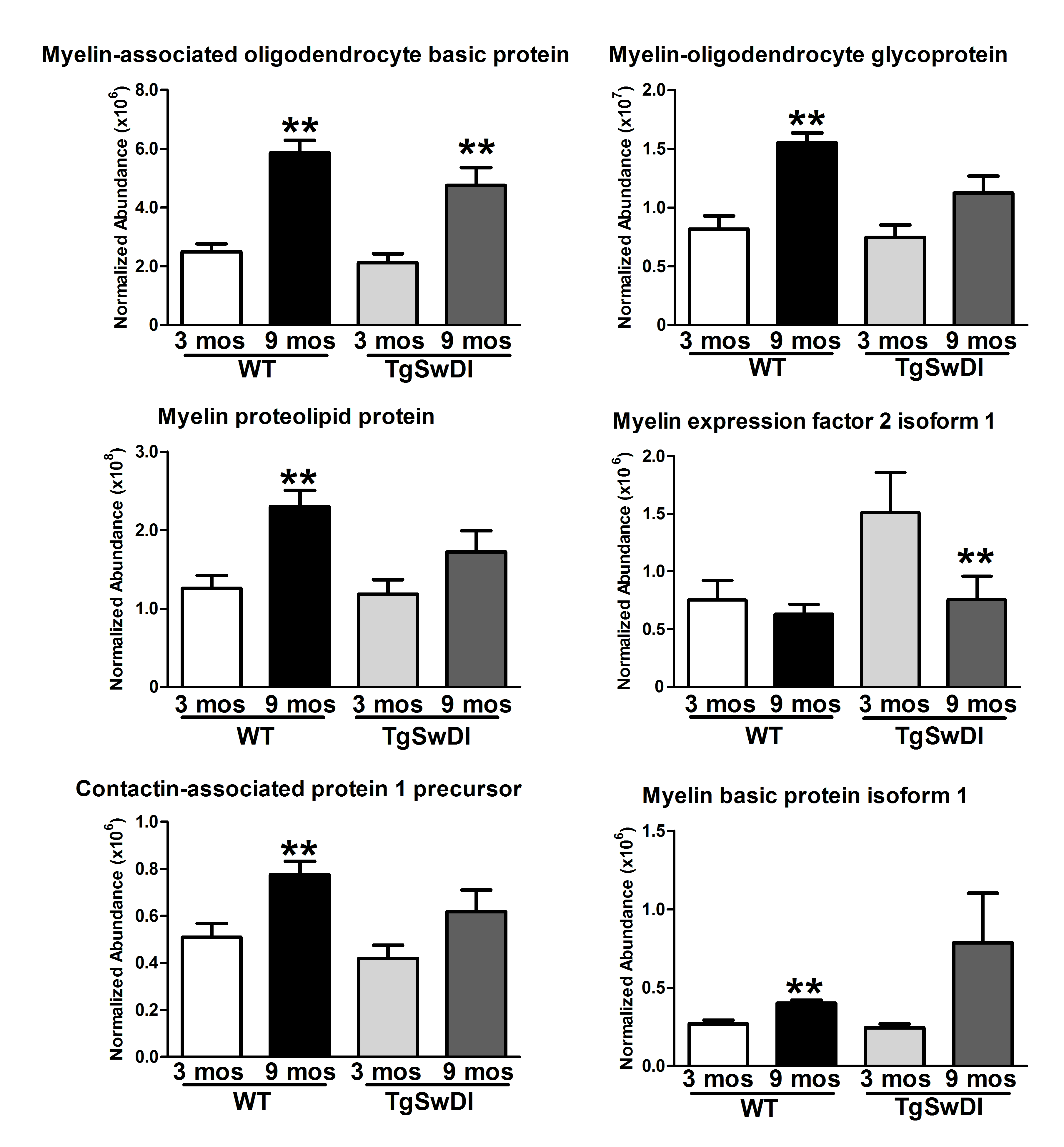

Supplement: Figure S2 — A number of white matter-associated proteins are significantly up-regulated in 9 month-old WT and Tg-SwDI animals (**p<0.01; t-test). See Table 2 for full list. (TIF) [file pone.0089970.s002.tif]
