# Supplementary material for: Impact of Age on the Cerebrovascular Proteomes of Wild-Type and Tg-SwDI Mice
Source: PLoS One. 2014 Feb 26;9(2):e89970. doi: 10.1371/journal.pone.0089970 (PMC3935958; doi:10.1371/journal.pone.0089970)
Supplement: Table S2 — Gene ontology analysis of proteins found to be significantly different at p<0.01 within the WT cohort were uploaded to WebGestalt Gene Set Analysis Toolkit version 2. (HTML) [file pone.0089970.s005.html]

Anchored HTML File of EIDs


|  |  |  |  |  |  |
| --- | --- | --- | --- | --- | --- |
| **User file and parameters:** User file: C57\_3vs9mos\_p01\_webgestalt.txt, Organism: mmusculus, Id Type: uniprot\_swissprot\_accession, Ref Set: entrezgene, Significance Level: Top10, Statistics Test: Hypergeometric, MTC: BH, Minimum: 2  The results for each enriched GO category are listed in this table. For each GO category, the first row lists its sub-root (biological process, molecular function, or cellular component), category name, and corresponding GO ID. The second row lists number of reference genes in the category (C), number of genes in the gene set and also in the category (O), expected number in the category (E), Ratio of enrichment (R), p value from hypergeometric test (rawP), and p value adjusted by the multiple test adjustment (adjP). Finally, genes in the category are listed. For each gene, the table lists the user uploaded ID and value (optional), Entrez ID, Ensembl Gene Stable ID, Gene symbol, and description. Ensembl Gene Stable ID and Entrez Gene ID are linked to the Ensembl and Entrez Gene databases, respectively. | | | | | |
| **biological process----oxidation reduction----GO:0055114** | | | | | |
| C=634;O=12;E=2.28;R=5.27;rawP=1.82e-06;adjP=0.0004 | | | | | |
| Q544B1 | NA | 11669 | ENSMUSG00000029455 | Aldh2 | aldehyde dehydrogenase 2, mitochondrial |
| P47738 | NA | 11669 | ENSMUSG00000029455 | Aldh2 | aldehyde dehydrogenase 2, mitochondrial |
| Q64521 | NA | 14571 | ENSMUSG00000026827 | Gpd2 | glycerol phosphate dehydrogenase 2, mitochondrial |
| Q9CQ69 | NA | 22272 | ENSMUSG00000044894 | Uqcrq | ubiquinol-cytochrome c reductase, complex III subunit VII |
| Q8BVI4 | NA | 110391 | ENSMUSG00000015806 | Qdpr | quinoid dihydropteridine reductase |
| Q6GT24 | NA | 11758 | ENSMUSG00000026701 | Prdx6 | peroxiredoxin 6 |
| O08709 | NA | 11758 | ENSMUSG00000026701 | Prdx6 | peroxiredoxin 6 |
| Q56A15 | NA | 13063 | ENSMUSG00000063694 | Cycs | cytochrome c, somatic |
| P62897 | NA | 13063 | ENSMUSG00000063694 | Cycs | cytochrome c, somatic |
| Q499X4 | NA | 26949 | ENSMUSG00000034993 | Vat1 | vesicle amine transport protein 1 homolog (T californica) |
| Q62465 | NA | 26949 | ENSMUSG00000034993 | Vat1 | vesicle amine transport protein 1 homolog (T californica) |
| Q9DCW4 | NA | 110826 | ENSMUSG00000004610 | Etfb | electron transferring flavoprotein, beta polypeptide |
| Q61171 | NA | 21672 | ENSMUSG00000005161 | Prdx2 | peroxiredoxin 2 |
| Q61753 | NA | 236539 | ENSMUSG00000053398 | Phgdh | 3-phosphoglycerate dehydrogenase |
| Q922Q1 | NA | 67247 | ENSMUSG00000073481 | Mosc2 | MOCO sulphurase C-terminal domain containing 2 |
| B2RSR7 | NA | 333433 | ENSMUSG00000050627 | Gpd1l | glycerol-3-phosphate dehydrogenase 1-like |
| Q3ULJ0 | NA | 333433 | ENSMUSG00000050627 | Gpd1l | glycerol-3-phosphate dehydrogenase 1-like |
| **biological process----ensheathment of neurons----GO:0007272** | | | | | |
| C=35;O=4;E=0.13;R=31.81;rawP=7.13e-06;adjP=0.0005 | | | | | |
| P60202 | NA | 18823 | ENSMUSG00000031425 | Plp1 | proteolipid protein (myelin) 1 |
| Q3UYM8 | NA | 18823 | ENSMUSG00000031425 | Plp1 | proteolipid protein (myelin) 1 |
| P40240 | NA | 12527 | ENSMUSG00000030342 | Cd9 | CD9 antigen |
| Q60771 | NA | 18417 | ENSMUSG00000037625 | Cldn11 | claudin 11 |
| P04370 | NA | 17196 | ENSMUSG00000041607 | Mbp | myelin basic protein |
| **biological process----axon ensheathment----GO:0008366** | | | | | |
| C=35;O=4;E=0.13;R=31.81;rawP=7.13e-06;adjP=0.0005 | | | | | |
| P60202 | NA | 18823 | ENSMUSG00000031425 | Plp1 | proteolipid protein (myelin) 1 |
| Q3UYM8 | NA | 18823 | ENSMUSG00000031425 | Plp1 | proteolipid protein (myelin) 1 |
| P40240 | NA | 12527 | ENSMUSG00000030342 | Cd9 | CD9 antigen |
| Q60771 | NA | 18417 | ENSMUSG00000037625 | Cldn11 | claudin 11 |
| P04370 | NA | 17196 | ENSMUSG00000041607 | Mbp | myelin basic protein |
| **biological process----regulation of action potential in neuron----GO:0019228** | | | | | |
| C=39;O=4;E=0.14;R=28.54;rawP=1.11e-05;adjP=0.0006 | | | | | |
| P60202 | NA | 18823 | ENSMUSG00000031425 | Plp1 | proteolipid protein (myelin) 1 |
| Q3UYM8 | NA | 18823 | ENSMUSG00000031425 | Plp1 | proteolipid protein (myelin) 1 |
| P40240 | NA | 12527 | ENSMUSG00000030342 | Cd9 | CD9 antigen |
| Q60771 | NA | 18417 | ENSMUSG00000037625 | Cldn11 | claudin 11 |
| P04370 | NA | 17196 | ENSMUSG00000041607 | Mbp | myelin basic protein |
| **biological process----cellular macromolecular complex assembly----GO:0034622** | | | | | |
| C=230;O=7;E=0.83;R=8.47;rawP=1.69e-05;adjP=0.0008 | | | | | |
| P68372 | NA | 227613 | ENSMUSG00000036752 | Tubb2c | tubulin, beta 2C |
| P10922 | NA | 14958 | NULL | H1f0 | H1 histone family, member 0 |
| P99024 | NA | 22154 | ENSMUSG00000001525 | Tubb5 | tubulin, beta 5 |
| Q8VCM7 | NA | 99571 | ENSMUSG00000033860 | Fgg | fibrinogen gamma chain |
| Q3UEM7 | NA | 99571 | ENSMUSG00000033860 | Fgg | fibrinogen gamma chain |
| P43274 | NA | 50709 | ENSMUSG00000051627 | Hist1h1e | histone cluster 1, H1e |
| Q9D6F9 | NA | 22153 | ENSMUSG00000062591 | Tubb4 | tubulin, beta 4 |
| P15864 | NA | 50708 | ENSMUSG00000036181 | Hist1h1c | histone cluster 1, H1c |
| Q5SZA3 | NA | 50708 | ENSMUSG00000036181 | Hist1h1c | histone cluster 1, H1c |
| **biological process----regulation of action potential----GO:0001508** | | | | | |
| C=47;O=4;E=0.17;R=23.69;rawP=2.35e-05;adjP=0.0009 | | | | | |
| P60202 | NA | 18823 | ENSMUSG00000031425 | Plp1 | proteolipid protein (myelin) 1 |
| Q3UYM8 | NA | 18823 | ENSMUSG00000031425 | Plp1 | proteolipid protein (myelin) 1 |
| P40240 | NA | 12527 | ENSMUSG00000030342 | Cd9 | CD9 antigen |
| Q60771 | NA | 18417 | ENSMUSG00000037625 | Cldn11 | claudin 11 |
| P04370 | NA | 17196 | ENSMUSG00000041607 | Mbp | myelin basic protein |
| **biological process----nucleosome positioning----GO:0016584** | | | | | |
| C=3;O=2;E=0.01;R=185.54;rawP=3.79e-05;adjP=0.0010 | | | | | |
| P43274 | NA | 50709 | ENSMUSG00000051627 | Hist1h1e | histone cluster 1, H1e |
| P15864 | NA | 50708 | ENSMUSG00000036181 | Hist1h1c | histone cluster 1, H1c |
| Q5SZA3 | NA | 50708 | ENSMUSG00000036181 | Hist1h1c | histone cluster 1, H1c |
| **biological process----cellular component assembly----GO:0022607** | | | | | |
| C=468;O=9;E=1.68;R=5.35;rawP=3.78e-05;adjP=0.0010 | | | | | |
| Q8VCM7 | NA | 99571 | ENSMUSG00000033860 | Fgg | fibrinogen gamma chain |
| Q3UEM7 | NA | 99571 | ENSMUSG00000033860 | Fgg | fibrinogen gamma chain |
| P43274 | NA | 50709 | ENSMUSG00000051627 | Hist1h1e | histone cluster 1, H1e |
| Q6GT24 | NA | 11758 | ENSMUSG00000026701 | Prdx6 | peroxiredoxin 6 |
| O08709 | NA | 11758 | ENSMUSG00000026701 | Prdx6 | peroxiredoxin 6 |
| P15864 | NA | 50708 | ENSMUSG00000036181 | Hist1h1c | histone cluster 1, H1c |
| Q5SZA3 | NA | 50708 | ENSMUSG00000036181 | Hist1h1c | histone cluster 1, H1c |
| P68372 | NA | 227613 | ENSMUSG00000036752 | Tubb2c | tubulin, beta 2C |
| P10922 | NA | 14958 | NULL | H1f0 | H1 histone family, member 0 |
| P40240 | NA | 12527 | ENSMUSG00000030342 | Cd9 | CD9 antigen |
| P99024 | NA | 22154 | ENSMUSG00000001525 | Tubb5 | tubulin, beta 5 |
| Q9D6F9 | NA | 22153 | ENSMUSG00000062591 | Tubb4 | tubulin, beta 4 |
| **biological process----cellular macromolecular complex subunit organization----GO:0034621** | | | | | |
| C=262;O=7;E=0.94;R=7.44;rawP=3.90e-05;adjP=0.0010 | | | | | |
| P68372 | NA | 227613 | ENSMUSG00000036752 | Tubb2c | tubulin, beta 2C |
| P10922 | NA | 14958 | NULL | H1f0 | H1 histone family, member 0 |
| P99024 | NA | 22154 | ENSMUSG00000001525 | Tubb5 | tubulin, beta 5 |
| Q8VCM7 | NA | 99571 | ENSMUSG00000033860 | Fgg | fibrinogen gamma chain |
| Q3UEM7 | NA | 99571 | ENSMUSG00000033860 | Fgg | fibrinogen gamma chain |
| P43274 | NA | 50709 | ENSMUSG00000051627 | Hist1h1e | histone cluster 1, H1e |
| Q9D6F9 | NA | 22153 | ENSMUSG00000062591 | Tubb4 | tubulin, beta 4 |
| P15864 | NA | 50708 | ENSMUSG00000036181 | Hist1h1c | histone cluster 1, H1c |
| Q5SZA3 | NA | 50708 | ENSMUSG00000036181 | Hist1h1c | histone cluster 1, H1c |
| **biological process----cellular homeostasis----GO:0019725** | | | | | |
| C=292;O=7;E=1.05;R=6.67;rawP=7.74e-05;adjP=0.0018 | | | | | |
| Q61171 | NA | 21672 | ENSMUSG00000005161 | Prdx2 | peroxiredoxin 2 |
| P60202 | NA | 18823 | ENSMUSG00000031425 | Plp1 | proteolipid protein (myelin) 1 |
| Q3UYM8 | NA | 18823 | ENSMUSG00000031425 | Plp1 | proteolipid protein (myelin) 1 |
| P40240 | NA | 12527 | ENSMUSG00000030342 | Cd9 | CD9 antigen |
| Q60771 | NA | 18417 | ENSMUSG00000037625 | Cldn11 | claudin 11 |
| P26883 | NA | 14225 | ENSMUSG00000032966 | Fkbp1a | FK506 binding protein 1a |
| A2AT05 | NA | 14225 | ENSMUSG00000032966 | Fkbp1a | FK506 binding protein 1a |
| Q6GT24 | NA | 11758 | ENSMUSG00000026701 | Prdx6 | peroxiredoxin 6 |
| O08709 | NA | 11758 | ENSMUSG00000026701 | Prdx6 | peroxiredoxin 6 |
| P04370 | NA | 17196 | ENSMUSG00000041607 | Mbp | myelin basic protein |
| **molecular function----structural molecule activity----GO:0005198** | | | | | |
| C=370;O=10;E=1.43;R=6.98;rawP=1.36e-06;adjP=9.38e-05 | | | | | |
| P46660 | NA | 226180 | ENSMUSG00000034336 | Ina | internexin neuronal intermediate filament protein, alpha |
| Q9D2P8 | NA | 17433 | ENSMUSG00000032517 | Mobp | myelin-associated oligodendrocytic basic protein |
| P04370 | NA | 17196 | ENSMUSG00000041607 | Mbp | myelin basic protein |
| P68372 | NA | 227613 | ENSMUSG00000036752 | Tubb2c | tubulin, beta 2C |
| P47963 | NA | 270106 | ENSMUSG00000000740 | Rpl13 | ribosomal protein L13 |
| Q5RKP3 | NA | 270106 | ENSMUSG00000000740 | Rpl13 | ribosomal protein L13 |
| P08122 | NA | 12827 | ENSMUSG00000031503 | Col4a2 | collagen, type IV, alpha 2 |
| B2RQQ8 | NA | 12827 | ENSMUSG00000031503 | Col4a2 | collagen, type IV, alpha 2 |
| Q60771 | NA | 18417 | ENSMUSG00000037625 | Cldn11 | claudin 11 |
| P99024 | NA | 22154 | ENSMUSG00000001525 | Tubb5 | tubulin, beta 5 |
| Q5FWJ3 | NA | 22352 | ENSMUSG00000026728 | Vim | vimentin |
| P20152 | NA | 22352 | ENSMUSG00000026728 | Vim | vimentin |
| Q9D6F9 | NA | 22153 | ENSMUSG00000062591 | Tubb4 | tubulin, beta 4 |
| **molecular function----structural constituent of myelin sheath----GO:0019911** | | | | | |
| C=2;O=2;E=0.01;R=258.19;rawP=1.47e-05;adjP=0.0005 | | | | | |
| Q9D2P8 | NA | 17433 | ENSMUSG00000032517 | Mobp | myelin-associated oligodendrocytic basic protein |
| P04370 | NA | 17196 | ENSMUSG00000041607 | Mbp | myelin basic protein |
| **molecular function----oxidoreductase activity----GO:0016491** | | | | | |
| C=662;O=10;E=2.56;R=3.90;rawP=0.0002;adjP=0.0046 | | | | | |
| Q544B1 | NA | 11669 | ENSMUSG00000029455 | Aldh2 | aldehyde dehydrogenase 2, mitochondrial |
| P47738 | NA | 11669 | ENSMUSG00000029455 | Aldh2 | aldehyde dehydrogenase 2, mitochondrial |
| Q64521 | NA | 14571 | ENSMUSG00000026827 | Gpd2 | glycerol phosphate dehydrogenase 2, mitochondrial |
| Q9CQ69 | NA | 22272 | ENSMUSG00000044894 | Uqcrq | ubiquinol-cytochrome c reductase, complex III subunit VII |
| Q8BVI4 | NA | 110391 | ENSMUSG00000015806 | Qdpr | quinoid dihydropteridine reductase |
| Q6GT24 | NA | 11758 | ENSMUSG00000026701 | Prdx6 | peroxiredoxin 6 |
| O08709 | NA | 11758 | ENSMUSG00000026701 | Prdx6 | peroxiredoxin 6 |
| Q499X4 | NA | 26949 | ENSMUSG00000034993 | Vat1 | vesicle amine transport protein 1 homolog (T californica) |
| Q62465 | NA | 26949 | ENSMUSG00000034993 | Vat1 | vesicle amine transport protein 1 homolog (T californica) |
| Q61171 | NA | 21672 | ENSMUSG00000005161 | Prdx2 | peroxiredoxin 2 |
| Q922Q1 | NA | 67247 | ENSMUSG00000073481 | Mosc2 | MOCO sulphurase C-terminal domain containing 2 |
| Q61753 | NA | 236539 | ENSMUSG00000053398 | Phgdh | 3-phosphoglycerate dehydrogenase |
| B2RSR7 | NA | 333433 | ENSMUSG00000050627 | Gpd1l | glycerol-3-phosphate dehydrogenase 1-like |
| Q3ULJ0 | NA | 333433 | ENSMUSG00000050627 | Gpd1l | glycerol-3-phosphate dehydrogenase 1-like |
| **molecular function----peroxiredoxin activity----GO:0051920** | | | | | |
| C=7;O=2;E=0.03;R=73.77;rawP=0.0003;adjP=0.0052 | | | | | |
| Q61171 | NA | 21672 | ENSMUSG00000005161 | Prdx2 | peroxiredoxin 2 |
| Q6GT24 | NA | 11758 | ENSMUSG00000026701 | Prdx6 | peroxiredoxin 6 |
| O08709 | NA | 11758 | ENSMUSG00000026701 | Prdx6 | peroxiredoxin 6 |
| **molecular function----GTPase activity----GO:0003924** | | | | | |
| C=110;O=4;E=0.43;R=9.39;rawP=0.0009;adjP=0.0124 | | | | | |
| P08752 | NA | 14678 | ENSMUSG00000032562 | Gnai2 | guanine nucleotide binding protein (G protein), alpha inhibiting 2 |
| P68372 | NA | 227613 | ENSMUSG00000036752 | Tubb2c | tubulin, beta 2C |
| P99024 | NA | 22154 | ENSMUSG00000001525 | Tubb5 | tubulin, beta 5 |
| Q9D6F9 | NA | 22153 | ENSMUSG00000062591 | Tubb4 | tubulin, beta 4 |
| **molecular function----voltage-gated anion channel activity----GO:0008308** | | | | | |
| C=19;O=2;E=0.07;R=27.18;rawP=0.0024;adjP=0.0276 | | | | | |
| Q60930 | NA | 22334 | ENSMUSG00000021771 | Vdac2 | voltage-dependent anion channel 2 |
| Q60931 | NA | 22335 | ENSMUSG00000008892 | Vdac3 | voltage-dependent anion channel 3 |
| Q3TX38 | NA | 22335 | ENSMUSG00000008892 | Vdac3 | voltage-dependent anion channel 3 |
| **molecular function----peroxidase activity----GO:0004601** | | | | | |
| C=25;O=2;E=0.10;R=20.66;rawP=0.0042;adjP=0.0362 | | | | | |
| Q61171 | NA | 21672 | ENSMUSG00000005161 | Prdx2 | peroxiredoxin 2 |
| Q6GT24 | NA | 11758 | ENSMUSG00000026701 | Prdx6 | peroxiredoxin 6 |
| O08709 | NA | 11758 | ENSMUSG00000026701 | Prdx6 | peroxiredoxin 6 |
| **molecular function----oxidoreductase activity, acting on peroxide as acceptor----GO:0016684** | | | | | |
| C=25;O=2;E=0.10;R=20.66;rawP=0.0042;adjP=0.0362 | | | | | |
| Q61171 | NA | 21672 | ENSMUSG00000005161 | Prdx2 | peroxiredoxin 2 |
| Q6GT24 | NA | 11758 | ENSMUSG00000026701 | Prdx6 | peroxiredoxin 6 |
| O08709 | NA | 11758 | ENSMUSG00000026701 | Prdx6 | peroxiredoxin 6 |
| **molecular function----cofactor binding----GO:0048037** | | | | | |
| C=185;O=4;E=0.72;R=5.58;rawP=0.0057;adjP=0.0414 | | | | | |
| Q548W7 | NA | 13167 | ENSMUSG00000026385 | Dbi | diazepam binding inhibitor |
| P31786 | NA | 13167 | ENSMUSG00000026385 | Dbi | diazepam binding inhibitor |
| Q61753 | NA | 236539 | ENSMUSG00000053398 | Phgdh | 3-phosphoglycerate dehydrogenase |
| Q922Q1 | NA | 67247 | ENSMUSG00000073481 | Mosc2 | MOCO sulphurase C-terminal domain containing 2 |
| B2RSR7 | NA | 333433 | ENSMUSG00000050627 | Gpd1l | glycerol-3-phosphate dehydrogenase 1-like |
| Q3ULJ0 | NA | 333433 | ENSMUSG00000050627 | Gpd1l | glycerol-3-phosphate dehydrogenase 1-like |
| **molecular function----NAD or NADH binding----GO:0051287** | | | | | |
| C=30;O=2;E=0.12;R=17.21;rawP=0.0060;adjP=0.0414 | | | | | |
| Q61753 | NA | 236539 | ENSMUSG00000053398 | Phgdh | 3-phosphoglycerate dehydrogenase |
| B2RSR7 | NA | 333433 | ENSMUSG00000050627 | Gpd1l | glycerol-3-phosphate dehydrogenase 1-like |
| Q3ULJ0 | NA | 333433 | ENSMUSG00000050627 | Gpd1l | glycerol-3-phosphate dehydrogenase 1-like |
| **cellular component----myelin sheath----GO:0043209** | | | | | |
| C=8;O=4;E=0.03;R=139.99;rawP=1.01e-08;adjP=7.57e-07 | | | | | |
| P60202 | NA | 18823 | ENSMUSG00000031425 | Plp1 | proteolipid protein (myelin) 1 |
| Q3UYM8 | NA | 18823 | ENSMUSG00000031425 | Plp1 | proteolipid protein (myelin) 1 |
| P20917 | NA | 17136 | ENSMUSG00000036634 | Mag | myelin-associated glycoprotein |
| Q9D6F9 | NA | 22153 | ENSMUSG00000062591 | Tubb4 | tubulin, beta 4 |
| P04370 | NA | 17196 | ENSMUSG00000041607 | Mbp | myelin basic protein |
| **cellular component----basement membrane----GO:0005604** | | | | | |
| C=69;O=5;E=0.25;R=20.29;rawP=4.57e-06;adjP=0.0001 | | | | | |
| Q3USI2 | NA | 16779 | ENSMUSG00000052911 | Lamb2 | laminin, beta 2 |
| P08122 | NA | 12827 | ENSMUSG00000031503 | Col4a2 | collagen, type IV, alpha 2 |
| B2RQQ8 | NA | 12827 | ENSMUSG00000031503 | Col4a2 | collagen, type IV, alpha 2 |
| P10493 | NA | 18073 | ENSMUSG00000005397 | Nid1 | nidogen 1 |
| Q3UHH3 | NA | 15530 | NULL | Hspg2 | perlecan (heparan sulfate proteoglycan 2) |
| Q2XQV0 | NA | 15530 | NULL | Hspg2 | perlecan (heparan sulfate proteoglycan 2) |
| Q05793 | NA | 15530 | NULL | Hspg2 | perlecan (heparan sulfate proteoglycan 2) |
| Q52KG8 | NA | 15530 | NULL | Hspg2 | perlecan (heparan sulfate proteoglycan 2) |
| Q3TPN0 | NA | 18074 | ENSMUSG00000021806 | Nid2 | nidogen 2 |
| Q8C6Z2 | NA | 18074 | ENSMUSG00000021806 | Nid2 | nidogen 2 |
| Q8R5G0 | NA | 18074 | ENSMUSG00000021806 | Nid2 | nidogen 2 |
| **cellular component----mitochondrial part----GO:0044429** | | | | | |
| C=400;O=9;E=1.43;R=6.30;rawP=1.10e-05;adjP=0.0001 | | | | | |
| Q64521 | NA | 14571 | ENSMUSG00000026827 | Gpd2 | glycerol phosphate dehydrogenase 2, mitochondrial |
| Q9CQ69 | NA | 22272 | ENSMUSG00000044894 | Uqcrq | ubiquinol-cytochrome c reductase, complex III subunit VII |
| Q60931 | NA | 22335 | ENSMUSG00000008892 | Vdac3 | voltage-dependent anion channel 3 |
| Q3TX38 | NA | 22335 | ENSMUSG00000008892 | Vdac3 | voltage-dependent anion channel 3 |
| O35129 | NA | 12034 | ENSMUSG00000004264 | Phb2 | prohibitin 2 |
| Q3V235 | NA | 12034 | ENSMUSG00000004264 | Phb2 | prohibitin 2 |
| Q9DCW4 | NA | 110826 | ENSMUSG00000004610 | Etfb | electron transferring flavoprotein, beta polypeptide |
| Q9WUM5 | NA | 56451 | ENSMUSG00000052738 | Suclg1 | succinate-CoA ligase, GDP-forming, alpha subunit |
| Q60930 | NA | 22334 | ENSMUSG00000021771 | Vdac2 | voltage-dependent anion channel 2 |
| Q8BWT1 | NA | 52538 | ENSMUSG00000036880 | Acaa2 | acetyl-Coenzyme A acyltransferase 2 (mitochondrial 3-oxoacyl-Coenzyme A thiolase) |
| Q922Q1 | NA | 67247 | ENSMUSG00000073481 | Mosc2 | MOCO sulphurase C-terminal domain containing 2 |
| **cellular component----mitochondrial inner membrane----GO:0005743** | | | | | |
| C=282;O=8;E=1.01;R=7.94;rawP=6.66e-06;adjP=0.0001 | | | | | |
| Q64521 | NA | 14571 | ENSMUSG00000026827 | Gpd2 | glycerol phosphate dehydrogenase 2, mitochondrial |
| Q9CQ69 | NA | 22272 | ENSMUSG00000044894 | Uqcrq | ubiquinol-cytochrome c reductase, complex III subunit VII |
| Q60931 | NA | 22335 | ENSMUSG00000008892 | Vdac3 | voltage-dependent anion channel 3 |
| Q3TX38 | NA | 22335 | ENSMUSG00000008892 | Vdac3 | voltage-dependent anion channel 3 |
| O35129 | NA | 12034 | ENSMUSG00000004264 | Phb2 | prohibitin 2 |
| Q3V235 | NA | 12034 | ENSMUSG00000004264 | Phb2 | prohibitin 2 |
| Q9WUM5 | NA | 56451 | ENSMUSG00000052738 | Suclg1 | succinate-CoA ligase, GDP-forming, alpha subunit |
| Q60930 | NA | 22334 | ENSMUSG00000021771 | Vdac2 | voltage-dependent anion channel 2 |
| Q8BWT1 | NA | 52538 | ENSMUSG00000036880 | Acaa2 | acetyl-Coenzyme A acyltransferase 2 (mitochondrial 3-oxoacyl-Coenzyme A thiolase) |
| Q922Q1 | NA | 67247 | ENSMUSG00000073481 | Mosc2 | MOCO sulphurase C-terminal domain containing 2 |
| **cellular component----mitochondrion----GO:0005739** | | | | | |
| C=1310;O=16;E=4.68;R=3.42;rawP=8.62e-06;adjP=0.0001 | | | | | |
| Q544B1 | NA | 11669 | ENSMUSG00000029455 | Aldh2 | aldehyde dehydrogenase 2, mitochondrial |
| P47738 | NA | 11669 | ENSMUSG00000029455 | Aldh2 | aldehyde dehydrogenase 2, mitochondrial |
| Q9D2P8 | NA | 17433 | ENSMUSG00000032517 | Mobp | myelin-associated oligodendrocytic basic protein |
| Q64521 | NA | 14571 | ENSMUSG00000026827 | Gpd2 | glycerol phosphate dehydrogenase 2, mitochondrial |
| Q9CQ69 | NA | 22272 | ENSMUSG00000044894 | Uqcrq | ubiquinol-cytochrome c reductase, complex III subunit VII |
| Q8BVI4 | NA | 110391 | ENSMUSG00000015806 | Qdpr | quinoid dihydropteridine reductase |
| Q60931 | NA | 22335 | ENSMUSG00000008892 | Vdac3 | voltage-dependent anion channel 3 |
| Q3TX38 | NA | 22335 | ENSMUSG00000008892 | Vdac3 | voltage-dependent anion channel 3 |
| Q6GT24 | NA | 11758 | ENSMUSG00000026701 | Prdx6 | peroxiredoxin 6 |
| O08709 | NA | 11758 | ENSMUSG00000026701 | Prdx6 | peroxiredoxin 6 |
| O35129 | NA | 12034 | ENSMUSG00000004264 | Phb2 | prohibitin 2 |
| Q3V235 | NA | 12034 | ENSMUSG00000004264 | Phb2 | prohibitin 2 |
| Q56A15 | NA | 13063 | ENSMUSG00000063694 | Cycs | cytochrome c, somatic |
| P62897 | NA | 13063 | ENSMUSG00000063694 | Cycs | cytochrome c, somatic |
| Q9DCW4 | NA | 110826 | ENSMUSG00000004610 | Etfb | electron transferring flavoprotein, beta polypeptide |
| Q61171 | NA | 21672 | ENSMUSG00000005161 | Prdx2 | peroxiredoxin 2 |
| Q9WUM5 | NA | 56451 | ENSMUSG00000052738 | Suclg1 | succinate-CoA ligase, GDP-forming, alpha subunit |
| Q548W7 | NA | 13167 | ENSMUSG00000026385 | Dbi | diazepam binding inhibitor |
| P31786 | NA | 13167 | ENSMUSG00000026385 | Dbi | diazepam binding inhibitor |
| Q60930 | NA | 22334 | ENSMUSG00000021771 | Vdac2 | voltage-dependent anion channel 2 |
| Q8BWT1 | NA | 52538 | ENSMUSG00000036880 | Acaa2 | acetyl-Coenzyme A acyltransferase 2 (mitochondrial 3-oxoacyl-Coenzyme A thiolase) |
| Q922Q1 | NA | 67247 | ENSMUSG00000073481 | Mosc2 | MOCO sulphurase C-terminal domain containing 2 |
| **cellular component----organelle inner membrane----GO:0019866** | | | | | |
| C=295;O=8;E=1.05;R=7.59;rawP=9.26e-06;adjP=0.0001 | | | | | |
| Q64521 | NA | 14571 | ENSMUSG00000026827 | Gpd2 | glycerol phosphate dehydrogenase 2, mitochondrial |
| Q9CQ69 | NA | 22272 | ENSMUSG00000044894 | Uqcrq | ubiquinol-cytochrome c reductase, complex III subunit VII |
| Q60931 | NA | 22335 | ENSMUSG00000008892 | Vdac3 | voltage-dependent anion channel 3 |
| Q3TX38 | NA | 22335 | ENSMUSG00000008892 | Vdac3 | voltage-dependent anion channel 3 |
| O35129 | NA | 12034 | ENSMUSG00000004264 | Phb2 | prohibitin 2 |
| Q3V235 | NA | 12034 | ENSMUSG00000004264 | Phb2 | prohibitin 2 |
| Q9WUM5 | NA | 56451 | ENSMUSG00000052738 | Suclg1 | succinate-CoA ligase, GDP-forming, alpha subunit |
| Q60930 | NA | 22334 | ENSMUSG00000021771 | Vdac2 | voltage-dependent anion channel 2 |
| Q8BWT1 | NA | 52538 | ENSMUSG00000036880 | Acaa2 | acetyl-Coenzyme A acyltransferase 2 (mitochondrial 3-oxoacyl-Coenzyme A thiolase) |
| Q922Q1 | NA | 67247 | ENSMUSG00000073481 | Mosc2 | MOCO sulphurase C-terminal domain containing 2 |
| **cellular component----tubulin complex----GO:0045298** | | | | | |
| C=2;O=2;E=0.01;R=279.98;rawP=1.25e-05;adjP=0.0001 | | | | | |
| P68372 | NA | 227613 | ENSMUSG00000036752 | Tubb2c | tubulin, beta 2C |
| P99024 | NA | 22154 | ENSMUSG00000001525 | Tubb5 | tubulin, beta 5 |
| **cellular component----extracellular matrix part----GO:0044420** | | | | | |
| C=86;O=5;E=0.31;R=16.28;rawP=1.35e-05;adjP=0.0001 | | | | | |
| Q3USI2 | NA | 16779 | ENSMUSG00000052911 | Lamb2 | laminin, beta 2 |
| P08122 | NA | 12827 | ENSMUSG00000031503 | Col4a2 | collagen, type IV, alpha 2 |
| B2RQQ8 | NA | 12827 | ENSMUSG00000031503 | Col4a2 | collagen, type IV, alpha 2 |
| P10493 | NA | 18073 | ENSMUSG00000005397 | Nid1 | nidogen 1 |
| Q3UHH3 | NA | 15530 | NULL | Hspg2 | perlecan (heparan sulfate proteoglycan 2) |
| Q2XQV0 | NA | 15530 | NULL | Hspg2 | perlecan (heparan sulfate proteoglycan 2) |
| Q05793 | NA | 15530 | NULL | Hspg2 | perlecan (heparan sulfate proteoglycan 2) |
| Q52KG8 | NA | 15530 | NULL | Hspg2 | perlecan (heparan sulfate proteoglycan 2) |
| Q3TPN0 | NA | 18074 | ENSMUSG00000021806 | Nid2 | nidogen 2 |
| Q8C6Z2 | NA | 18074 | ENSMUSG00000021806 | Nid2 | nidogen 2 |
| Q8R5G0 | NA | 18074 | ENSMUSG00000021806 | Nid2 | nidogen 2 |
| **cellular component----non-membrane-bounded organelle----GO:0043228** | | | | | |
| C=1610;O=17;E=5.75;R=2.96;rawP=2.84e-05;adjP=0.0002 | | | | | |
| Q6S388 | NA | 18810 | ENSMUSG00000022565 | Plec1 | plectin 1 |
| Q7TSJ2 | NA | 17760 | ENSMUSG00000055407 | Mtap6 | microtubule-associated protein 6 |
| P43274 | NA | 50709 | ENSMUSG00000051627 | Hist1h1e | histone cluster 1, H1e |
| Q8BH44 | NA | 235431 | ENSMUSG00000041729 | Coro2b | coronin, actin binding protein, 2B |
| Q9JJV2 | NA | 18645 | ENSMUSG00000027805 | Pfn2 | profilin 2 |
| P68372 | NA | 227613 | ENSMUSG00000036752 | Tubb2c | tubulin, beta 2C |
| P47963 | NA | 270106 | ENSMUSG00000000740 | Rpl13 | ribosomal protein L13 |
| Q5RKP3 | NA | 270106 | ENSMUSG00000000740 | Rpl13 | ribosomal protein L13 |
| P10922 | NA | 14958 | NULL | H1f0 | H1 histone family, member 0 |
| P97315 | NA | 13007 | ENSMUSG00000026421 | Csrp1 | cysteine and glycine-rich protein 1 |
| Q4FJX4 | NA | 13007 | ENSMUSG00000026421 | Csrp1 | cysteine and glycine-rich protein 1 |
| Q53ZN9 | NA | 20533 | ENSMUSG00000006574 | Slc4a1 | solute carrier family 4 (anion exchanger), member 1 |
| P04919 | NA | 20533 | ENSMUSG00000006574 | Slc4a1 | solute carrier family 4 (anion exchanger), member 1 |
| Q3TZ29 | NA | 20533 | ENSMUSG00000006574 | Slc4a1 | solute carrier family 4 (anion exchanger), member 1 |
| Q9D6F9 | NA | 22153 | ENSMUSG00000062591 | Tubb4 | tubulin, beta 4 |
| P26041 | NA | 17698 | NULL | Msn | moesin |
| Q5FWJ3 | NA | 22352 | ENSMUSG00000026728 | Vim | vimentin |
| P20152 | NA | 22352 | ENSMUSG00000026728 | Vim | vimentin |
| P46660 | NA | 226180 | ENSMUSG00000034336 | Ina | internexin neuronal intermediate filament protein, alpha |
| A2ARP8 | NA | 17754 | ENSMUSG00000027254 | Mtap1a | microtubule-associated protein 1 A |
| P15864 | NA | 50708 | ENSMUSG00000036181 | Hist1h1c | histone cluster 1, H1c |
| Q5SZA3 | NA | 50708 | ENSMUSG00000036181 | Hist1h1c | histone cluster 1, H1c |
| P99024 | NA | 22154 | ENSMUSG00000001525 | Tubb5 | tubulin, beta 5 |
| **cellular component----axon part----GO:0033267** | | | | | |
| C=18;O=3;E=0.06;R=46.66;rawP=3.39e-05;adjP=0.0002 | | | | | |
| O54991 | NA | 53321 | ENSMUSG00000017167 | Cntnap1 | contactin associated protein-like 1 |
| Q9D6F9 | NA | 22153 | ENSMUSG00000062591 | Tubb4 | tubulin, beta 4 |
| P04370 | NA | 17196 | ENSMUSG00000041607 | Mbp | myelin basic protein |
