# Supplementary material for: Impact of Age on the Cerebrovascular Proteomes of Wild-Type and Tg-SwDI Mice
Source: PLoS One. 2014 Feb 26;9(2):e89970. doi: 10.1371/journal.pone.0089970 (PMC3935958; doi:10.1371/journal.pone.0089970)
Supplement: Table S3 — Gene ontology analysis of proteins found to be significantly different at p<0.01 within the Tg-SwDI cohort were uploaded to WebGestalt Gene Set Analysis Toolkit version 2. (HTML) [file pone.0089970.s006.html]

Anchored HTML File of EIDs


|  |  |  |  |  |  |
| --- | --- | --- | --- | --- | --- |
| **User file and parameters:** User file: Tg\_swDI\_3vs9mos\_p01\_webgestalt.txt, Organism: mmusculus, Id Type: uniprot\_swissprot\_accession, Ref Set: entrezgene, Significance Level: Top10, Statistics Test: Hypergeometric, MTC: BH, Minimum: 2  The results for each enriched GO category are listed in this table. For each GO category, the first row lists its sub-root (biological process, molecular function, or cellular component), category name, and corresponding GO ID. The second row lists number of reference genes in the category (C), number of genes in the gene set and also in the category (O), expected number in the category (E), Ratio of enrichment (R), p value from hypergeometric test (rawP), and p value adjusted by the multiple test adjustment (adjP). Finally, genes in the category are listed. For each gene, the table lists the user uploaded ID and value (optional), Entrez ID, Ensembl Gene Stable ID, Gene symbol, and description. Ensembl Gene Stable ID and Entrez Gene ID are linked to the Ensembl and Entrez Gene databases, respectively. | | | | | |
| **biological process----mRNA metabolic process----GO:0016071** | | | | | |
| C=282;O=12;E=0.72;R=16.78;rawP=2.45e-12;adjP=3.77e-10 | | | | | |
| Q60668 | NA | 11991 | ENSMUSG00000000568 | Hnrnpd | heterogeneous nuclear ribonucleoprotein D |
| Q544R5 | NA | 230908 | ENSMUSG00000041459 | Tardbp | TAR DNA binding protein |
| Q921F2 | NA | 230908 | ENSMUSG00000041459 | Tardbp | TAR DNA binding protein |
| Q9Z0H4 | NA | 14007 | ENSMUSG00000002107 | Cugbp2 | CUG triplet repeat, RNA binding protein 2 |
| Q91Z31 | NA | 56195 | ENSMUSG00000028134 | Ptbp2 | polypyrimidine tract binding protein 2 |
| Q3UMT7 | NA | 15388 | ENSMUSG00000015165 | Hnrnpl | heterogeneous nuclear ribonucleoprotein L |
| Q8R081 | NA | 15388 | ENSMUSG00000015165 | Hnrnpl | heterogeneous nuclear ribonucleoprotein L |
| Q9CVU5 | NA | 15388 | ENSMUSG00000015165 | Hnrnpl | heterogeneous nuclear ribonucleoprotein L |
| Q91VM5 | NA | 19656 | ENSMUSG00000037070 | Rbmxrt | RNA binding motif protein, X chromosome retrogene |
| P61979 | NA | 15387 | NULL | Hnrnpk | heterogeneous nuclear ribonucleoprotein K |
| O35737 | NA | 59013 | ENSMUSG00000007850 | Hnrnph1 | heterogeneous nuclear ribonucleoprotein H1 |
| Q811L7 | NA | 59013 | ENSMUSG00000007850 | Hnrnph1 | heterogeneous nuclear ribonucleoprotein H1 |
| O88569 | NA | 53379 | ENSMUSG00000004980 | Hnrnpa2b1 | heterogeneous nuclear ribonucleoprotein A2/B1 |
| Q3THA6 | NA | 225027 | ENSMUSG00000024097 | Sfrs7 | splicing factor, arginine/serine-rich 7 |
| Q8BL97 | NA | 225027 | ENSMUSG00000024097 | Sfrs7 | splicing factor, arginine/serine-rich 7 |
| P49312 | NA | 15382 | ENSMUSG00000046434 | Hnrnpa1 | heterogeneous nuclear ribonucleoprotein A1 |
| Q9Z204 | NA | 15381 | ENSMUSG00000060373 | Hnrnpc | heterogeneous nuclear ribonucleoprotein C |
| **biological process----mRNA processing----GO:0006397** | | | | | |
| C=249;O=11;E=0.63;R=17.42;rawP=1.58e-11;adjP=1.22e-09 | | | | | |
| Q544R5 | NA | 230908 | ENSMUSG00000041459 | Tardbp | TAR DNA binding protein |
| Q921F2 | NA | 230908 | ENSMUSG00000041459 | Tardbp | TAR DNA binding protein |
| Q9Z0H4 | NA | 14007 | ENSMUSG00000002107 | Cugbp2 | CUG triplet repeat, RNA binding protein 2 |
| Q91Z31 | NA | 56195 | ENSMUSG00000028134 | Ptbp2 | polypyrimidine tract binding protein 2 |
| Q3UMT7 | NA | 15388 | ENSMUSG00000015165 | Hnrnpl | heterogeneous nuclear ribonucleoprotein L |
| Q8R081 | NA | 15388 | ENSMUSG00000015165 | Hnrnpl | heterogeneous nuclear ribonucleoprotein L |
| Q9CVU5 | NA | 15388 | ENSMUSG00000015165 | Hnrnpl | heterogeneous nuclear ribonucleoprotein L |
| Q91VM5 | NA | 19656 | ENSMUSG00000037070 | Rbmxrt | RNA binding motif protein, X chromosome retrogene |
| P61979 | NA | 15387 | NULL | Hnrnpk | heterogeneous nuclear ribonucleoprotein K |
| O35737 | NA | 59013 | ENSMUSG00000007850 | Hnrnph1 | heterogeneous nuclear ribonucleoprotein H1 |
| Q811L7 | NA | 59013 | ENSMUSG00000007850 | Hnrnph1 | heterogeneous nuclear ribonucleoprotein H1 |
| O88569 | NA | 53379 | ENSMUSG00000004980 | Hnrnpa2b1 | heterogeneous nuclear ribonucleoprotein A2/B1 |
| Q3THA6 | NA | 225027 | ENSMUSG00000024097 | Sfrs7 | splicing factor, arginine/serine-rich 7 |
| Q8BL97 | NA | 225027 | ENSMUSG00000024097 | Sfrs7 | splicing factor, arginine/serine-rich 7 |
| P49312 | NA | 15382 | ENSMUSG00000046434 | Hnrnpa1 | heterogeneous nuclear ribonucleoprotein A1 |
| Q9Z204 | NA | 15381 | ENSMUSG00000060373 | Hnrnpc | heterogeneous nuclear ribonucleoprotein C |
| **biological process----RNA splicing----GO:0008380** | | | | | |
| C=192;O=10;E=0.49;R=20.54;rawP=3.04e-11;adjP=1.56e-09 | | | | | |
| Q544R5 | NA | 230908 | ENSMUSG00000041459 | Tardbp | TAR DNA binding protein |
| Q921F2 | NA | 230908 | ENSMUSG00000041459 | Tardbp | TAR DNA binding protein |
| Q9Z0H4 | NA | 14007 | ENSMUSG00000002107 | Cugbp2 | CUG triplet repeat, RNA binding protein 2 |
| Q91Z31 | NA | 56195 | ENSMUSG00000028134 | Ptbp2 | polypyrimidine tract binding protein 2 |
| Q91VM5 | NA | 19656 | ENSMUSG00000037070 | Rbmxrt | RNA binding motif protein, X chromosome retrogene |
| P61979 | NA | 15387 | NULL | Hnrnpk | heterogeneous nuclear ribonucleoprotein K |
| O35737 | NA | 59013 | ENSMUSG00000007850 | Hnrnph1 | heterogeneous nuclear ribonucleoprotein H1 |
| Q811L7 | NA | 59013 | ENSMUSG00000007850 | Hnrnph1 | heterogeneous nuclear ribonucleoprotein H1 |
| O88569 | NA | 53379 | ENSMUSG00000004980 | Hnrnpa2b1 | heterogeneous nuclear ribonucleoprotein A2/B1 |
| P49312 | NA | 15382 | ENSMUSG00000046434 | Hnrnpa1 | heterogeneous nuclear ribonucleoprotein A1 |
| Q3THA6 | NA | 225027 | ENSMUSG00000024097 | Sfrs7 | splicing factor, arginine/serine-rich 7 |
| Q8BL97 | NA | 225027 | ENSMUSG00000024097 | Sfrs7 | splicing factor, arginine/serine-rich 7 |
| Q9Z204 | NA | 15381 | ENSMUSG00000060373 | Hnrnpc | heterogeneous nuclear ribonucleoprotein C |
| **biological process----RNA processing----GO:0006396** | | | | | |
| C=389;O=12;E=0.99;R=12.16;rawP=1.05e-10;adjP=4.04e-09 | | | | | |
| Q544R5 | NA | 230908 | ENSMUSG00000041459 | Tardbp | TAR DNA binding protein |
| Q921F2 | NA | 230908 | ENSMUSG00000041459 | Tardbp | TAR DNA binding protein |
| Q9Z0H4 | NA | 14007 | ENSMUSG00000002107 | Cugbp2 | CUG triplet repeat, RNA binding protein 2 |
| Q564E6 | NA | 20823 | ENSMUSG00000068882 | Ssb | Sjogren syndrome antigen B |
| P32067 | NA | 20823 | ENSMUSG00000068882 | Ssb | Sjogren syndrome antigen B |
| Q91Z31 | NA | 56195 | ENSMUSG00000028134 | Ptbp2 | polypyrimidine tract binding protein 2 |
| Q3UMT7 | NA | 15388 | ENSMUSG00000015165 | Hnrnpl | heterogeneous nuclear ribonucleoprotein L |
| Q8R081 | NA | 15388 | ENSMUSG00000015165 | Hnrnpl | heterogeneous nuclear ribonucleoprotein L |
| Q9CVU5 | NA | 15388 | ENSMUSG00000015165 | Hnrnpl | heterogeneous nuclear ribonucleoprotein L |
| Q91VM5 | NA | 19656 | ENSMUSG00000037070 | Rbmxrt | RNA binding motif protein, X chromosome retrogene |
| P61979 | NA | 15387 | NULL | Hnrnpk | heterogeneous nuclear ribonucleoprotein K |
| O35737 | NA | 59013 | ENSMUSG00000007850 | Hnrnph1 | heterogeneous nuclear ribonucleoprotein H1 |
| Q811L7 | NA | 59013 | ENSMUSG00000007850 | Hnrnph1 | heterogeneous nuclear ribonucleoprotein H1 |
| O88569 | NA | 53379 | ENSMUSG00000004980 | Hnrnpa2b1 | heterogeneous nuclear ribonucleoprotein A2/B1 |
| Q3THA6 | NA | 225027 | ENSMUSG00000024097 | Sfrs7 | splicing factor, arginine/serine-rich 7 |
| Q8BL97 | NA | 225027 | ENSMUSG00000024097 | Sfrs7 | splicing factor, arginine/serine-rich 7 |
| P49312 | NA | 15382 | ENSMUSG00000046434 | Hnrnpa1 | heterogeneous nuclear ribonucleoprotein A1 |
| Q9Z204 | NA | 15381 | ENSMUSG00000060373 | Hnrnpc | heterogeneous nuclear ribonucleoprotein C |
| **biological process----RNA metabolic process----GO:0016070** | | | | | |
| C=1587;O=15;E=4.03;R=3.73;rawP=2.82e-06;adjP=8.69e-05 | | | | | |
| Q60668 | NA | 11991 | ENSMUSG00000000568 | Hnrnpd | heterogeneous nuclear ribonucleoprotein D |
| Q544R5 | NA | 230908 | ENSMUSG00000041459 | Tardbp | TAR DNA binding protein |
| Q921F2 | NA | 230908 | ENSMUSG00000041459 | Tardbp | TAR DNA binding protein |
| Q9Z0H4 | NA | 14007 | ENSMUSG00000002107 | Cugbp2 | CUG triplet repeat, RNA binding protein 2 |
| Q564E6 | NA | 20823 | ENSMUSG00000068882 | Ssb | Sjogren syndrome antigen B |
| P32067 | NA | 20823 | ENSMUSG00000068882 | Ssb | Sjogren syndrome antigen B |
| Q91Z31 | NA | 56195 | ENSMUSG00000028134 | Ptbp2 | polypyrimidine tract binding protein 2 |
| Q3UMT7 | NA | 15388 | ENSMUSG00000015165 | Hnrnpl | heterogeneous nuclear ribonucleoprotein L |
| Q8R081 | NA | 15388 | ENSMUSG00000015165 | Hnrnpl | heterogeneous nuclear ribonucleoprotein L |
| Q9CVU5 | NA | 15388 | ENSMUSG00000015165 | Hnrnpl | heterogeneous nuclear ribonucleoprotein L |
| Q91VM5 | NA | 19656 | ENSMUSG00000037070 | Rbmxrt | RNA binding motif protein, X chromosome retrogene |
| P61979 | NA | 15387 | NULL | Hnrnpk | heterogeneous nuclear ribonucleoprotein K |
| Q544Z3 | NA | 15384 | ENSMUSG00000020358 | Hnrnpab | heterogeneous nuclear ribonucleoprotein A/B |
| Q99020 | NA | 15384 | ENSMUSG00000020358 | Hnrnpab | heterogeneous nuclear ribonucleoprotein A/B |
| O35737 | NA | 59013 | ENSMUSG00000007850 | Hnrnph1 | heterogeneous nuclear ribonucleoprotein H1 |
| Q811L7 | NA | 59013 | ENSMUSG00000007850 | Hnrnph1 | heterogeneous nuclear ribonucleoprotein H1 |
| O88569 | NA | 53379 | ENSMUSG00000004980 | Hnrnpa2b1 | heterogeneous nuclear ribonucleoprotein A2/B1 |
| Q3THA6 | NA | 225027 | ENSMUSG00000024097 | Sfrs7 | splicing factor, arginine/serine-rich 7 |
| Q8BL97 | NA | 225027 | ENSMUSG00000024097 | Sfrs7 | splicing factor, arginine/serine-rich 7 |
| P49312 | NA | 15382 | ENSMUSG00000046434 | Hnrnpa1 | heterogeneous nuclear ribonucleoprotein A1 |
| Q9Z204 | NA | 15381 | ENSMUSG00000060373 | Hnrnpc | heterogeneous nuclear ribonucleoprotein C |
| Q5SX50 | NA | 18643 | ENSMUSG00000018293 | Pfn1 | profilin 1 |
| Q3U7V7 | NA | 18643 | ENSMUSG00000018293 | Pfn1 | profilin 1 |
| P62962 | NA | 18643 | ENSMUSG00000018293 | Pfn1 | profilin 1 |
| **biological process----gene expression----GO:0010467** | | | | | |
| C=2863;O=19;E=7.26;R=2.62;rawP=1.41e-05;adjP=0.0004 | | | | | |
| Q60668 | NA | 11991 | ENSMUSG00000000568 | Hnrnpd | heterogeneous nuclear ribonucleoprotein D |
| Q3UMT7 | NA | 15388 | ENSMUSG00000015165 | Hnrnpl | heterogeneous nuclear ribonucleoprotein L |
| Q8R081 | NA | 15388 | ENSMUSG00000015165 | Hnrnpl | heterogeneous nuclear ribonucleoprotein L |
| Q9CVU5 | NA | 15388 | ENSMUSG00000015165 | Hnrnpl | heterogeneous nuclear ribonucleoprotein L |
| Q544Z3 | NA | 15384 | ENSMUSG00000020358 | Hnrnpab | heterogeneous nuclear ribonucleoprotein A/B |
| Q99020 | NA | 15384 | ENSMUSG00000020358 | Hnrnpab | heterogeneous nuclear ribonucleoprotein A/B |
| O35737 | NA | 59013 | ENSMUSG00000007850 | Hnrnph1 | heterogeneous nuclear ribonucleoprotein H1 |
| Q811L7 | NA | 59013 | ENSMUSG00000007850 | Hnrnph1 | heterogeneous nuclear ribonucleoprotein H1 |
| O88569 | NA | 53379 | ENSMUSG00000004980 | Hnrnpa2b1 | heterogeneous nuclear ribonucleoprotein A2/B1 |
| P49312 | NA | 15382 | ENSMUSG00000046434 | Hnrnpa1 | heterogeneous nuclear ribonucleoprotein A1 |
| Q60749 | NA | 20218 | ENSMUSG00000028790 | Khdrbs1 | KH domain containing, RNA binding, signal transduction associated 1 |
| Q9Z0H4 | NA | 14007 | ENSMUSG00000002107 | Cugbp2 | CUG triplet repeat, RNA binding protein 2 |
| Q544R5 | NA | 230908 | ENSMUSG00000041459 | Tardbp | TAR DNA binding protein |
| Q921F2 | NA | 230908 | ENSMUSG00000041459 | Tardbp | TAR DNA binding protein |
| Q564E6 | NA | 20823 | ENSMUSG00000068882 | Ssb | Sjogren syndrome antigen B |
| P32067 | NA | 20823 | ENSMUSG00000068882 | Ssb | Sjogren syndrome antigen B |
| Q91Z31 | NA | 56195 | ENSMUSG00000028134 | Ptbp2 | polypyrimidine tract binding protein 2 |
| P08226 | NA | 11816 | ENSMUSG00000002985 | Apoe | apolipoprotein E |
| Q3TXU4 | NA | 11816 | ENSMUSG00000002985 | Apoe | apolipoprotein E |
| Q91VM5 | NA | 19656 | ENSMUSG00000037070 | Rbmxrt | RNA binding motif protein, X chromosome retrogene |
| P61979 | NA | 15387 | NULL | Hnrnpk | heterogeneous nuclear ribonucleoprotein K |
| Q61753 | NA | 236539 | ENSMUSG00000053398 | Phgdh | 3-phosphoglycerate dehydrogenase |
| Q3THA6 | NA | 225027 | ENSMUSG00000024097 | Sfrs7 | splicing factor, arginine/serine-rich 7 |
| Q8BL97 | NA | 225027 | ENSMUSG00000024097 | Sfrs7 | splicing factor, arginine/serine-rich 7 |
| Q9Z204 | NA | 15381 | ENSMUSG00000060373 | Hnrnpc | heterogeneous nuclear ribonucleoprotein C |
| Q5SX50 | NA | 18643 | ENSMUSG00000018293 | Pfn1 | profilin 1 |
| Q3U7V7 | NA | 18643 | ENSMUSG00000018293 | Pfn1 | profilin 1 |
| P62962 | NA | 18643 | ENSMUSG00000018293 | Pfn1 | profilin 1 |
| A7UQY4 | NA | 17876 | NULL | Myef2 | myelin basic protein expression factor 2, repressor |
| Q8C854 | NA | 17876 | NULL | Myef2 | myelin basic protein expression factor 2, repressor |
| **biological process----RNA splicing, via transesterification reactions----GO:0000375** | | | | | |
| C=30;O=3;E=0.08;R=39.43;rawP=5.80e-05;adjP=0.0010 | | | | | |
| Q9Z0H4 | NA | 14007 | ENSMUSG00000002107 | Cugbp2 | CUG triplet repeat, RNA binding protein 2 |
| Q91Z31 | NA | 56195 | ENSMUSG00000028134 | Ptbp2 | polypyrimidine tract binding protein 2 |
| P49312 | NA | 15382 | ENSMUSG00000046434 | Hnrnpa1 | heterogeneous nuclear ribonucleoprotein A1 |
| **biological process----mRNA splice site selection----GO:0006376** | | | | | |
| C=5;O=2;E=0.01;R=157.71;rawP=6.22e-05;adjP=0.0010 | | | | | |
| Q9Z0H4 | NA | 14007 | ENSMUSG00000002107 | Cugbp2 | CUG triplet repeat, RNA binding protein 2 |
| Q91Z31 | NA | 56195 | ENSMUSG00000028134 | Ptbp2 | polypyrimidine tract binding protein 2 |
| **biological process----RNA splicing, via transesterification reactions with bulged adenosine as nucleophile----GO:0000377** | | | | | |
| C=29;O=3;E=0.07;R=40.79;rawP=5.23e-05;adjP=0.0010 | | | | | |
| Q9Z0H4 | NA | 14007 | ENSMUSG00000002107 | Cugbp2 | CUG triplet repeat, RNA binding protein 2 |
| Q91Z31 | NA | 56195 | ENSMUSG00000028134 | Ptbp2 | polypyrimidine tract binding protein 2 |
| P49312 | NA | 15382 | ENSMUSG00000046434 | Hnrnpa1 | heterogeneous nuclear ribonucleoprotein A1 |
| **biological process----nuclear mRNA splicing, via spliceosome----GO:0000398** | | | | | |
| C=29;O=3;E=0.07;R=40.79;rawP=5.23e-05;adjP=0.0010 | | | | | |
| Q9Z0H4 | NA | 14007 | ENSMUSG00000002107 | Cugbp2 | CUG triplet repeat, RNA binding protein 2 |
| Q91Z31 | NA | 56195 | ENSMUSG00000028134 | Ptbp2 | polypyrimidine tract binding protein 2 |
| P49312 | NA | 15382 | ENSMUSG00000046434 | Hnrnpa1 | heterogeneous nuclear ribonucleoprotein A1 |
| **molecular function----RNA binding----GO:0003723** | | | | | |
| C=589;O=19;E=1.65;R=11.50;rawP=2.88e-16;adjP=8.93e-15 | | | | | |
| Q60668 | NA | 11991 | ENSMUSG00000000568 | Hnrnpd | heterogeneous nuclear ribonucleoprotein D |
| Q3TT81 | NA | 18521 | ENSMUSG00000056851 | Pcbp2 | poly(rC) binding protein 2 |
| Q61990 | NA | 18521 | ENSMUSG00000056851 | Pcbp2 | poly(rC) binding protein 2 |
| Q5BL18 | NA | 17184 | ENSMUSG00000037236 | Matr3 | matrin 3 |
| Q8K310 | NA | 17184 | ENSMUSG00000037236 | Matr3 | matrin 3 |
| Q3UMT7 | NA | 15388 | ENSMUSG00000015165 | Hnrnpl | heterogeneous nuclear ribonucleoprotein L |
| Q8R081 | NA | 15388 | ENSMUSG00000015165 | Hnrnpl | heterogeneous nuclear ribonucleoprotein L |
| Q9CVU5 | NA | 15388 | ENSMUSG00000015165 | Hnrnpl | heterogeneous nuclear ribonucleoprotein L |
| Q544Z3 | NA | 15384 | ENSMUSG00000020358 | Hnrnpab | heterogeneous nuclear ribonucleoprotein A/B |
| Q99020 | NA | 15384 | ENSMUSG00000020358 | Hnrnpab | heterogeneous nuclear ribonucleoprotein A/B |
| O35737 | NA | 59013 | ENSMUSG00000007850 | Hnrnph1 | heterogeneous nuclear ribonucleoprotein H1 |
| Q811L7 | NA | 59013 | ENSMUSG00000007850 | Hnrnph1 | heterogeneous nuclear ribonucleoprotein H1 |
| O88569 | NA | 53379 | ENSMUSG00000004980 | Hnrnpa2b1 | heterogeneous nuclear ribonucleoprotein A2/B1 |
| P49312 | NA | 15382 | ENSMUSG00000046434 | Hnrnpa1 | heterogeneous nuclear ribonucleoprotein A1 |
| Q60749 | NA | 20218 | ENSMUSG00000028790 | Khdrbs1 | KH domain containing, RNA binding, signal transduction associated 1 |
| P70333 | NA | 56258 | ENSMUSG00000045427 | Hnrnph2 | heterogeneous nuclear ribonucleoprotein H2 |
| A2BDV8 | NA | 56258 | ENSMUSG00000045427 | Hnrnph2 | heterogeneous nuclear ribonucleoprotein H2 |
| Q9Z0H4 | NA | 14007 | ENSMUSG00000002107 | Cugbp2 | CUG triplet repeat, RNA binding protein 2 |
| Q544R5 | NA | 230908 | ENSMUSG00000041459 | Tardbp | TAR DNA binding protein |
| Q921F2 | NA | 230908 | ENSMUSG00000041459 | Tardbp | TAR DNA binding protein |
| Q564E6 | NA | 20823 | ENSMUSG00000068882 | Ssb | Sjogren syndrome antigen B |
| P32067 | NA | 20823 | ENSMUSG00000068882 | Ssb | Sjogren syndrome antigen B |
| Q91Z31 | NA | 56195 | ENSMUSG00000028134 | Ptbp2 | polypyrimidine tract binding protein 2 |
| Q91VM5 | NA | 19656 | ENSMUSG00000037070 | Rbmxrt | RNA binding motif protein, X chromosome retrogene |
| P61979 | NA | 15387 | NULL | Hnrnpk | heterogeneous nuclear ribonucleoprotein K |
| Q3THA6 | NA | 225027 | ENSMUSG00000024097 | Sfrs7 | splicing factor, arginine/serine-rich 7 |
| Q8BL97 | NA | 225027 | ENSMUSG00000024097 | Sfrs7 | splicing factor, arginine/serine-rich 7 |
| Q9Z204 | NA | 15381 | ENSMUSG00000060373 | Hnrnpc | heterogeneous nuclear ribonucleoprotein C |
| A7UQY4 | NA | 17876 | NULL | Myef2 | myelin basic protein expression factor 2, repressor |
| Q8C854 | NA | 17876 | NULL | Myef2 | myelin basic protein expression factor 2, repressor |
| **molecular function----nucleic acid binding----GO:0003676** | | | | | |
| C=2395;O=21;E=6.72;R=3.13;rawP=3.08e-07;adjP=4.77e-06 | | | | | |
| Q60668 | NA | 11991 | ENSMUSG00000000568 | Hnrnpd | heterogeneous nuclear ribonucleoprotein D |
| Q3TT81 | NA | 18521 | ENSMUSG00000056851 | Pcbp2 | poly(rC) binding protein 2 |
| Q61990 | NA | 18521 | ENSMUSG00000056851 | Pcbp2 | poly(rC) binding protein 2 |
| Q5BL18 | NA | 17184 | ENSMUSG00000037236 | Matr3 | matrin 3 |
| Q8K310 | NA | 17184 | ENSMUSG00000037236 | Matr3 | matrin 3 |
| Q3UMT7 | NA | 15388 | ENSMUSG00000015165 | Hnrnpl | heterogeneous nuclear ribonucleoprotein L |
| Q8R081 | NA | 15388 | ENSMUSG00000015165 | Hnrnpl | heterogeneous nuclear ribonucleoprotein L |
| Q9CVU5 | NA | 15388 | ENSMUSG00000015165 | Hnrnpl | heterogeneous nuclear ribonucleoprotein L |
| Q544Z3 | NA | 15384 | ENSMUSG00000020358 | Hnrnpab | heterogeneous nuclear ribonucleoprotein A/B |
| Q99020 | NA | 15384 | ENSMUSG00000020358 | Hnrnpab | heterogeneous nuclear ribonucleoprotein A/B |
| O35737 | NA | 59013 | ENSMUSG00000007850 | Hnrnph1 | heterogeneous nuclear ribonucleoprotein H1 |
| Q811L7 | NA | 59013 | ENSMUSG00000007850 | Hnrnph1 | heterogeneous nuclear ribonucleoprotein H1 |
| O88569 | NA | 53379 | ENSMUSG00000004980 | Hnrnpa2b1 | heterogeneous nuclear ribonucleoprotein A2/B1 |
| P49312 | NA | 15382 | ENSMUSG00000046434 | Hnrnpa1 | heterogeneous nuclear ribonucleoprotein A1 |
| Q60749 | NA | 20218 | ENSMUSG00000028790 | Khdrbs1 | KH domain containing, RNA binding, signal transduction associated 1 |
| P70333 | NA | 56258 | ENSMUSG00000045427 | Hnrnph2 | heterogeneous nuclear ribonucleoprotein H2 |
| A2BDV8 | NA | 56258 | ENSMUSG00000045427 | Hnrnph2 | heterogeneous nuclear ribonucleoprotein H2 |
| Q9Z0H4 | NA | 14007 | ENSMUSG00000002107 | Cugbp2 | CUG triplet repeat, RNA binding protein 2 |
| Q544R5 | NA | 230908 | ENSMUSG00000041459 | Tardbp | TAR DNA binding protein |
| Q921F2 | NA | 230908 | ENSMUSG00000041459 | Tardbp | TAR DNA binding protein |
| Q564E6 | NA | 20823 | ENSMUSG00000068882 | Ssb | Sjogren syndrome antigen B |
| P32067 | NA | 20823 | ENSMUSG00000068882 | Ssb | Sjogren syndrome antigen B |
| Q91Z31 | NA | 56195 | ENSMUSG00000028134 | Ptbp2 | polypyrimidine tract binding protein 2 |
| Q91VM5 | NA | 19656 | ENSMUSG00000037070 | Rbmxrt | RNA binding motif protein, X chromosome retrogene |
| Q8VHM5 | NA | 74326 | ENSMUSG00000066037 | Hnrnpr | heterogeneous nuclear ribonucleoprotein R |
| P61979 | NA | 15387 | NULL | Hnrnpk | heterogeneous nuclear ribonucleoprotein K |
| Q3THA6 | NA | 225027 | ENSMUSG00000024097 | Sfrs7 | splicing factor, arginine/serine-rich 7 |
| Q8BL97 | NA | 225027 | ENSMUSG00000024097 | Sfrs7 | splicing factor, arginine/serine-rich 7 |
| Q8VEK3 | NA | 51810 | ENSMUSG00000039630 | Hnrnpu | heterogeneous nuclear ribonucleoprotein U |
| Q9Z204 | NA | 15381 | ENSMUSG00000060373 | Hnrnpc | heterogeneous nuclear ribonucleoprotein C |
| A7UQY4 | NA | 17876 | NULL | Myef2 | myelin basic protein expression factor 2, repressor |
| Q8C854 | NA | 17876 | NULL | Myef2 | myelin basic protein expression factor 2, repressor |
| **molecular function----nucleotide binding----GO:0000166** | | | | | |
| C=1955;O=17;E=5.48;R=3.10;rawP=8.68e-06;adjP=8.97e-05 | | | | | |
| Q60668 | NA | 11991 | ENSMUSG00000000568 | Hnrnpd | heterogeneous nuclear ribonucleoprotein D |
| Q5BL18 | NA | 17184 | ENSMUSG00000037236 | Matr3 | matrin 3 |
| Q8K310 | NA | 17184 | ENSMUSG00000037236 | Matr3 | matrin 3 |
| Q3UMT7 | NA | 15388 | ENSMUSG00000015165 | Hnrnpl | heterogeneous nuclear ribonucleoprotein L |
| Q8R081 | NA | 15388 | ENSMUSG00000015165 | Hnrnpl | heterogeneous nuclear ribonucleoprotein L |
| Q9CVU5 | NA | 15388 | ENSMUSG00000015165 | Hnrnpl | heterogeneous nuclear ribonucleoprotein L |
| Q544Z3 | NA | 15384 | ENSMUSG00000020358 | Hnrnpab | heterogeneous nuclear ribonucleoprotein A/B |
| Q99020 | NA | 15384 | ENSMUSG00000020358 | Hnrnpab | heterogeneous nuclear ribonucleoprotein A/B |
| O35737 | NA | 59013 | ENSMUSG00000007850 | Hnrnph1 | heterogeneous nuclear ribonucleoprotein H1 |
| Q811L7 | NA | 59013 | ENSMUSG00000007850 | Hnrnph1 | heterogeneous nuclear ribonucleoprotein H1 |
| O88569 | NA | 53379 | ENSMUSG00000004980 | Hnrnpa2b1 | heterogeneous nuclear ribonucleoprotein A2/B1 |
| P49312 | NA | 15382 | ENSMUSG00000046434 | Hnrnpa1 | heterogeneous nuclear ribonucleoprotein A1 |
| P70333 | NA | 56258 | ENSMUSG00000045427 | Hnrnph2 | heterogeneous nuclear ribonucleoprotein H2 |
| A2BDV8 | NA | 56258 | ENSMUSG00000045427 | Hnrnph2 | heterogeneous nuclear ribonucleoprotein H2 |
| Q9Z0H4 | NA | 14007 | ENSMUSG00000002107 | Cugbp2 | CUG triplet repeat, RNA binding protein 2 |
| Q544R5 | NA | 230908 | ENSMUSG00000041459 | Tardbp | TAR DNA binding protein |
| Q921F2 | NA | 230908 | ENSMUSG00000041459 | Tardbp | TAR DNA binding protein |
| Q564E6 | NA | 20823 | ENSMUSG00000068882 | Ssb | Sjogren syndrome antigen B |
| P32067 | NA | 20823 | ENSMUSG00000068882 | Ssb | Sjogren syndrome antigen B |
| Q91Z31 | NA | 56195 | ENSMUSG00000028134 | Ptbp2 | polypyrimidine tract binding protein 2 |
| Q91VM5 | NA | 19656 | ENSMUSG00000037070 | Rbmxrt | RNA binding motif protein, X chromosome retrogene |
| Q3THA6 | NA | 225027 | ENSMUSG00000024097 | Sfrs7 | splicing factor, arginine/serine-rich 7 |
| Q8BL97 | NA | 225027 | ENSMUSG00000024097 | Sfrs7 | splicing factor, arginine/serine-rich 7 |
| Q61753 | NA | 236539 | ENSMUSG00000053398 | Phgdh | 3-phosphoglycerate dehydrogenase |
| Q9Z204 | NA | 15381 | ENSMUSG00000060373 | Hnrnpc | heterogeneous nuclear ribonucleoprotein C |
| A7UQY4 | NA | 17876 | NULL | Myef2 | myelin basic protein expression factor 2, repressor |
| Q8C854 | NA | 17876 | NULL | Myef2 | myelin basic protein expression factor 2, repressor |
| **molecular function----structural molecule activity----GO:0005198** | | | | | |
| C=370;O=6;E=1.04;R=5.78;rawP=0.0005;adjP=0.0039 | | | | | |
| Q6PFA2 | NA | 12757 | ENSMUSG00000028478 | Clta | clathrin, light polypeptide (Lca) |
| Q9D2P8 | NA | 17433 | ENSMUSG00000032517 | Mobp | myelin-associated oligodendrocytic basic protein |
| Q9WV55 | NA | 30960 | ENSMUSG00000024091 | Vapa | vesicle-associated membrane protein, associated protein A |
| Q8BH80 | NA | 56491 | ENSMUSG00000054455 | Vapb | vesicle-associated membrane protein, associated protein B and C |
| Q52L78 | NA | 12955 | ENSMUSG00000032060 | Cryab | crystallin, alpha B |
| P23927 | NA | 12955 | ENSMUSG00000032060 | Cryab | crystallin, alpha B |
| P03995 | NA | 14580 | ENSMUSG00000020932 | Gfap | glial fibrillary acidic protein |
| **molecular function----mRNA binding----GO:0003729** | | | | | |
| C=44;O=2;E=0.12;R=16.21;rawP=0.0067;adjP=0.0415 | | | | | |
| Q91Z31 | NA | 56195 | ENSMUSG00000028134 | Ptbp2 | polypyrimidine tract binding protein 2 |
| Q9Z204 | NA | 15381 | ENSMUSG00000060373 | Hnrnpc | heterogeneous nuclear ribonucleoprotein C |
| **molecular function----binding----GO:0005488** | | | | | |
| C=10190;O=35;E=28.58;R=1.22;rawP=0.0206;adjP=0.1064 | | | | | |
| Q3TT81 | NA | 18521 | ENSMUSG00000056851 | Pcbp2 | poly(rC) binding protein 2 |
| Q61990 | NA | 18521 | ENSMUSG00000056851 | Pcbp2 | poly(rC) binding protein 2 |
| Q60749 | NA | 20218 | ENSMUSG00000028790 | Khdrbs1 | KH domain containing, RNA binding, signal transduction associated 1 |
| P70333 | NA | 56258 | ENSMUSG00000045427 | Hnrnph2 | heterogeneous nuclear ribonucleoprotein H2 |
| A2BDV8 | NA | 56258 | ENSMUSG00000045427 | Hnrnph2 | heterogeneous nuclear ribonucleoprotein H2 |
| O88737 | NA | 12217 | ENSMUSG00000032589 | Bsn | bassoon |
| Q3UXD6 | NA | 12217 | ENSMUSG00000032589 | Bsn | bassoon |
| P23242 | NA | 14609 | ENSMUSG00000050953 | Gja1 | gap junction protein, alpha 1 |
| Q8C4N2 | NA | 14609 | ENSMUSG00000050953 | Gja1 | gap junction protein, alpha 1 |
| Q9Z0H4 | NA | 14007 | ENSMUSG00000002107 | Cugbp2 | CUG triplet repeat, RNA binding protein 2 |
| Q564E6 | NA | 20823 | ENSMUSG00000068882 | Ssb | Sjogren syndrome antigen B |
| P32067 | NA | 20823 | ENSMUSG00000068882 | Ssb | Sjogren syndrome antigen B |
| P08226 | NA | 11816 | ENSMUSG00000002985 | Apoe | apolipoprotein E |
| Q3TXU4 | NA | 11816 | ENSMUSG00000002985 | Apoe | apolipoprotein E |
| Q91VM5 | NA | 19656 | ENSMUSG00000037070 | Rbmxrt | RNA binding motif protein, X chromosome retrogene |
| P61979 | NA | 15387 | NULL | Hnrnpk | heterogeneous nuclear ribonucleoprotein K |
| Q54A87 | NA | 66237 | ENSMUSG00000024403 | Atp6v1g2 | ATPase, H+ transporting, lysosomal V1 subunit G2 |
| Q9WTT4 | NA | 66237 | ENSMUSG00000024403 | Atp6v1g2 | ATPase, H+ transporting, lysosomal V1 subunit G2 |
| A7UQY4 | NA | 17876 | NULL | Myef2 | myelin basic protein expression factor 2, repressor |
| Q8C854 | NA | 17876 | NULL | Myef2 | myelin basic protein expression factor 2, repressor |
| O08539 | NA | 30948 | ENSMUSG00000024381 | Bin1 | bridging integrator 1 |
| Q60668 | NA | 11991 | ENSMUSG00000000568 | Hnrnpd | heterogeneous nuclear ribonucleoprotein D |
| Q3UMT7 | NA | 15388 | ENSMUSG00000015165 | Hnrnpl | heterogeneous nuclear ribonucleoprotein L |
| Q8R081 | NA | 15388 | ENSMUSG00000015165 | Hnrnpl | heterogeneous nuclear ribonucleoprotein L |
| Q9CVU5 | NA | 15388 | ENSMUSG00000015165 | Hnrnpl | heterogeneous nuclear ribonucleoprotein L |
| Q5BL18 | NA | 17184 | ENSMUSG00000037236 | Matr3 | matrin 3 |
| Q8K310 | NA | 17184 | ENSMUSG00000037236 | Matr3 | matrin 3 |
| Q544Z3 | NA | 15384 | ENSMUSG00000020358 | Hnrnpab | heterogeneous nuclear ribonucleoprotein A/B |
| Q99020 | NA | 15384 | ENSMUSG00000020358 | Hnrnpab | heterogeneous nuclear ribonucleoprotein A/B |
| O35737 | NA | 59013 | ENSMUSG00000007850 | Hnrnph1 | heterogeneous nuclear ribonucleoprotein H1 |
| Q811L7 | NA | 59013 | ENSMUSG00000007850 | Hnrnph1 | heterogeneous nuclear ribonucleoprotein H1 |
| Q61644 | NA | 23969 | ENSMUSG00000040276 | Pacsin1 | protein kinase C and casein kinase substrate in neurons 1 |
| Q543Y7 | NA | 23969 | ENSMUSG00000040276 | Pacsin1 | protein kinase C and casein kinase substrate in neurons 1 |
| Q6PFA2 | NA | 12757 | ENSMUSG00000028478 | Clta | clathrin, light polypeptide (Lca) |
| P63141 | NA | 16490 | ENSMUSG00000040724 | Kcna2 | potassium voltage-gated channel, shaker-related subfamily, member 2 |
| B2RS05 | NA | 16490 | ENSMUSG00000040724 | Kcna2 | potassium voltage-gated channel, shaker-related subfamily, member 2 |
| O88569 | NA | 53379 | ENSMUSG00000004980 | Hnrnpa2b1 | heterogeneous nuclear ribonucleoprotein A2/B1 |
| P49312 | NA | 15382 | ENSMUSG00000046434 | Hnrnpa1 | heterogeneous nuclear ribonucleoprotein A1 |
| Q3UY19 | NA | 15898 | ENSMUSG00000032174 | Icam5 | intercellular adhesion molecule 5, telencephalin |
| Q2KHL7 | NA | 15898 | ENSMUSG00000032174 | Icam5 | intercellular adhesion molecule 5, telencephalin |
| Q9DB41 | NA | 71803 | ENSMUSG00000004902 | Slc25a18 | solute carrier family 25 (mitochondrial carrier), member 18 |
| Q544R5 | NA | 230908 | ENSMUSG00000041459 | Tardbp | TAR DNA binding protein |
| Q921F2 | NA | 230908 | ENSMUSG00000041459 | Tardbp | TAR DNA binding protein |
| Q91Z31 | NA | 56195 | ENSMUSG00000028134 | Ptbp2 | polypyrimidine tract binding protein 2 |
| P03995 | NA | 14580 | ENSMUSG00000020932 | Gfap | glial fibrillary acidic protein |
| Q8VHM5 | NA | 74326 | ENSMUSG00000066037 | Hnrnpr | heterogeneous nuclear ribonucleoprotein R |
| P40240 | NA | 12527 | ENSMUSG00000030342 | Cd9 | CD9 antigen |
| Q61753 | NA | 236539 | ENSMUSG00000053398 | Phgdh | 3-phosphoglycerate dehydrogenase |
| Q8VEK3 | NA | 51810 | ENSMUSG00000039630 | Hnrnpu | heterogeneous nuclear ribonucleoprotein U |
| Q3THA6 | NA | 225027 | ENSMUSG00000024097 | Sfrs7 | splicing factor, arginine/serine-rich 7 |
| Q8BL97 | NA | 225027 | ENSMUSG00000024097 | Sfrs7 | splicing factor, arginine/serine-rich 7 |
| Q9Z204 | NA | 15381 | ENSMUSG00000060373 | Hnrnpc | heterogeneous nuclear ribonucleoprotein C |
| Q5SX50 | NA | 18643 | ENSMUSG00000018293 | Pfn1 | profilin 1 |
| Q3U7V7 | NA | 18643 | ENSMUSG00000018293 | Pfn1 | profilin 1 |
| P62962 | NA | 18643 | ENSMUSG00000018293 | Pfn1 | profilin 1 |
| **molecular function----oxidoreductase activity, acting on the CH-OH group of donors, NAD or NADP as acceptor----GO:0016616** | | | | | |
| C=103;O=2;E=0.29;R=6.92;rawP=0.0337;adjP=0.1492 | | | | | |
| Q9JII6 | NA | 58810 | ENSMUSG00000028692 | Akr1a4 | aldo-keto reductase family 1, member A4 (aldehyde reductase) |
| Q540D7 | NA | 58810 | ENSMUSG00000028692 | Akr1a4 | aldo-keto reductase family 1, member A4 (aldehyde reductase) |
| Q61753 | NA | 236539 | ENSMUSG00000053398 | Phgdh | 3-phosphoglycerate dehydrogenase |
| **molecular function----oxidoreductase activity, acting on CH-OH group of donors----GO:0016614** | | | | | |
| C=114;O=2;E=0.32;R=6.26;rawP=0.0406;adjP=0.1573 | | | | | |
| Q9JII6 | NA | 58810 | ENSMUSG00000028692 | Akr1a4 | aldo-keto reductase family 1, member A4 (aldehyde reductase) |
| Q540D7 | NA | 58810 | ENSMUSG00000028692 | Akr1a4 | aldo-keto reductase family 1, member A4 (aldehyde reductase) |
| Q61753 | NA | 236539 | ENSMUSG00000053398 | Phgdh | 3-phosphoglycerate dehydrogenase |
| **molecular function----transporter activity----GO:0005215** | | | | | |
| C=941;O=5;E=2.64;R=1.89;rawP=0.1214;adjP=0.4182 | | | | | |
| P23242 | NA | 14609 | ENSMUSG00000050953 | Gja1 | gap junction protein, alpha 1 |
| Q8C4N2 | NA | 14609 | ENSMUSG00000050953 | Gja1 | gap junction protein, alpha 1 |
| Q54A87 | NA | 66237 | ENSMUSG00000024403 | Atp6v1g2 | ATPase, H+ transporting, lysosomal V1 subunit G2 |
| Q9WTT4 | NA | 66237 | ENSMUSG00000024403 | Atp6v1g2 | ATPase, H+ transporting, lysosomal V1 subunit G2 |
| P63141 | NA | 16490 | ENSMUSG00000040724 | Kcna2 | potassium voltage-gated channel, shaker-related subfamily, member 2 |
| B2RS05 | NA | 16490 | ENSMUSG00000040724 | Kcna2 | potassium voltage-gated channel, shaker-related subfamily, member 2 |
| P08226 | NA | 11816 | ENSMUSG00000002985 | Apoe | apolipoprotein E |
| Q3TXU4 | NA | 11816 | ENSMUSG00000002985 | Apoe | apolipoprotein E |
| Q9DB41 | NA | 71803 | ENSMUSG00000004902 | Slc25a18 | solute carrier family 25 (mitochondrial carrier), member 18 |
| **molecular function----transmembrane transporter activity----GO:0022857** | | | | | |
| C=742;O=4;E=2.08;R=1.92;rawP=0.1531;adjP=0.4501 | | | | | |
| P23242 | NA | 14609 | ENSMUSG00000050953 | Gja1 | gap junction protein, alpha 1 |
| Q8C4N2 | NA | 14609 | ENSMUSG00000050953 | Gja1 | gap junction protein, alpha 1 |
| Q54A87 | NA | 66237 | ENSMUSG00000024403 | Atp6v1g2 | ATPase, H+ transporting, lysosomal V1 subunit G2 |
| Q9WTT4 | NA | 66237 | ENSMUSG00000024403 | Atp6v1g2 | ATPase, H+ transporting, lysosomal V1 subunit G2 |
| P63141 | NA | 16490 | ENSMUSG00000040724 | Kcna2 | potassium voltage-gated channel, shaker-related subfamily, member 2 |
| B2RS05 | NA | 16490 | ENSMUSG00000040724 | Kcna2 | potassium voltage-gated channel, shaker-related subfamily, member 2 |
| Q9DB41 | NA | 71803 | ENSMUSG00000004902 | Slc25a18 | solute carrier family 25 (mitochondrial carrier), member 18 |
| **cellular component----ribonucleoprotein complex----GO:0030529** | | | | | |
| C=414;O=15;E=1.08;R=13.86;rawP=5.57e-14;adjP=3.56e-12 | | | | | |
| Q60668 | NA | 11991 | ENSMUSG00000000568 | Hnrnpd | heterogeneous nuclear ribonucleoprotein D |
| Q564E6 | NA | 20823 | ENSMUSG00000068882 | Ssb | Sjogren syndrome antigen B |
| P32067 | NA | 20823 | ENSMUSG00000068882 | Ssb | Sjogren syndrome antigen B |
| Q3TT81 | NA | 18521 | ENSMUSG00000056851 | Pcbp2 | poly(rC) binding protein 2 |
| Q61990 | NA | 18521 | ENSMUSG00000056851 | Pcbp2 | poly(rC) binding protein 2 |
| Q91Z31 | NA | 56195 | ENSMUSG00000028134 | Ptbp2 | polypyrimidine tract binding protein 2 |
| Q3UMT7 | NA | 15388 | ENSMUSG00000015165 | Hnrnpl | heterogeneous nuclear ribonucleoprotein L |
| Q8R081 | NA | 15388 | ENSMUSG00000015165 | Hnrnpl | heterogeneous nuclear ribonucleoprotein L |
| Q9CVU5 | NA | 15388 | ENSMUSG00000015165 | Hnrnpl | heterogeneous nuclear ribonucleoprotein L |
| Q91VM5 | NA | 19656 | ENSMUSG00000037070 | Rbmxrt | RNA binding motif protein, X chromosome retrogene |
| Q8VHM5 | NA | 74326 | ENSMUSG00000066037 | Hnrnpr | heterogeneous nuclear ribonucleoprotein R |
| P61979 | NA | 15387 | NULL | Hnrnpk | heterogeneous nuclear ribonucleoprotein K |
| Q544Z3 | NA | 15384 | ENSMUSG00000020358 | Hnrnpab | heterogeneous nuclear ribonucleoprotein A/B |
| Q99020 | NA | 15384 | ENSMUSG00000020358 | Hnrnpab | heterogeneous nuclear ribonucleoprotein A/B |
| O35737 | NA | 59013 | ENSMUSG00000007850 | Hnrnph1 | heterogeneous nuclear ribonucleoprotein H1 |
| Q811L7 | NA | 59013 | ENSMUSG00000007850 | Hnrnph1 | heterogeneous nuclear ribonucleoprotein H1 |
| O88569 | NA | 53379 | ENSMUSG00000004980 | Hnrnpa2b1 | heterogeneous nuclear ribonucleoprotein A2/B1 |
| Q8VEK3 | NA | 51810 | ENSMUSG00000039630 | Hnrnpu | heterogeneous nuclear ribonucleoprotein U |
| P49312 | NA | 15382 | ENSMUSG00000046434 | Hnrnpa1 | heterogeneous nuclear ribonucleoprotein A1 |
| Q9Z204 | NA | 15381 | ENSMUSG00000060373 | Hnrnpc | heterogeneous nuclear ribonucleoprotein C |
| P70333 | NA | 56258 | ENSMUSG00000045427 | Hnrnph2 | heterogeneous nuclear ribonucleoprotein H2 |
| A2BDV8 | NA | 56258 | ENSMUSG00000045427 | Hnrnph2 | heterogeneous nuclear ribonucleoprotein H2 |
| **cellular component----macromolecular complex----GO:0032991** | | | | | |
| C=2161;O=22;E=5.65;R=3.89;rawP=1.82e-09;adjP=5.82e-08 | | | | | |
| Q60668 | NA | 11991 | ENSMUSG00000000568 | Hnrnpd | heterogeneous nuclear ribonucleoprotein D |
| Q3TT81 | NA | 18521 | ENSMUSG00000056851 | Pcbp2 | poly(rC) binding protein 2 |
| Q61990 | NA | 18521 | ENSMUSG00000056851 | Pcbp2 | poly(rC) binding protein 2 |
| Q9WV55 | NA | 30960 | ENSMUSG00000024091 | Vapa | vesicle-associated membrane protein, associated protein A |
| Q3UMT7 | NA | 15388 | ENSMUSG00000015165 | Hnrnpl | heterogeneous nuclear ribonucleoprotein L |
| Q8R081 | NA | 15388 | ENSMUSG00000015165 | Hnrnpl | heterogeneous nuclear ribonucleoprotein L |
| Q9CVU5 | NA | 15388 | ENSMUSG00000015165 | Hnrnpl | heterogeneous nuclear ribonucleoprotein L |
| Q544Z3 | NA | 15384 | ENSMUSG00000020358 | Hnrnpab | heterogeneous nuclear ribonucleoprotein A/B |
| Q99020 | NA | 15384 | ENSMUSG00000020358 | Hnrnpab | heterogeneous nuclear ribonucleoprotein A/B |
| Q6PFA2 | NA | 12757 | ENSMUSG00000028478 | Clta | clathrin, light polypeptide (Lca) |
| O35737 | NA | 59013 | ENSMUSG00000007850 | Hnrnph1 | heterogeneous nuclear ribonucleoprotein H1 |
| Q811L7 | NA | 59013 | ENSMUSG00000007850 | Hnrnph1 | heterogeneous nuclear ribonucleoprotein H1 |
| P63141 | NA | 16490 | ENSMUSG00000040724 | Kcna2 | potassium voltage-gated channel, shaker-related subfamily, member 2 |
| B2RS05 | NA | 16490 | ENSMUSG00000040724 | Kcna2 | potassium voltage-gated channel, shaker-related subfamily, member 2 |
| O88569 | NA | 53379 | ENSMUSG00000004980 | Hnrnpa2b1 | heterogeneous nuclear ribonucleoprotein A2/B1 |
| P49312 | NA | 15382 | ENSMUSG00000046434 | Hnrnpa1 | heterogeneous nuclear ribonucleoprotein A1 |
| P70333 | NA | 56258 | ENSMUSG00000045427 | Hnrnph2 | heterogeneous nuclear ribonucleoprotein H2 |
| A2BDV8 | NA | 56258 | ENSMUSG00000045427 | Hnrnph2 | heterogeneous nuclear ribonucleoprotein H2 |
| P23242 | NA | 14609 | ENSMUSG00000050953 | Gja1 | gap junction protein, alpha 1 |
| Q8C4N2 | NA | 14609 | ENSMUSG00000050953 | Gja1 | gap junction protein, alpha 1 |
| Q564E6 | NA | 20823 | ENSMUSG00000068882 | Ssb | Sjogren syndrome antigen B |
| P32067 | NA | 20823 | ENSMUSG00000068882 | Ssb | Sjogren syndrome antigen B |
| Q91Z31 | NA | 56195 | ENSMUSG00000028134 | Ptbp2 | polypyrimidine tract binding protein 2 |
| P08226 | NA | 11816 | ENSMUSG00000002985 | Apoe | apolipoprotein E |
| Q3TXU4 | NA | 11816 | ENSMUSG00000002985 | Apoe | apolipoprotein E |
| Q91VM5 | NA | 19656 | ENSMUSG00000037070 | Rbmxrt | RNA binding motif protein, X chromosome retrogene |
| P03995 | NA | 14580 | ENSMUSG00000020932 | Gfap | glial fibrillary acidic protein |
| Q8VHM5 | NA | 74326 | ENSMUSG00000066037 | Hnrnpr | heterogeneous nuclear ribonucleoprotein R |
| P61979 | NA | 15387 | NULL | Hnrnpk | heterogeneous nuclear ribonucleoprotein K |
| Q54A87 | NA | 66237 | ENSMUSG00000024403 | Atp6v1g2 | ATPase, H+ transporting, lysosomal V1 subunit G2 |
| Q9WTT4 | NA | 66237 | ENSMUSG00000024403 | Atp6v1g2 | ATPase, H+ transporting, lysosomal V1 subunit G2 |
| Q8VEK3 | NA | 51810 | ENSMUSG00000039630 | Hnrnpu | heterogeneous nuclear ribonucleoprotein U |
| Q9Z204 | NA | 15381 | ENSMUSG00000060373 | Hnrnpc | heterogeneous nuclear ribonucleoprotein C |
| **cellular component----spliceosomal complex----GO:0005681** | | | | | |
| C=114;O=7;E=0.30;R=23.48;rawP=1.64e-08;adjP=3.50e-07 | | | | | |
| P61979 | NA | 15387 | NULL | Hnrnpk | heterogeneous nuclear ribonucleoprotein K |
| O35737 | NA | 59013 | ENSMUSG00000007850 | Hnrnph1 | heterogeneous nuclear ribonucleoprotein H1 |
| Q811L7 | NA | 59013 | ENSMUSG00000007850 | Hnrnph1 | heterogeneous nuclear ribonucleoprotein H1 |
| O88569 | NA | 53379 | ENSMUSG00000004980 | Hnrnpa2b1 | heterogeneous nuclear ribonucleoprotein A2/B1 |
| Q91Z31 | NA | 56195 | ENSMUSG00000028134 | Ptbp2 | polypyrimidine tract binding protein 2 |
| P49312 | NA | 15382 | ENSMUSG00000046434 | Hnrnpa1 | heterogeneous nuclear ribonucleoprotein A1 |
| Q9Z204 | NA | 15381 | ENSMUSG00000060373 | Hnrnpc | heterogeneous nuclear ribonucleoprotein C |
| Q91VM5 | NA | 19656 | ENSMUSG00000037070 | Rbmxrt | RNA binding motif protein, X chromosome retrogene |
| **cellular component----intracellular part----GO:0044424** | | | | | |
| C=9057;O=36;E=23.68;R=1.52;rawP=3.15e-05;adjP=0.0005 | | | | | |
| Q3TT81 | NA | 18521 | ENSMUSG00000056851 | Pcbp2 | poly(rC) binding protein 2 |
| Q61990 | NA | 18521 | ENSMUSG00000056851 | Pcbp2 | poly(rC) binding protein 2 |
| Q9WV55 | NA | 30960 | ENSMUSG00000024091 | Vapa | vesicle-associated membrane protein, associated protein A |
| Q8BWT1 | NA | 52538 | ENSMUSG00000036880 | Acaa2 | acetyl-Coenzyme A acyltransferase 2 (mitochondrial 3-oxoacyl-Coenzyme A thiolase) |
| Q60749 | NA | 20218 | ENSMUSG00000028790 | Khdrbs1 | KH domain containing, RNA binding, signal transduction associated 1 |
| P70333 | NA | 56258 | ENSMUSG00000045427 | Hnrnph2 | heterogeneous nuclear ribonucleoprotein H2 |
| A2BDV8 | NA | 56258 | ENSMUSG00000045427 | Hnrnph2 | heterogeneous nuclear ribonucleoprotein H2 |
| O88737 | NA | 12217 | ENSMUSG00000032589 | Bsn | bassoon |
| Q3UXD6 | NA | 12217 | ENSMUSG00000032589 | Bsn | bassoon |
| P23242 | NA | 14609 | ENSMUSG00000050953 | Gja1 | gap junction protein, alpha 1 |
| Q8C4N2 | NA | 14609 | ENSMUSG00000050953 | Gja1 | gap junction protein, alpha 1 |
| Q9Z0H4 | NA | 14007 | ENSMUSG00000002107 | Cugbp2 | CUG triplet repeat, RNA binding protein 2 |
| Q9D2P8 | NA | 17433 | ENSMUSG00000032517 | Mobp | myelin-associated oligodendrocytic basic protein |
| Q564E6 | NA | 20823 | ENSMUSG00000068882 | Ssb | Sjogren syndrome antigen B |
| P32067 | NA | 20823 | ENSMUSG00000068882 | Ssb | Sjogren syndrome antigen B |
| Q91VM5 | NA | 19656 | ENSMUSG00000037070 | Rbmxrt | RNA binding motif protein, X chromosome retrogene |
| P61979 | NA | 15387 | NULL | Hnrnpk | heterogeneous nuclear ribonucleoprotein K |
| Q54A87 | NA | 66237 | ENSMUSG00000024403 | Atp6v1g2 | ATPase, H+ transporting, lysosomal V1 subunit G2 |
| Q9WTT4 | NA | 66237 | ENSMUSG00000024403 | Atp6v1g2 | ATPase, H+ transporting, lysosomal V1 subunit G2 |
| A7UQY4 | NA | 17876 | NULL | Myef2 | myelin basic protein expression factor 2, repressor |
| Q8C854 | NA | 17876 | NULL | Myef2 | myelin basic protein expression factor 2, repressor |
| O08539 | NA | 30948 | ENSMUSG00000024381 | Bin1 | bridging integrator 1 |
| Q60668 | NA | 11991 | ENSMUSG00000000568 | Hnrnpd | heterogeneous nuclear ribonucleoprotein D |
| Q3UMT7 | NA | 15388 | ENSMUSG00000015165 | Hnrnpl | heterogeneous nuclear ribonucleoprotein L |
| Q8R081 | NA | 15388 | ENSMUSG00000015165 | Hnrnpl | heterogeneous nuclear ribonucleoprotein L |
| Q9CVU5 | NA | 15388 | ENSMUSG00000015165 | Hnrnpl | heterogeneous nuclear ribonucleoprotein L |
| Q5BL18 | NA | 17184 | ENSMUSG00000037236 | Matr3 | matrin 3 |
| Q8K310 | NA | 17184 | ENSMUSG00000037236 | Matr3 | matrin 3 |
| Q544Z3 | NA | 15384 | ENSMUSG00000020358 | Hnrnpab | heterogeneous nuclear ribonucleoprotein A/B |
| Q99020 | NA | 15384 | ENSMUSG00000020358 | Hnrnpab | heterogeneous nuclear ribonucleoprotein A/B |
| O35737 | NA | 59013 | ENSMUSG00000007850 | Hnrnph1 | heterogeneous nuclear ribonucleoprotein H1 |
| Q811L7 | NA | 59013 | ENSMUSG00000007850 | Hnrnph1 | heterogeneous nuclear ribonucleoprotein H1 |
| Q61644 | NA | 23969 | ENSMUSG00000040276 | Pacsin1 | protein kinase C and casein kinase substrate in neurons 1 |
| Q543Y7 | NA | 23969 | ENSMUSG00000040276 | Pacsin1 | protein kinase C and casein kinase substrate in neurons 1 |
| Q6PFA2 | NA | 12757 | ENSMUSG00000028478 | Clta | clathrin, light polypeptide (Lca) |
| O88569 | NA | 53379 | ENSMUSG00000004980 | Hnrnpa2b1 | heterogeneous nuclear ribonucleoprotein A2/B1 |
| P49312 | NA | 15382 | ENSMUSG00000046434 | Hnrnpa1 | heterogeneous nuclear ribonucleoprotein A1 |
| Q9DB41 | NA | 71803 | ENSMUSG00000004902 | Slc25a18 | solute carrier family 25 (mitochondrial carrier), member 18 |
| Q544R5 | NA | 230908 | ENSMUSG00000041459 | Tardbp | TAR DNA binding protein |
| Q921F2 | NA | 230908 | ENSMUSG00000041459 | Tardbp | TAR DNA binding protein |
| Q91Z31 | NA | 56195 | ENSMUSG00000028134 | Ptbp2 | polypyrimidine tract binding protein 2 |
| Q52L78 | NA | 12955 | ENSMUSG00000032060 | Cryab | crystallin, alpha B |
| P23927 | NA | 12955 | ENSMUSG00000032060 | Cryab | crystallin, alpha B |
| P03995 | NA | 14580 | ENSMUSG00000020932 | Gfap | glial fibrillary acidic protein |
| Q6GT24 | NA | 11758 | ENSMUSG00000026701 | Prdx6 | peroxiredoxin 6 |
| O08709 | NA | 11758 | ENSMUSG00000026701 | Prdx6 | peroxiredoxin 6 |
| Q8VHM5 | NA | 74326 | ENSMUSG00000066037 | Hnrnpr | heterogeneous nuclear ribonucleoprotein R |
| Q9JII6 | NA | 58810 | ENSMUSG00000028692 | Akr1a4 | aldo-keto reductase family 1, member A4 (aldehyde reductase) |
| Q540D7 | NA | 58810 | ENSMUSG00000028692 | Akr1a4 | aldo-keto reductase family 1, member A4 (aldehyde reductase) |
| Q8VEK3 | NA | 51810 | ENSMUSG00000039630 | Hnrnpu | heterogeneous nuclear ribonucleoprotein U |
| Q3THA6 | NA | 225027 | ENSMUSG00000024097 | Sfrs7 | splicing factor, arginine/serine-rich 7 |
| Q8BL97 | NA | 225027 | ENSMUSG00000024097 | Sfrs7 | splicing factor, arginine/serine-rich 7 |
| Q9Z204 | NA | 15381 | ENSMUSG00000060373 | Hnrnpc | heterogeneous nuclear ribonucleoprotein C |
| Q5SX50 | NA | 18643 | ENSMUSG00000018293 | Pfn1 | profilin 1 |
| Q3U7V7 | NA | 18643 | ENSMUSG00000018293 | Pfn1 | profilin 1 |
| P62962 | NA | 18643 | ENSMUSG00000018293 | Pfn1 | profilin 1 |
| **cellular component----intracellular membrane-bounded organelle----GO:0043231** | | | | | |
| C=6827;O=30;E=17.85;R=1.68;rawP=0.0001;adjP=0.0006 | | | | | |
| Q60668 | NA | 11991 | ENSMUSG00000000568 | Hnrnpd | heterogeneous nuclear ribonucleoprotein D |
| O08539 | NA | 30948 | ENSMUSG00000024381 | Bin1 | bridging integrator 1 |
| Q3TT81 | NA | 18521 | ENSMUSG00000056851 | Pcbp2 | poly(rC) binding protein 2 |
| Q61990 | NA | 18521 | ENSMUSG00000056851 | Pcbp2 | poly(rC) binding protein 2 |
| Q9WV55 | NA | 30960 | ENSMUSG00000024091 | Vapa | vesicle-associated membrane protein, associated protein A |
| Q5BL18 | NA | 17184 | ENSMUSG00000037236 | Matr3 | matrin 3 |
| Q8K310 | NA | 17184 | ENSMUSG00000037236 | Matr3 | matrin 3 |
| Q3UMT7 | NA | 15388 | ENSMUSG00000015165 | Hnrnpl | heterogeneous nuclear ribonucleoprotein L |
| Q8R081 | NA | 15388 | ENSMUSG00000015165 | Hnrnpl | heterogeneous nuclear ribonucleoprotein L |
| Q9CVU5 | NA | 15388 | ENSMUSG00000015165 | Hnrnpl | heterogeneous nuclear ribonucleoprotein L |
| Q544Z3 | NA | 15384 | ENSMUSG00000020358 | Hnrnpab | heterogeneous nuclear ribonucleoprotein A/B |
| Q99020 | NA | 15384 | ENSMUSG00000020358 | Hnrnpab | heterogeneous nuclear ribonucleoprotein A/B |
| Q6PFA2 | NA | 12757 | ENSMUSG00000028478 | Clta | clathrin, light polypeptide (Lca) |
| Q61644 | NA | 23969 | ENSMUSG00000040276 | Pacsin1 | protein kinase C and casein kinase substrate in neurons 1 |
| Q543Y7 | NA | 23969 | ENSMUSG00000040276 | Pacsin1 | protein kinase C and casein kinase substrate in neurons 1 |
| O35737 | NA | 59013 | ENSMUSG00000007850 | Hnrnph1 | heterogeneous nuclear ribonucleoprotein H1 |
| Q811L7 | NA | 59013 | ENSMUSG00000007850 | Hnrnph1 | heterogeneous nuclear ribonucleoprotein H1 |
| O88569 | NA | 53379 | ENSMUSG00000004980 | Hnrnpa2b1 | heterogeneous nuclear ribonucleoprotein A2/B1 |
| Q8BWT1 | NA | 52538 | ENSMUSG00000036880 | Acaa2 | acetyl-Coenzyme A acyltransferase 2 (mitochondrial 3-oxoacyl-Coenzyme A thiolase) |
| P49312 | NA | 15382 | ENSMUSG00000046434 | Hnrnpa1 | heterogeneous nuclear ribonucleoprotein A1 |
| Q60749 | NA | 20218 | ENSMUSG00000028790 | Khdrbs1 | KH domain containing, RNA binding, signal transduction associated 1 |
| P70333 | NA | 56258 | ENSMUSG00000045427 | Hnrnph2 | heterogeneous nuclear ribonucleoprotein H2 |
| A2BDV8 | NA | 56258 | ENSMUSG00000045427 | Hnrnph2 | heterogeneous nuclear ribonucleoprotein H2 |
| Q9DB41 | NA | 71803 | ENSMUSG00000004902 | Slc25a18 | solute carrier family 25 (mitochondrial carrier), member 18 |
| Q544R5 | NA | 230908 | ENSMUSG00000041459 | Tardbp | TAR DNA binding protein |
| Q921F2 | NA | 230908 | ENSMUSG00000041459 | Tardbp | TAR DNA binding protein |
| Q9Z0H4 | NA | 14007 | ENSMUSG00000002107 | Cugbp2 | CUG triplet repeat, RNA binding protein 2 |
| Q9D2P8 | NA | 17433 | ENSMUSG00000032517 | Mobp | myelin-associated oligodendrocytic basic protein |
| Q564E6 | NA | 20823 | ENSMUSG00000068882 | Ssb | Sjogren syndrome antigen B |
| P32067 | NA | 20823 | ENSMUSG00000068882 | Ssb | Sjogren syndrome antigen B |
| Q91Z31 | NA | 56195 | ENSMUSG00000028134 | Ptbp2 | polypyrimidine tract binding protein 2 |
| Q6GT24 | NA | 11758 | ENSMUSG00000026701 | Prdx6 | peroxiredoxin 6 |
| O08709 | NA | 11758 | ENSMUSG00000026701 | Prdx6 | peroxiredoxin 6 |
| Q91VM5 | NA | 19656 | ENSMUSG00000037070 | Rbmxrt | RNA binding motif protein, X chromosome retrogene |
| Q8VHM5 | NA | 74326 | ENSMUSG00000066037 | Hnrnpr | heterogeneous nuclear ribonucleoprotein R |
| P61979 | NA | 15387 | NULL | Hnrnpk | heterogeneous nuclear ribonucleoprotein K |
| Q54A87 | NA | 66237 | ENSMUSG00000024403 | Atp6v1g2 | ATPase, H+ transporting, lysosomal V1 subunit G2 |
| Q9WTT4 | NA | 66237 | ENSMUSG00000024403 | Atp6v1g2 | ATPase, H+ transporting, lysosomal V1 subunit G2 |
| Q3THA6 | NA | 225027 | ENSMUSG00000024097 | Sfrs7 | splicing factor, arginine/serine-rich 7 |
| Q8BL97 | NA | 225027 | ENSMUSG00000024097 | Sfrs7 | splicing factor, arginine/serine-rich 7 |
| Q9Z204 | NA | 15381 | ENSMUSG00000060373 | Hnrnpc | heterogeneous nuclear ribonucleoprotein C |
| Q5SX50 | NA | 18643 | ENSMUSG00000018293 | Pfn1 | profilin 1 |
| Q3U7V7 | NA | 18643 | ENSMUSG00000018293 | Pfn1 | profilin 1 |
| P62962 | NA | 18643 | ENSMUSG00000018293 | Pfn1 | profilin 1 |
| A7UQY4 | NA | 17876 | NULL | Myef2 | myelin basic protein expression factor 2, repressor |
| Q8C854 | NA | 17876 | NULL | Myef2 | myelin basic protein expression factor 2, repressor |
| **cellular component----nucleus----GO:0005634** | | | | | |
| C=4046;O=22;E=10.58;R=2.08;rawP=0.0001;adjP=0.0006 | | | | | |
| Q60668 | NA | 11991 | ENSMUSG00000000568 | Hnrnpd | heterogeneous nuclear ribonucleoprotein D |
| O08539 | NA | 30948 | ENSMUSG00000024381 | Bin1 | bridging integrator 1 |
| Q3TT81 | NA | 18521 | ENSMUSG00000056851 | Pcbp2 | poly(rC) binding protein 2 |
| Q61990 | NA | 18521 | ENSMUSG00000056851 | Pcbp2 | poly(rC) binding protein 2 |
| Q5BL18 | NA | 17184 | ENSMUSG00000037236 | Matr3 | matrin 3 |
| Q8K310 | NA | 17184 | ENSMUSG00000037236 | Matr3 | matrin 3 |
| Q3UMT7 | NA | 15388 | ENSMUSG00000015165 | Hnrnpl | heterogeneous nuclear ribonucleoprotein L |
| Q8R081 | NA | 15388 | ENSMUSG00000015165 | Hnrnpl | heterogeneous nuclear ribonucleoprotein L |
| Q9CVU5 | NA | 15388 | ENSMUSG00000015165 | Hnrnpl | heterogeneous nuclear ribonucleoprotein L |
| Q544Z3 | NA | 15384 | ENSMUSG00000020358 | Hnrnpab | heterogeneous nuclear ribonucleoprotein A/B |
| Q99020 | NA | 15384 | ENSMUSG00000020358 | Hnrnpab | heterogeneous nuclear ribonucleoprotein A/B |
| O35737 | NA | 59013 | ENSMUSG00000007850 | Hnrnph1 | heterogeneous nuclear ribonucleoprotein H1 |
| Q811L7 | NA | 59013 | ENSMUSG00000007850 | Hnrnph1 | heterogeneous nuclear ribonucleoprotein H1 |
| O88569 | NA | 53379 | ENSMUSG00000004980 | Hnrnpa2b1 | heterogeneous nuclear ribonucleoprotein A2/B1 |
| P49312 | NA | 15382 | ENSMUSG00000046434 | Hnrnpa1 | heterogeneous nuclear ribonucleoprotein A1 |
| Q60749 | NA | 20218 | ENSMUSG00000028790 | Khdrbs1 | KH domain containing, RNA binding, signal transduction associated 1 |
| P70333 | NA | 56258 | ENSMUSG00000045427 | Hnrnph2 | heterogeneous nuclear ribonucleoprotein H2 |
| A2BDV8 | NA | 56258 | ENSMUSG00000045427 | Hnrnph2 | heterogeneous nuclear ribonucleoprotein H2 |
| Q9Z0H4 | NA | 14007 | ENSMUSG00000002107 | Cugbp2 | CUG triplet repeat, RNA binding protein 2 |
| Q544R5 | NA | 230908 | ENSMUSG00000041459 | Tardbp | TAR DNA binding protein |
| Q921F2 | NA | 230908 | ENSMUSG00000041459 | Tardbp | TAR DNA binding protein |
| Q564E6 | NA | 20823 | ENSMUSG00000068882 | Ssb | Sjogren syndrome antigen B |
| P32067 | NA | 20823 | ENSMUSG00000068882 | Ssb | Sjogren syndrome antigen B |
| Q91Z31 | NA | 56195 | ENSMUSG00000028134 | Ptbp2 | polypyrimidine tract binding protein 2 |
| Q91VM5 | NA | 19656 | ENSMUSG00000037070 | Rbmxrt | RNA binding motif protein, X chromosome retrogene |
| Q8VHM5 | NA | 74326 | ENSMUSG00000066037 | Hnrnpr | heterogeneous nuclear ribonucleoprotein R |
| P61979 | NA | 15387 | NULL | Hnrnpk | heterogeneous nuclear ribonucleoprotein K |
| Q3THA6 | NA | 225027 | ENSMUSG00000024097 | Sfrs7 | splicing factor, arginine/serine-rich 7 |
| Q8BL97 | NA | 225027 | ENSMUSG00000024097 | Sfrs7 | splicing factor, arginine/serine-rich 7 |
| Q9Z204 | NA | 15381 | ENSMUSG00000060373 | Hnrnpc | heterogeneous nuclear ribonucleoprotein C |
| Q5SX50 | NA | 18643 | ENSMUSG00000018293 | Pfn1 | profilin 1 |
| Q3U7V7 | NA | 18643 | ENSMUSG00000018293 | Pfn1 | profilin 1 |
| P62962 | NA | 18643 | ENSMUSG00000018293 | Pfn1 | profilin 1 |
| A7UQY4 | NA | 17876 | NULL | Myef2 | myelin basic protein expression factor 2, repressor |
| Q8C854 | NA | 17876 | NULL | Myef2 | myelin basic protein expression factor 2, repressor |
| **cellular component----intracellular organelle----GO:0043229** | | | | | |
| C=7707;O=32;E=20.15;R=1.59;rawP=0.0001;adjP=0.0006 | | | | | |
| Q3TT81 | NA | 18521 | ENSMUSG00000056851 | Pcbp2 | poly(rC) binding protein 2 |
| Q61990 | NA | 18521 | ENSMUSG00000056851 | Pcbp2 | poly(rC) binding protein 2 |
| Q9WV55 | NA | 30960 | ENSMUSG00000024091 | Vapa | vesicle-associated membrane protein, associated protein A |
| Q8BWT1 | NA | 52538 | ENSMUSG00000036880 | Acaa2 | acetyl-Coenzyme A acyltransferase 2 (mitochondrial 3-oxoacyl-Coenzyme A thiolase) |
| Q60749 | NA | 20218 | ENSMUSG00000028790 | Khdrbs1 | KH domain containing, RNA binding, signal transduction associated 1 |
| P70333 | NA | 56258 | ENSMUSG00000045427 | Hnrnph2 | heterogeneous nuclear ribonucleoprotein H2 |
| A2BDV8 | NA | 56258 | ENSMUSG00000045427 | Hnrnph2 | heterogeneous nuclear ribonucleoprotein H2 |
| O88737 | NA | 12217 | ENSMUSG00000032589 | Bsn | bassoon |
| Q3UXD6 | NA | 12217 | ENSMUSG00000032589 | Bsn | bassoon |
| Q9Z0H4 | NA | 14007 | ENSMUSG00000002107 | Cugbp2 | CUG triplet repeat, RNA binding protein 2 |
| Q9D2P8 | NA | 17433 | ENSMUSG00000032517 | Mobp | myelin-associated oligodendrocytic basic protein |
| Q564E6 | NA | 20823 | ENSMUSG00000068882 | Ssb | Sjogren syndrome antigen B |
| P32067 | NA | 20823 | ENSMUSG00000068882 | Ssb | Sjogren syndrome antigen B |
| Q91VM5 | NA | 19656 | ENSMUSG00000037070 | Rbmxrt | RNA binding motif protein, X chromosome retrogene |
| P61979 | NA | 15387 | NULL | Hnrnpk | heterogeneous nuclear ribonucleoprotein K |
| Q54A87 | NA | 66237 | ENSMUSG00000024403 | Atp6v1g2 | ATPase, H+ transporting, lysosomal V1 subunit G2 |
| Q9WTT4 | NA | 66237 | ENSMUSG00000024403 | Atp6v1g2 | ATPase, H+ transporting, lysosomal V1 subunit G2 |
| A7UQY4 | NA | 17876 | NULL | Myef2 | myelin basic protein expression factor 2, repressor |
| Q8C854 | NA | 17876 | NULL | Myef2 | myelin basic protein expression factor 2, repressor |
| O08539 | NA | 30948 | ENSMUSG00000024381 | Bin1 | bridging integrator 1 |
| Q60668 | NA | 11991 | ENSMUSG00000000568 | Hnrnpd | heterogeneous nuclear ribonucleoprotein D |
| Q3UMT7 | NA | 15388 | ENSMUSG00000015165 | Hnrnpl | heterogeneous nuclear ribonucleoprotein L |
| Q8R081 | NA | 15388 | ENSMUSG00000015165 | Hnrnpl | heterogeneous nuclear ribonucleoprotein L |
| Q9CVU5 | NA | 15388 | ENSMUSG00000015165 | Hnrnpl | heterogeneous nuclear ribonucleoprotein L |
| Q5BL18 | NA | 17184 | ENSMUSG00000037236 | Matr3 | matrin 3 |
| Q8K310 | NA | 17184 | ENSMUSG00000037236 | Matr3 | matrin 3 |
| Q544Z3 | NA | 15384 | ENSMUSG00000020358 | Hnrnpab | heterogeneous nuclear ribonucleoprotein A/B |
| Q99020 | NA | 15384 | ENSMUSG00000020358 | Hnrnpab | heterogeneous nuclear ribonucleoprotein A/B |
| O35737 | NA | 59013 | ENSMUSG00000007850 | Hnrnph1 | heterogeneous nuclear ribonucleoprotein H1 |
| Q811L7 | NA | 59013 | ENSMUSG00000007850 | Hnrnph1 | heterogeneous nuclear ribonucleoprotein H1 |
| Q61644 | NA | 23969 | ENSMUSG00000040276 | Pacsin1 | protein kinase C and casein kinase substrate in neurons 1 |
| Q543Y7 | NA | 23969 | ENSMUSG00000040276 | Pacsin1 | protein kinase C and casein kinase substrate in neurons 1 |
| Q6PFA2 | NA | 12757 | ENSMUSG00000028478 | Clta | clathrin, light polypeptide (Lca) |
| O88569 | NA | 53379 | ENSMUSG00000004980 | Hnrnpa2b1 | heterogeneous nuclear ribonucleoprotein A2/B1 |
| P49312 | NA | 15382 | ENSMUSG00000046434 | Hnrnpa1 | heterogeneous nuclear ribonucleoprotein A1 |
| Q9DB41 | NA | 71803 | ENSMUSG00000004902 | Slc25a18 | solute carrier family 25 (mitochondrial carrier), member 18 |
| Q544R5 | NA | 230908 | ENSMUSG00000041459 | Tardbp | TAR DNA binding protein |
| Q921F2 | NA | 230908 | ENSMUSG00000041459 | Tardbp | TAR DNA binding protein |
| Q91Z31 | NA | 56195 | ENSMUSG00000028134 | Ptbp2 | polypyrimidine tract binding protein 2 |
| P03995 | NA | 14580 | ENSMUSG00000020932 | Gfap | glial fibrillary acidic protein |
| Q6GT24 | NA | 11758 | ENSMUSG00000026701 | Prdx6 | peroxiredoxin 6 |
| O08709 | NA | 11758 | ENSMUSG00000026701 | Prdx6 | peroxiredoxin 6 |
| Q8VHM5 | NA | 74326 | ENSMUSG00000066037 | Hnrnpr | heterogeneous nuclear ribonucleoprotein R |
| Q3THA6 | NA | 225027 | ENSMUSG00000024097 | Sfrs7 | splicing factor, arginine/serine-rich 7 |
| Q8BL97 | NA | 225027 | ENSMUSG00000024097 | Sfrs7 | splicing factor, arginine/serine-rich 7 |
| Q9Z204 | NA | 15381 | ENSMUSG00000060373 | Hnrnpc | heterogeneous nuclear ribonucleoprotein C |
| Q5SX50 | NA | 18643 | ENSMUSG00000018293 | Pfn1 | profilin 1 |
| Q3U7V7 | NA | 18643 | ENSMUSG00000018293 | Pfn1 | profilin 1 |
| P62962 | NA | 18643 | ENSMUSG00000018293 | Pfn1 | profilin 1 |
| **cellular component----organelle----GO:0043226** | | | | | |
| C=7711;O=32;E=20.16;R=1.59;rawP=0.0001;adjP=0.0006 | | | | | |
| Q3TT81 | NA | 18521 | ENSMUSG00000056851 | Pcbp2 | poly(rC) binding protein 2 |
| Q61990 | NA | 18521 | ENSMUSG00000056851 | Pcbp2 | poly(rC) binding protein 2 |
| Q9WV55 | NA | 30960 | ENSMUSG00000024091 | Vapa | vesicle-associated membrane protein, associated protein A |
| Q8BWT1 | NA | 52538 | ENSMUSG00000036880 | Acaa2 | acetyl-Coenzyme A acyltransferase 2 (mitochondrial 3-oxoacyl-Coenzyme A thiolase) |
| Q60749 | NA | 20218 | ENSMUSG00000028790 | Khdrbs1 | KH domain containing, RNA binding, signal transduction associated 1 |
| P70333 | NA | 56258 | ENSMUSG00000045427 | Hnrnph2 | heterogeneous nuclear ribonucleoprotein H2 |
| A2BDV8 | NA | 56258 | ENSMUSG00000045427 | Hnrnph2 | heterogeneous nuclear ribonucleoprotein H2 |
| O88737 | NA | 12217 | ENSMUSG00000032589 | Bsn | bassoon |
| Q3UXD6 | NA | 12217 | ENSMUSG00000032589 | Bsn | bassoon |
| Q9Z0H4 | NA | 14007 | ENSMUSG00000002107 | Cugbp2 | CUG triplet repeat, RNA binding protein 2 |
| Q9D2P8 | NA | 17433 | ENSMUSG00000032517 | Mobp | myelin-associated oligodendrocytic basic protein |
| Q564E6 | NA | 20823 | ENSMUSG00000068882 | Ssb | Sjogren syndrome antigen B |
| P32067 | NA | 20823 | ENSMUSG00000068882 | Ssb | Sjogren syndrome antigen B |
| Q91VM5 | NA | 19656 | ENSMUSG00000037070 | Rbmxrt | RNA binding motif protein, X chromosome retrogene |
| P61979 | NA | 15387 | NULL | Hnrnpk | heterogeneous nuclear ribonucleoprotein K |
| Q54A87 | NA | 66237 | ENSMUSG00000024403 | Atp6v1g2 | ATPase, H+ transporting, lysosomal V1 subunit G2 |
| Q9WTT4 | NA | 66237 | ENSMUSG00000024403 | Atp6v1g2 | ATPase, H+ transporting, lysosomal V1 subunit G2 |
| A7UQY4 | NA | 17876 | NULL | Myef2 | myelin basic protein expression factor 2, repressor |
| Q8C854 | NA | 17876 | NULL | Myef2 | myelin basic protein expression factor 2, repressor |
| O08539 | NA | 30948 | ENSMUSG00000024381 | Bin1 | bridging integrator 1 |
| Q60668 | NA | 11991 | ENSMUSG00000000568 | Hnrnpd | heterogeneous nuclear ribonucleoprotein D |
| Q3UMT7 | NA | 15388 | ENSMUSG00000015165 | Hnrnpl | heterogeneous nuclear ribonucleoprotein L |
| Q8R081 | NA | 15388 | ENSMUSG00000015165 | Hnrnpl | heterogeneous nuclear ribonucleoprotein L |
| Q9CVU5 | NA | 15388 | ENSMUSG00000015165 | Hnrnpl | heterogeneous nuclear ribonucleoprotein L |
| Q5BL18 | NA | 17184 | ENSMUSG00000037236 | Matr3 | matrin 3 |
| Q8K310 | NA | 17184 | ENSMUSG00000037236 | Matr3 | matrin 3 |
| Q544Z3 | NA | 15384 | ENSMUSG00000020358 | Hnrnpab | heterogeneous nuclear ribonucleoprotein A/B |
| Q99020 | NA | 15384 | ENSMUSG00000020358 | Hnrnpab | heterogeneous nuclear ribonucleoprotein A/B |
| O35737 | NA | 59013 | ENSMUSG00000007850 | Hnrnph1 | heterogeneous nuclear ribonucleoprotein H1 |
| Q811L7 | NA | 59013 | ENSMUSG00000007850 | Hnrnph1 | heterogeneous nuclear ribonucleoprotein H1 |
| Q61644 | NA | 23969 | ENSMUSG00000040276 | Pacsin1 | protein kinase C and casein kinase substrate in neurons 1 |
| Q543Y7 | NA | 23969 | ENSMUSG00000040276 | Pacsin1 | protein kinase C and casein kinase substrate in neurons 1 |
| Q6PFA2 | NA | 12757 | ENSMUSG00000028478 | Clta | clathrin, light polypeptide (Lca) |
| O88569 | NA | 53379 | ENSMUSG00000004980 | Hnrnpa2b1 | heterogeneous nuclear ribonucleoprotein A2/B1 |
| P49312 | NA | 15382 | ENSMUSG00000046434 | Hnrnpa1 | heterogeneous nuclear ribonucleoprotein A1 |
| Q9DB41 | NA | 71803 | ENSMUSG00000004902 | Slc25a18 | solute carrier family 25 (mitochondrial carrier), member 18 |
| Q544R5 | NA | 230908 | ENSMUSG00000041459 | Tardbp | TAR DNA binding protein |
| Q921F2 | NA | 230908 | ENSMUSG00000041459 | Tardbp | TAR DNA binding protein |
| Q91Z31 | NA | 56195 | ENSMUSG00000028134 | Ptbp2 | polypyrimidine tract binding protein 2 |
| P03995 | NA | 14580 | ENSMUSG00000020932 | Gfap | glial fibrillary acidic protein |
| Q6GT24 | NA | 11758 | ENSMUSG00000026701 | Prdx6 | peroxiredoxin 6 |
| O08709 | NA | 11758 | ENSMUSG00000026701 | Prdx6 | peroxiredoxin 6 |
| Q8VHM5 | NA | 74326 | ENSMUSG00000066037 | Hnrnpr | heterogeneous nuclear ribonucleoprotein R |
| Q3THA6 | NA | 225027 | ENSMUSG00000024097 | Sfrs7 | splicing factor, arginine/serine-rich 7 |
| Q8BL97 | NA | 225027 | ENSMUSG00000024097 | Sfrs7 | splicing factor, arginine/serine-rich 7 |
| Q9Z204 | NA | 15381 | ENSMUSG00000060373 | Hnrnpc | heterogeneous nuclear ribonucleoprotein C |
| Q5SX50 | NA | 18643 | ENSMUSG00000018293 | Pfn1 | profilin 1 |
| Q3U7V7 | NA | 18643 | ENSMUSG00000018293 | Pfn1 | profilin 1 |
| P62962 | NA | 18643 | ENSMUSG00000018293 | Pfn1 | profilin 1 |
| **cellular component----membrane-bounded organelle----GO:0043227** | | | | | |
| C=6833;O=30;E=17.87;R=1.68;rawP=0.0001;adjP=0.0006 | | | | | |
| Q60668 | NA | 11991 | ENSMUSG00000000568 | Hnrnpd | heterogeneous nuclear ribonucleoprotein D |
| O08539 | NA | 30948 | ENSMUSG00000024381 | Bin1 | bridging integrator 1 |
| Q3TT81 | NA | 18521 | ENSMUSG00000056851 | Pcbp2 | poly(rC) binding protein 2 |
| Q61990 | NA | 18521 | ENSMUSG00000056851 | Pcbp2 | poly(rC) binding protein 2 |
| Q9WV55 | NA | 30960 | ENSMUSG00000024091 | Vapa | vesicle-associated membrane protein, associated protein A |
| Q5BL18 | NA | 17184 | ENSMUSG00000037236 | Matr3 | matrin 3 |
| Q8K310 | NA | 17184 | ENSMUSG00000037236 | Matr3 | matrin 3 |
| Q3UMT7 | NA | 15388 | ENSMUSG00000015165 | Hnrnpl | heterogeneous nuclear ribonucleoprotein L |
| Q8R081 | NA | 15388 | ENSMUSG00000015165 | Hnrnpl | heterogeneous nuclear ribonucleoprotein L |
| Q9CVU5 | NA | 15388 | ENSMUSG00000015165 | Hnrnpl | heterogeneous nuclear ribonucleoprotein L |
| Q544Z3 | NA | 15384 | ENSMUSG00000020358 | Hnrnpab | heterogeneous nuclear ribonucleoprotein A/B |
| Q99020 | NA | 15384 | ENSMUSG00000020358 | Hnrnpab | heterogeneous nuclear ribonucleoprotein A/B |
| Q6PFA2 | NA | 12757 | ENSMUSG00000028478 | Clta | clathrin, light polypeptide (Lca) |
| Q61644 | NA | 23969 | ENSMUSG00000040276 | Pacsin1 | protein kinase C and casein kinase substrate in neurons 1 |
| Q543Y7 | NA | 23969 | ENSMUSG00000040276 | Pacsin1 | protein kinase C and casein kinase substrate in neurons 1 |
| O35737 | NA | 59013 | ENSMUSG00000007850 | Hnrnph1 | heterogeneous nuclear ribonucleoprotein H1 |
| Q811L7 | NA | 59013 | ENSMUSG00000007850 | Hnrnph1 | heterogeneous nuclear ribonucleoprotein H1 |
| O88569 | NA | 53379 | ENSMUSG00000004980 | Hnrnpa2b1 | heterogeneous nuclear ribonucleoprotein A2/B1 |
| Q8BWT1 | NA | 52538 | ENSMUSG00000036880 | Acaa2 | acetyl-Coenzyme A acyltransferase 2 (mitochondrial 3-oxoacyl-Coenzyme A thiolase) |
| P49312 | NA | 15382 | ENSMUSG00000046434 | Hnrnpa1 | heterogeneous nuclear ribonucleoprotein A1 |
| Q60749 | NA | 20218 | ENSMUSG00000028790 | Khdrbs1 | KH domain containing, RNA binding, signal transduction associated 1 |
| P70333 | NA | 56258 | ENSMUSG00000045427 | Hnrnph2 | heterogeneous nuclear ribonucleoprotein H2 |
| A2BDV8 | NA | 56258 | ENSMUSG00000045427 | Hnrnph2 | heterogeneous nuclear ribonucleoprotein H2 |
| Q9DB41 | NA | 71803 | ENSMUSG00000004902 | Slc25a18 | solute carrier family 25 (mitochondrial carrier), member 18 |
| Q544R5 | NA | 230908 | ENSMUSG00000041459 | Tardbp | TAR DNA binding protein |
| Q921F2 | NA | 230908 | ENSMUSG00000041459 | Tardbp | TAR DNA binding protein |
| Q9Z0H4 | NA | 14007 | ENSMUSG00000002107 | Cugbp2 | CUG triplet repeat, RNA binding protein 2 |
| Q9D2P8 | NA | 17433 | ENSMUSG00000032517 | Mobp | myelin-associated oligodendrocytic basic protein |
| Q564E6 | NA | 20823 | ENSMUSG00000068882 | Ssb | Sjogren syndrome antigen B |
| P32067 | NA | 20823 | ENSMUSG00000068882 | Ssb | Sjogren syndrome antigen B |
| Q91Z31 | NA | 56195 | ENSMUSG00000028134 | Ptbp2 | polypyrimidine tract binding protein 2 |
| Q6GT24 | NA | 11758 | ENSMUSG00000026701 | Prdx6 | peroxiredoxin 6 |
| O08709 | NA | 11758 | ENSMUSG00000026701 | Prdx6 | peroxiredoxin 6 |
| Q91VM5 | NA | 19656 | ENSMUSG00000037070 | Rbmxrt | RNA binding motif protein, X chromosome retrogene |
| Q8VHM5 | NA | 74326 | ENSMUSG00000066037 | Hnrnpr | heterogeneous nuclear ribonucleoprotein R |
| P61979 | NA | 15387 | NULL | Hnrnpk | heterogeneous nuclear ribonucleoprotein K |
| Q54A87 | NA | 66237 | ENSMUSG00000024403 | Atp6v1g2 | ATPase, H+ transporting, lysosomal V1 subunit G2 |
| Q9WTT4 | NA | 66237 | ENSMUSG00000024403 | Atp6v1g2 | ATPase, H+ transporting, lysosomal V1 subunit G2 |
| Q3THA6 | NA | 225027 | ENSMUSG00000024097 | Sfrs7 | splicing factor, arginine/serine-rich 7 |
| Q8BL97 | NA | 225027 | ENSMUSG00000024097 | Sfrs7 | splicing factor, arginine/serine-rich 7 |
| Q9Z204 | NA | 15381 | ENSMUSG00000060373 | Hnrnpc | heterogeneous nuclear ribonucleoprotein C |
| Q5SX50 | NA | 18643 | ENSMUSG00000018293 | Pfn1 | profilin 1 |
| Q3U7V7 | NA | 18643 | ENSMUSG00000018293 | Pfn1 | profilin 1 |
| P62962 | NA | 18643 | ENSMUSG00000018293 | Pfn1 | profilin 1 |
| A7UQY4 | NA | 17876 | NULL | Myef2 | myelin basic protein expression factor 2, repressor |
| Q8C854 | NA | 17876 | NULL | Myef2 | myelin basic protein expression factor 2, repressor |
| **cellular component----intracellular----GO:0005622** | | | | | |
| C=9293;O=36;E=24.30;R=1.48;rawP=6.74e-05;adjP=0.0006 | | | | | |
| Q3TT81 | NA | 18521 | ENSMUSG00000056851 | Pcbp2 | poly(rC) binding protein 2 |
| Q61990 | NA | 18521 | ENSMUSG00000056851 | Pcbp2 | poly(rC) binding protein 2 |
| Q9WV55 | NA | 30960 | ENSMUSG00000024091 | Vapa | vesicle-associated membrane protein, associated protein A |
| Q8BWT1 | NA | 52538 | ENSMUSG00000036880 | Acaa2 | acetyl-Coenzyme A acyltransferase 2 (mitochondrial 3-oxoacyl-Coenzyme A thiolase) |
| Q60749 | NA | 20218 | ENSMUSG00000028790 | Khdrbs1 | KH domain containing, RNA binding, signal transduction associated 1 |
| P70333 | NA | 56258 | ENSMUSG00000045427 | Hnrnph2 | heterogeneous nuclear ribonucleoprotein H2 |
| A2BDV8 | NA | 56258 | ENSMUSG00000045427 | Hnrnph2 | heterogeneous nuclear ribonucleoprotein H2 |
| O88737 | NA | 12217 | ENSMUSG00000032589 | Bsn | bassoon |
| Q3UXD6 | NA | 12217 | ENSMUSG00000032589 | Bsn | bassoon |
| P23242 | NA | 14609 | ENSMUSG00000050953 | Gja1 | gap junction protein, alpha 1 |
| Q8C4N2 | NA | 14609 | ENSMUSG00000050953 | Gja1 | gap junction protein, alpha 1 |
| Q9Z0H4 | NA | 14007 | ENSMUSG00000002107 | Cugbp2 | CUG triplet repeat, RNA binding protein 2 |
| Q9D2P8 | NA | 17433 | ENSMUSG00000032517 | Mobp | myelin-associated oligodendrocytic basic protein |
| Q564E6 | NA | 20823 | ENSMUSG00000068882 | Ssb | Sjogren syndrome antigen B |
| P32067 | NA | 20823 | ENSMUSG00000068882 | Ssb | Sjogren syndrome antigen B |
| Q91VM5 | NA | 19656 | ENSMUSG00000037070 | Rbmxrt | RNA binding motif protein, X chromosome retrogene |
| P61979 | NA | 15387 | NULL | Hnrnpk | heterogeneous nuclear ribonucleoprotein K |
| Q54A87 | NA | 66237 | ENSMUSG00000024403 | Atp6v1g2 | ATPase, H+ transporting, lysosomal V1 subunit G2 |
| Q9WTT4 | NA | 66237 | ENSMUSG00000024403 | Atp6v1g2 | ATPase, H+ transporting, lysosomal V1 subunit G2 |
| A7UQY4 | NA | 17876 | NULL | Myef2 | myelin basic protein expression factor 2, repressor |
| Q8C854 | NA | 17876 | NULL | Myef2 | myelin basic protein expression factor 2, repressor |
| O08539 | NA | 30948 | ENSMUSG00000024381 | Bin1 | bridging integrator 1 |
| Q60668 | NA | 11991 | ENSMUSG00000000568 | Hnrnpd | heterogeneous nuclear ribonucleoprotein D |
| Q3UMT7 | NA | 15388 | ENSMUSG00000015165 | Hnrnpl | heterogeneous nuclear ribonucleoprotein L |
| Q8R081 | NA | 15388 | ENSMUSG00000015165 | Hnrnpl | heterogeneous nuclear ribonucleoprotein L |
| Q9CVU5 | NA | 15388 | ENSMUSG00000015165 | Hnrnpl | heterogeneous nuclear ribonucleoprotein L |
| Q5BL18 | NA | 17184 | ENSMUSG00000037236 | Matr3 | matrin 3 |
| Q8K310 | NA | 17184 | ENSMUSG00000037236 | Matr3 | matrin 3 |
| Q544Z3 | NA | 15384 | ENSMUSG00000020358 | Hnrnpab | heterogeneous nuclear ribonucleoprotein A/B |
| Q99020 | NA | 15384 | ENSMUSG00000020358 | Hnrnpab | heterogeneous nuclear ribonucleoprotein A/B |
| O35737 | NA | 59013 | ENSMUSG00000007850 | Hnrnph1 | heterogeneous nuclear ribonucleoprotein H1 |
| Q811L7 | NA | 59013 | ENSMUSG00000007850 | Hnrnph1 | heterogeneous nuclear ribonucleoprotein H1 |
| Q61644 | NA | 23969 | ENSMUSG00000040276 | Pacsin1 | protein kinase C and casein kinase substrate in neurons 1 |
| Q543Y7 | NA | 23969 | ENSMUSG00000040276 | Pacsin1 | protein kinase C and casein kinase substrate in neurons 1 |
| Q6PFA2 | NA | 12757 | ENSMUSG00000028478 | Clta | clathrin, light polypeptide (Lca) |
| O88569 | NA | 53379 | ENSMUSG00000004980 | Hnrnpa2b1 | heterogeneous nuclear ribonucleoprotein A2/B1 |
| P49312 | NA | 15382 | ENSMUSG00000046434 | Hnrnpa1 | heterogeneous nuclear ribonucleoprotein A1 |
| Q9DB41 | NA | 71803 | ENSMUSG00000004902 | Slc25a18 | solute carrier family 25 (mitochondrial carrier), member 18 |
| Q544R5 | NA | 230908 | ENSMUSG00000041459 | Tardbp | TAR DNA binding protein |
| Q921F2 | NA | 230908 | ENSMUSG00000041459 | Tardbp | TAR DNA binding protein |
| Q91Z31 | NA | 56195 | ENSMUSG00000028134 | Ptbp2 | polypyrimidine tract binding protein 2 |
| Q52L78 | NA | 12955 | ENSMUSG00000032060 | Cryab | crystallin, alpha B |
| P23927 | NA | 12955 | ENSMUSG00000032060 | Cryab | crystallin, alpha B |
| P03995 | NA | 14580 | ENSMUSG00000020932 | Gfap | glial fibrillary acidic protein |
| Q6GT24 | NA | 11758 | ENSMUSG00000026701 | Prdx6 | peroxiredoxin 6 |
| O08709 | NA | 11758 | ENSMUSG00000026701 | Prdx6 | peroxiredoxin 6 |
| Q8VHM5 | NA | 74326 | ENSMUSG00000066037 | Hnrnpr | heterogeneous nuclear ribonucleoprotein R |
| Q9JII6 | NA | 58810 | ENSMUSG00000028692 | Akr1a4 | aldo-keto reductase family 1, member A4 (aldehyde reductase) |
| Q540D7 | NA | 58810 | ENSMUSG00000028692 | Akr1a4 | aldo-keto reductase family 1, member A4 (aldehyde reductase) |
| Q8VEK3 | NA | 51810 | ENSMUSG00000039630 | Hnrnpu | heterogeneous nuclear ribonucleoprotein U |
| Q3THA6 | NA | 225027 | ENSMUSG00000024097 | Sfrs7 | splicing factor, arginine/serine-rich 7 |
| Q8BL97 | NA | 225027 | ENSMUSG00000024097 | Sfrs7 | splicing factor, arginine/serine-rich 7 |
| Q9Z204 | NA | 15381 | ENSMUSG00000060373 | Hnrnpc | heterogeneous nuclear ribonucleoprotein C |
| Q5SX50 | NA | 18643 | ENSMUSG00000018293 | Pfn1 | profilin 1 |
| Q3U7V7 | NA | 18643 | ENSMUSG00000018293 | Pfn1 | profilin 1 |
| P62962 | NA | 18643 | ENSMUSG00000018293 | Pfn1 | profilin 1 |
